# Effectiveness and safety of selective internal radiation therapy using yttrium-90 glass microspheres for hepatocellular carcinoma: real-world results from the multi-center prospective PROACTIF cohort of 989 patients

**DOI:** 10.1016/j.eclinm.2026.103884

**Published:** 2026-04-17

**Authors:** Boris Guiu, Clément Bailly, Eric Vibert, Ghoufrane Tlili, Denis Mariano-Goulart, Julien Edeline, Yann Touchefeu, Emmanuel Durand, Jean Frédéric Blanc, Julia Chalaye, Hélène Regnault, Antoine Bouvier, Geraldine Sergent, Christian Sengel, Stéphane Renaud, Agnès Rode, Claude Somma, Patrick Chevallier, Vincent Habouzit, Isabelle Brenot-Rossi, Anthony Dohan, Lambros Tselikas, Thierry DeBaère, Sylvain Manfredi, Arnaud Dieudonné, Kirk Fowers, Eveline Boucher, Binal Patel, Eric Vicaut, Etienne Garin, The PROACTIF Investigators Group, Emmanuel Durand, Olivier Meyrignac, Clara Prud'homme, Gilles Grimon, Anthony Dohan, Maxime Barat, Raphael Dautry, Julia Chalaye, Hélène Regnault, Hicham Kobeiter, Haytham Derbel, Eric Vicaut, Eric Vibert, Olivier Rosmorduc, Anton Pachev, Thomas Aparicio, Laetitia Vercellino, Etienne Garin, Julien Edeline, Yann Rolland, Julien Farce, Samuel Lesourd, Xavier Palard-Novello, Inna Dygai-Cochet, François Godard, Romain Popoff, Julie Pellegrinelli, Arnaud Dieudonné, Stéphanie Becker, Stéphane Renaud, Aymeric Guibal, Mohamed Abdel-Rehim, Faiza Khemissa Akouz, Sibel Isal, Charles Mastier, Arnaud Muller, Guillaume Guthier, Paul Calame, Hatem Boulahdour, Vincent Di Martino, Elodie Chevalier, Valérie Laurent, Xavier Orry, Thierry Yzet, Eric Nguyen-Khac, Vandici Ovidiu-Florian, Chalabia Fergani, Antoine Bouvier, Pacome Fosse, Frederic Oberti, Ghoufrane Tlili, Jean Frédéric Blanc, Panteleimon Papadopoulos, Ronan Abral, Antoine Boizet, Jean Romain Risson, Sylvain Manfredi, Francois Ghiringhelli, Romaric Loffroy, Michel Greget, Fabrice Hubele, Claude Somma, Olivier Durieux, Géraldine Sergent, Clio Baillet, Massih Ningarhari, Christian Sengel, Julie Roux, Julien Ghelfi, Thomas Decaens, Clément Bailly, Yann Touchefeu, Frederic Douane, Matthieu Barbaud, Patrick Chevallier, Mohamed El Zibawi, Philippe Viaud, Micheline Razzouk, Jean Goupil, Melanie Sainmont, Jean-Marie Peron, Philippe Otal, Fatima-Zohra Mokrane, David Sefrioui, Frederic Di Fiore, Vincent Habouzit, Remi Grange, Jean Marc Phelip, Jean-Pierre Tasu, David Tougeron, Rémy Perdrisot, Isabelle Brenot-Rossi, Gilles Piana, Brice Chanez, Daniel Ouk, Clément Mennetrey, Franck Grillet, Amine Bouhamama, Sandrine Parisse-Di Martino, Marco Dioguardi Burgio, Maxime Ronot, Rachida Lebtahi, Mohammed Bouattour, Laurent Milot, Jérôme Dumortier, Boris Guiu, Denis Mariano-Goulart, Eric Assenat, Carole Allimant, Christine Latry Kuhn, Marjolaine Fourcade, Agnès Rode, Philippe Merle, Jean Palussière, Vincent Prega-Renaud, Thierry De Baère, Lambros Tselikas, Iulian Enescu

**Affiliations:** a Hôpital Saint-Eloi, Montpellier, France; b Centre Hospitalier Universitaire de Nantes, Nantes, France; c Assistance Publique-Hôpitaux de Paris, Hôpital Paul Brousse, Villejuif, France; d Centre Hospitalier Universitaire de Bordeaux, Hôpital du Haut-Lévêque, Bordeaux, France; e Centre Eugène Marquis, Rennes, France; f Assistance Publique-Hôpitaux de Paris, Hôpital Bicêtre, Le Kremlin-Bicêtre, France; g Assistance Publique-Hôpitaux de Paris, Hôpital Henri-Mondor, Créteil, France; h Centre Hospitalier Universitaire de Angers, Angers, France; i Centre Hospitalier Universitaire de Lille, Hôpital Claude Huriez, Lille, France; j Centre Hospitalier Universitaire de Michallon, Grenoble, France; k Centre Hospitalier de Perpignan, Perpignan, France; l Hospices Civils de Lyon, Hôpital de la Croix Rousse, Lyon, France; m Centre Hospitalier Universitaire de la Timone, Hôpital Adultes, Marseille, France; n Centre Hospitalier Universitaire de Nice, Nice, France; o Centre Hospitalier Universitaire de Saint-Étienne, Hôpital Nord, Saint-Étienne, France; p CLCC Institut Paoli-Calmettes, Marseille, France; q Assistance Publique-Hôpitaux de Paris, Hôpital Cochin, Paris, France; r Institut Gustave Roussy, Villejuif, France; s Centre Hospitalier Universitaire de Dijon, Université Bourgogne Europe, Dijon, France; t Centre Henri Becquerel, Rouen, France; u Boston Scientific Corporation, Malborough, MA, USA; v Assistance Publique-Hôpitaux de Paris, Hôpital Lariboisière – Fernand-Widal, Paris, France

**Keywords:** SIRT, TARE, Y90, HCC, Real-world evidence

## Abstract

**Background:**

Selective Internal Radiation Therapy with yttrium-90 (Y90) has been used for decades, yet guideline recommendations remain inconsistent. High-quality real-world data is needed to guide practice. The objectives of this study were to evaluate effectiveness, safety, and patient quality of life (QoL) with TheraSphere™ treatment in real-world clinical practice, and to identify clinical and dosimetric factors associated with survival.

**Methods:**

PROACTIF was a prospective, open label, non-interventional, all-comers cohort study that recruited patients who received Y90 glass microspheres (TheraSphere™) per local standard of care across 34 French sites (January 2019–January 2024). Co-primary endpoints were overall survival (OS) and QoL. Secondary endpoints included safety, conversion to surgery, and factors associated with OS. OS and time-to-deterioration in QoL (Functional Assessment of Cancer Therapy-Hepatobiliary) were assessed by Kaplan–Meier analysis. Adverse events were descriptively summarized using Common Terminology Criteria for Adverse Events, version 5. Trial registration: ClinicalTrials.gov Identifier, NCT04069468.

**Findings:**

Amongst 989 HCC patients, 13·3%/18·9%/57·9%/5·8% were Barcelona Clinic Liver Cancer (BCLC) A/B/C/D, respectively; 35·3% had portal-vein tumor thrombosis (PVT); 74·4% were treated using multicompartment dosimetry, and 53·6% with selective Y90 administration. Mean index lesion dose was 435·4 Gy. For all patients, median OS (mOS) [95% CI] was 21·8 months (M) [20·1–23·3]. mOS was 27·0 M [20·7–31·4] for BCLC B, 21·1 M [18·0–22·8] for BCLC C; 23·1 M [21·6–27·0] without PVT, 24·8 M [19·3–30·3] for patients with Vp1/Vp2; 16·8 M mOS for patients with a pretreatment absorbed dose to the index lesion <200 Gy versus 26·0 M with ≥200 Gy (p < 0·001); 19·7 M for <400 Gy and 30·7 M for ≥400 Gy (p < 0·001). After Y90, 106/989 (10·7%) underwent curative-intent surgery, resulting in a mOS of 48·6 M [40·6-not evaluable], versus 20·1 M [17·7–21·7] without surgery. Median time-to-deterioration in QoL was 10·6 M [9·6–11·7]. Serious adverse events occurred in 7·5% patients; serious treatment-related events in 3·7%.

**Interpretation:**

In this large real-world cohort, treatment with Y90 glass microspheres demonstrated favorable effectiveness and safety with meaningful outcomes, especially in PVT patients and following subsequent surgery. PROACTIF showed a strong dose-survival relationship as demonstrated in previous studies, and highlights the potential of a tumor absorbed dose ≥400 Gy to further increase survival. These findings support dosimetry-guided Y90 across all BCLC stages, and should inform future guideline recommendations.

**Funding:**

Boston Scientific Corporation.

**Research in context**

**Evidence before this study**

We searched PubMed for articles published in English between January 2000 and November 1, 2025, focusing on randomized studies in hepatocellular carcinoma (HCC), and using terms “selective internal radiation therapy” or “SIRT”, “radioembolization”, or “transarterial radioembolization” or “TARE”, “90Y” or “Y90”, AND “chemoembolization”, “sorafenib”, or “immunotherapy” for the comparator. In early/intermediate stages (BCLC A/B), two phase II randomized studies (PREMIERE and TRACE) compared SIRT with chemoembolization or drug-eluting beads; SIRT demonstrated improved time-to-progression in both studies, and improved overall survival in TRACE. Despite these findings, chemoembolization remains the standard of care for intermediate HCC in most clinical recommendations. In advanced-stage patients (mainly BCLC C), three negative trials compared SIRT (without personalized dosimetry) with sorafenib (then the standard of care). DOSISPHERE-01, a positive phase II trial, showed that focusing on delivery of a threshold absorbed dose to target tumor(s) (i.e, personalized dosimetry) markedly improved outcomes versus providing an average dose of 120 Gy to the perfused volume including the tumor(s) (i.e., standard dosimetry), resulting in a 0·41 hazard ratio for overall survival, thereby establishing personalized dosimetry as the standard for SIRT and calling into question the relevance of earlier negative trials performed without this approach. Since 2020—when immunotherapy-based regimens supplanted sorafenib as the standard of care for advanced HCC—no randomized trial has compared SIRT (alone or combined with immunotherapy) against immunotherapy alone. Over the same period, at least five phase III randomized controlled trials (RCTs) of immunotherapy-based combinations (IMbrave150, HIMALAYA, ORIENT-32, CARES-310, and CheckMate 9DW) have demonstrated superiority to sorafenib or lenvatinib.

A key limitation of current evidence is that RCTs of SIRT are constrained by substantial methodological and ethical issues. Unless [^99m^Tc]Tc-MAA scintigraphy screening is performed prior to randomization, an imbalance would occur between treatment arms due to ineligibility resulting from either excessive lung shunt, gastrointestinal shunting, poor tumor targeting, or inadequate dosimetry that could lead to decreased efficacy. Furthermore, patient enrollment may be adversely impacted in the context of a known dose–response (theranostic) relationship for SIRT, anticipating a treatment benefit, raises potential ethical and practical concerns. These constraints hinder the generation of classical evidence-based data and highlight the importance for large, high-quality real-world datasets.

**Added value of this study**

This study represents the largest prospective and multicenter evaluation of HCC patients treated with SIRT using glass microspheres in a real-world setting. Treatment decisions were made in multidisciplinary tumor boards and treatment was performed according to local practice. This all-comers study shows that, with appropriate patient selection, and broad adoption of personalized dosimetry-based treatment, meaningful survival benefit can be achieved across all BCLC stages including patients with PVT. Prolonged survival was observed with increasing tumor dose, and in patients who were subsequently suitable for surgery.

**Implications of all the available evidence**

Findings from this large, rigorously conducted real-world study reinforce the central importance of patient selection and treatment method, particularly dosimetry-guided treatment delivery, consistent with prior reports. PROACTIF provides contemporary real-world evidence that will inform future treatment decisions and guideline recommendations.

## Introduction

Selective internal radiation therapy (SIRT) with yttrium-90 (Y90) glass microspheres (TheraSphere™) has been used to treat liver malignancies in Europe for over two decades, and hepatocellular carcinoma (HCC) in the United States (US) for more than 25 years. SIRT provides durable local control with an acceptable safety profile across disease stages.^1^ Contemporary guidelines, Barcelona Clinic of Liver Cancer (BCLC), European Society for Medical Oncology, and European Association for the Study of the Liver, consider SIRT as a treatment option for very-early and early-stage HCC in patients who are not candidates for resection or transarterial chemoembolization (TACE),^2^, ^3^, ^4^, ^5^ These guidelines remain cautious or restrictive regarding SIRT in patients with intermediate/advanced disease and patients with portal vein thrombosis (PVT), despite accumulating data supporting Y90 use.

Initially, Y90 glass microspheres were used with a single compartment dosimetry (SCD) approach that targeted 120 Gy to the perfused volume (usually a liver lobe), termed “standard dosimetry”. Over time, the use of Y90 SIRT has evolved toward a more personalized treatment strategy, focusing on appropriate tumor/perfused volume dosing, which demonstrated to be associated with improved tumor response and overall survival.^1^^,^^6^, ^7^, ^8^ Personalized treatment is treatment with a pre-defined objective (i.e., sustained tumor control, complete response, downstaging, bridge to surgery) and can be achieved through two different dosimetry methods: SCD and multicompartment dosimetry (MCD). The SCD method is simple and is typically chosen for selective treatment of a limited tumor volume (≤25% of the liver). Selective high dose treatment, referred to as radiation segmentectomy, targets a perfused liver dose ≥400 Gy with the restriction of ≤25% of perfused liver in patients with good liver function (ALBI 1) and ≤15% of perfused liver for patients with ALBI 2.^9^ The DOSISPHERE-01 study demonstrated the importance of pretreatment MCD determination, the tumoral dose/response and survival relationship, and recommended threshold tumor doses.^8^ Using MCD, the dose to the tumor and dose to the normal perfused liver are evaluated using the technetium-99 m macroaggregated albumin ([^99m^Tc]TcMAA) scan, with the objective to deliver as high of a dose as possible (e.g., ≥300 Gy; upper limit not established) while limiting the dose received by the non-tumoral liver tissue (e.g., <120 Gy to the normal perfused liver when the hepatic reserve is <30%).^10^ Current best practice includes [^99m^Tc]TcMAA–based MCD, particularly for large lesions^7^; or SCD using a more selective catheter positioning to spare non-tumoral parenchyma.^1^ However, there are limited prospective data in routine clinical practice that simultaneously capture the treatment goals and methods, the effectiveness of SIRT, patient-reported outcomes, safety, and key clinical endpoints such as downstaging/bridging to curative therapy.

Randomized controlled trials of SIRT pose specific methodological challenges. Without pre-randomization [^99m^Tc]TcMAA angiography/scintigraphy in both arms, imbalances are likely because a high fraction of patients prove ineligible for SIRT (e.g., excessive lung or gastrointestinal shunting, poor tumor targeting, or inadequate dosimetry). Such patients represented 19% in the DOSISPHERE-01 randomized study.^8^ This screening may impede accrual. Moreover, given the theranostic, dose–response relationship of SIRT, assigning patients to systemic therapy alone when SIRT benefit is reasonably anticipated raises potential ethical and practical concerns. Collectively, these constraints limit the feasibility of conventional randomized controlled trials (RCTs) and underscore the need for large, high-quality real-world datasets.

PROACTIF (Prospective, Post-Approval, Multiple Centre, Open-Label, Non-Interventional, Registry Study to Evaluate Effectiveness of TheraSphere™ in Clinical Practice in France) was a multi-center, prospective post-approval registry mandated by the French health authority to support continued reimbursement of Y90 glass microspheres for hepatic malignancies. Herein are reported outcomes for patients with HCC who were treated between 2019 and 2024 across 34 centers, with prespecified collection of clinical, biological, dosimetry, survival and quality-of-life (QoL) data. The objectives were to evaluate effectiveness, safety, and patient QoL with TheraSphere treatment in real-world clinical practice, and to identify clinical and dosimetric factors associated with survival.

## Methods

### Study design and patient population

PROACTIF was a prospective, post-approval, multicenter, single arm, open label, observational registry.^11^ Consecutive HCC patients treated with Y90 glass microspheres (TheraSphere™, Boston Scientific, Massachusetts, USA), who agreed to data collection were enrolled across 34 French centers (Supplementary Figure S1). The protocol was approved by the national ethics committee (N_IDRCB:2017-A01003-50). Trial registration: ClinicalTrials.gov Identifier, NCT04069468.

### Ethical approval

All procedures performed in studies involving human participants were in accordance with the ethical standards of the institutional and/or national research committee and with the 1964 Helsinki declaration and its later amendments or comparable ethical standards.

This study was classified in France as Recherche Impliquant la Personne Humaine de Catégorie 3 (RIHP 3) by the Agence Nationale de Sécurité du Médicament et des Produits de Santé (ANSM) due to its observational, non-interventional design. The protocol was approved upon random assignment to one of the 39 Independent Ethics Committees in France prior to the study's initiation (assigned to French Ethics Committee « Ile de France VII »); assignments are made by the French National Commission for Research Involving Human Persons (CNRIPH).

### Informed consent

No written consent was required for this post-approval non-interventional registry study, but only collection of non-opposition to data collection was mandatory and non-opposition has been obtained from all patients. Patients received a Patient Information Sheet (PIS), which they review prior to treatment and are given the opportunity to ask the investigator or delegate any questions. As detailed by the General Data Protection Regulation (GDPR) and requested by the ethics committee, this PIS informs the patient about the purpose and aim of the registry and how their personal medical data will be used. After reviewing, clinicians document whether the patient expressed verbal non-opposition to data collection in the patient's record (“non-opposition to data collection”).

### Consent for publication

Consent for publication was obtained for every individual person's data included in the study.

### Procedures

Treatment decisions were made by local multidisciplinary tumor boards. Treatment planning used angiography with [^99m^Tc]TcMAA administration single-photon emission computed tomography (SPECT)-computed tomography (CT) imaging, and treatment administration followed local procedures with contemporary treatment recommendations and guidelines, including personalized dosimetry.^8^^,^^12^ Y90 treatment administration type was categorized as selective (sub-segmental, segmental, or sectorial) or non-selective (lobar). Briefly, recommendations for ablative selective administrations were performed using the SCD method and targeting ≥400 Gy to the perfused volume. Lobar administrations were performed using MCD and targeting ≥300 Gy to the tumor while respecting dose to normal tissue (i.e., <120 Gy to the normal perfused liver when hepatic reserve is <30%).^10^

### Data collection

Data collection has been previously described.^11^ Briefly, at baseline and treatment visits, patients were assessed for disease characteristics, liver function, QoL, Eastern Cooperative Oncology Group (ECOG), laboratory values, TheraSphere treatment planning and administration details (pretreatment and treatment angiography, pretreatment and post-treatment dosimetry). After treatment, the following data were collected at routine follow-up visits every 2–4 M for 12 months: clinical, biological, laboratory values; adverse events (AEs) that occurred up to 90 days, serious adverse events (SAEs) that occurred up to 12 M; occurrence of clinical and/or biological liver decompensation; QoL questionnaire results (FACT-Hep); ECOG; tumor response, subsequent TheraSphere treatment details, subsequent anticancer treatment; and survival status. After 12 months, more limited data was collected: QoL, ECOG, subsequent treatment, treatment-related SAEs, and survival status until study end, death, study withdrawal, or were lost to follow-up. Tumor response was reported by investigators per Response Evaluation Criteria in Solid Tumors 1·1 (RECIST 1·1) or modified Response Evaluation Criteria in Solid Tumors (mRECIST) response criteria. Baseline CT or MRI, [^99m^Tc]TcMAA SPECT-CT, Y90 SPECT-CT, or Y90 PET-CT were collected for central dosimetry review. BCLC stage was programmatically derived using the following patient data documented by the investigator: ECOG, liver function (Child-Pugh, albumin-bilirubin score and grade [ALBI]), extrahepatic spread, tumor number, and PVT. Of note, patients were classified as BCLC D if ECOG was 3 or 4 or if liver function was impaired. Database lock was 22 MAY 2025.

### Outcome measures

The primary outcomes were overall survival (OS) and QoL. OS was defined as the date of Y90 treatment to death from any cause or last date known alive. OS was not censored after subsequent treatment. QoL was assessed by Functional Assessment of Cancer Therapy-Hepatobiliary [FACT-Hep] questionnaire. Completion of the QoL questionnaire was not mandatory. QoL deterioration was defined as a 7-point decrease in the global QoL score or death. For time-to-deterioration in QoL, patients were censored at the last assessment prior to receiving subsequent treatment. Secondary outcomes included tumor response assessments, the identification of patient and treatment factors associated with survival, safety, and subsequent treatments (type and timing). Best tumor response was analyzed per RECIST 1·1 or mRECIST, according to what was reported by the investigator. Safety assessments included the collection of (i) treatment-related grade ≥3 AEs within 90 days of last treatment; (ii) any SAEs within 12 M; (iii) device-related SAEs until study end; (iv) hospitalizations up to 30 days; and (v) occurrence of liver decompensation. AEs were graded using the National Cancer Institute- Common Terminology Criteria for Adverse Events, version 5 (NCI-CTCAE v5). An event was classified as treatment-emergent if it occurred after Y90 administration; treatment-related event if the causality assessment per investigator was due to the device and/or procedure; device-related if the causality assessment per investigator was due to the device. Liver decompensation was defined as the occurrence of grade ≥2 of any of the following: ascites, elevated bilirubin, portal hypertension leading to gastrointestinal bleeding, edema, hypoalbuminemia, and/or encephalopathy. Post-treatment dosimetry and a comparison of pretreatment and post-treatment dosimetry will be reported elsewhere in a dedicated dosimetry publication.

### Statistical analysis

Continuous variables (e.g., hospitalization duration, dose) were summarized as mean and standard deviation (SD), or median and interquartile range (IQR). Categorical data were summarized with observed counts and percent totals. Median study duration and follow-up was estimated by reverse Kaplan–Meier method. Kaplan–Meier analysis was performed for time-to-event endpoints (OS, time-to-deterioration of QoL [TTD QoL]), with the median and 95% confidence intervals (CI) reported using log–log standard error.^13^ Kaplan–Meier rates were calculated using the log–log transformation.^14^ Post-hoc analyses were performed to compare median OS by dose to the index lesion (<200 Gy versus ≥200 Gy, and <400 Gy versus ≥400 Gy), and by tumor size and tumor absorbed dose. A post-hoc log-rank analysis p-value was calculated to compare the median OS by BCLC, PVT, subsequent treatment. In addition, supplementary survival analyses were performed on Child-Pugh and ECOG subgroups to assess more granularity within each category (e.g., Child-Pugh B7 versus > B7, ECOG 1, and ECOG 2).

Univariable and multivariable Cox regression analyses were performed to assess the relationship between predictor variables and time-to-event endpoints. All covariates in the univariable models with a two-sided p-value <0·15 were assessed for collinearity using variance inflation factor (VIF) prior to being included in the multivariable analysis.^15^ In a step-wise approach, any covariate with the highest VIF was removed and this was repeated until all covariates had a VIF <5. Absorbed dose (AD) to index lesion (lesion with the longest diameter) and AD to total perfused tumor, along with their respective dose volume histogram values were collected, however, only AD to index lesion was used in the multivariable model as AD to total perfused tumor was available for fewer patients. Subgroups assessed by investigator included age group, unilobar versus bilobar disease extension, ECOG status, ALBI grade, disease etiology, liver status (cirrhosis versus no cirrhosis), Child-Pugh score, prior systemic therapy, prior transarterial TACE, treatment administration type (selective versus non-selective administration), subsequent surgery, and dosimetry method (SCD versus MCD); programmatically-derived BCLC stage at baseline was included; subgroups by central assessment included index lesion size, number of tumors, PVT classification, and dosimetry results (volume and AD).

### Role of the funding source

This study was sponsored by Boston Scientific Corporation. The sponsor designed the study, was involved in data collection, data analysis, data interpretation, and manuscript writing. The corresponding author had full access to all study data and had final responsibility for the decision to submit for publication.

## Results

Patients were recruited between January 2019 and January 2024 in 34 centers in France. The study included 1259 patients with liver malignancies who received Y90 glass microspheres; 989 had HCC which formed the treatment cohort for this publication. Median age was 71·0 years (with 33% ≥ 75); 89·9% were male; 57·4% had an ECOG performance status of 0 (Table 1). Most patients had liver cirrhosis or fibrosis (80·7%), mainly related to alcohol intake (47·5%) or viral hepatitis (24·8%), and were Child-Pugh A (81·7%). At baseline, 49·0% had a solitary tumor, and 77·1% had unilobar disease; median index lesion size was 6·8 cm (IQR, 4·6–9·0) by mRECIST. PVT was present in 35·3% of patients, and 4·6% had extrahepatic disease. Programmatically derived BCLC stage was A (13·3%), B (18·9%), C (57·9%), and D (5·8%). Notably, 68·9% (n = 91) of BCLC A tumors, 59·9% (n = 112) B, 63·9% (n = 366) C, and 61·4% (n = 35) D index tumors were >5 cm. Before SIRT, 39·2% of patients had received at least one prior anti-cancer treatment (Table 1).

**Table 1:** Patient demographics, tumor characteristics, and prior treatment.

	N (%)
Age, median (years)^a^	71·0
≥18–<65	260 (26·3)
≥65–<75	401 (40·5)
≥75	328 (33·2)
Gender^a^	–
Male	889 (89·9)
Female	100 (10·1)
Child-Pugh^a^	–
A5/A6	618 (62·5)/190 (19·2)
B7	47 (4·8)
>B7	21 (2·1)
Unknown/Missing	113 (11·4)
ALBI Grade^a^	–
1	380 (38·4)
2/3	440 (44·5)/15 (1·5)
Unknown/Missing	154 (15·6)
ECOG^a^	–
0	568 (57·4)
>0	349 (35·3)
1/2	329 (33·3)/15 (1·5)
3/4	3 (0·3)/2 (0·2)
Unknown/Missing	72 (7·3)
Liver status^a^	–
No history of liver disease	138 (14·0)
Liver cirrhosis	690 (69·8)
Liver fibrosis	108 (10·9)
Unknown/Missing	53 (5·4)
Disease Extent^a^	–
Unilobar	763 (77·1)
Bilobar	184 (18·6)
Unknown/Missing	42 (4·2)
Disease Etiology^a^, ^d^ (top 5)	–
Alcohol	470 (47·5)
Hepatitis B/C	67 (6·8)/178 (18·0)
No underlying disease	168 (17·0)
Metabolic/MASH	120 (12·1)/108 (10·9)
Comorbidities Present^a^, ^d^ (top 5)	–
Alcohol intake sequelae	536 (54·2)
Arterial hypertension	536 (54·2)
Diabetes	421 (42·6)
Smoking	381 (38·5)
Other previous or ongoing disease	144 (14·6)
Extrahepatic disease^a^	–
Yes	45 (4·6)
No	907 (91·7)
Missing or not assessed	37 (3·7)
BCLC Stage^b^	–
A	132 (13·3)
B	187 (18·9)
C	573 (57·9)
D	57 (5·8)
Unknown/missing	40 (4·0)
Number of lesions^c^	–
1	485 (49·0)
>1	484 (48·9)
Unknown/Missing	20 (2·0)
Index Lesion Diameter^c^ (mRECIST)	–
Median size, cm (IQR)	6·80 (4·6–9·0)
≤5 cm/>5 cm	294 (31·8)/631 (68·2)
≤7 cm/>7 cm	517 (55·9)/408 (44·1)
Unknown/Missing	64 (6·5)
Liver Tumor Burden^c^	–
<25%	606 (61·3)
≥25%	128 (12·9)
Unknown/Missing	255 (25·8)
PVT^c^	–
Yes	349 (35·3)
Vp1	75 (21·5)
Vp2	104 (29·8)
Vp3	138 (39·5)
Vp4	32 (9·2)
No	623 (63·0)
Unknown/Missing	17 (1·7)
Prior Anti-cancer Treatment	–
Yes^d^	388 (39·2)
Prior Locoregional Treatment^d^	282 (28·5)
Prior Systemic Treatment^d^	124 (12·5)
Prior Liver Surgery^d^	62 (6·3)

Abbreviations: ALBI, albumin-bilirubin; BCLC, Barcelona Clinic Liver Cancer; ECOG, Eastern Cooperative Oncology Group; IQR, interquartile range; MASH, metabolic dysfunction-associated steatohepatitis; mRECIST, modified Response Evaluation Criteria in Solid Tumors; PVT, portal vein thrombosis.

a Assessment by investigator.

b Programmatically derived using data entered by investigator.

c Assessment by central review.

d Patients may have had more than one.

Overall, 89% of patients were treated using personalized dosimetry (i.e., radiation segmentectomy, MCD). Target AD to index lesion or perfused volume and corresponding activity were determined by investigators using MCD in 736 of 989 (74·4%) or SCD in 251 of 989 (25·4%). Y90 administration was selective for 53·6% of patients, non-selective for 43·0%, or a mix of both for 2·4% (Table 2). Few patients received more than one treatment (6·5%); the mean number of treatments per patient was 1·07. The mean and median total activity administered (first treatment) were 3·2 GBq (SD, 2·33) and 2·7 GBq (IQR, 1·6–4·2), respectively. Mean and median pretreatment AD to the index lesion were 435·4 Gy (SD, 360·27) and 347·6 Gy (IQR, 227·0–533·0), respectively; mean and median pretreatment AD to the total normal liver tissue were 48·1 Gy (36·89) and 39·6 Gy (IQR, 23·0–62·1), respectively; pretreatment index lesion AD was <200 Gy in 20·5%, 200 to <300 Gy in 22·2%, 300 to <400 Gy in 15·6%, and ≥400 Gy in 41·7% of patients with available dosimetry by central assessment (Table 2).

**Table 2:** SIRT treatment & dosimetry.

	N (%)
Dosimetry Software for Treatment Planning	–
Yes	942 (95·2)
No	46 (4·7)
Missing	1 (0·1)
Pretreatment Dosimetry^a^	–
SCD	251 (25·4)
MCD	736 (74·4)
Missing	2 (0·2)
TheraSphere Administration	–
Selective	530 (53·6)
Non-selective	425 (43·0)
Mixed	24 (2·4)
Missing	10 (1·0)
Repeat Treatments	–
Two	64 (6·5)
Three	4 (0·4)
Pretreatment Dosimetry^b^, ^c^, mean (SD)	–
Total perfused liver volume (cm^3^)	826·9 (633·19)
Total perfused tumor volume (cm^3^)	267·7 (397·41)
Total perfused normal liver volume (cm^3^)	555·8 (410·34)
Pretreatment Absorbed Dose to Index Lesion (Gy)^b^, ^c^	–
<200	150 (15·2)
200–<300	163 (16·5)
300–<400	114 (11·5)
≥400	306 (30·9)

Abbreviations: CT, computed tomography; MCD, multicompartment dosimetry; MRI, magnetic resonance imaging; PET, positron emission tomography; SCD, single compartment dosimetry; SPECT, single-photon emission computed tomography.

a Assessment by investigator.

b Based on first SIRT treatment.

c Assessment by central review.

Objective response rate was reported by investigators for 940 of 989 patients using either RECIST 1·1 or mRECIST, and index lesion response for 796 patients using RECIST 1·1 or mRECIST. A best overall response of complete response (CR) or partial response (PR) was achieved in 228/498 (45·8%) of patients according to RECIST 1·1 and 249/442 (56·3%) according to mRECIST (Table 3). A best index lesion response of CR/PR was achieved in 539/796 (67·7%) according to RECIST 1·1 or mRECIST. Median time-to-progression of the index lesion, according to either RECIST 1·1 or mRECIST, was 23·8 M (95% CI, 15·5 – not evaluable). Median overall time-to-progression, according to either RECIST 1·1 or mRECIST, was 9·1 M (95% CI, 7·5–10·3).

**Table 3:** Tumor response: Best overall response and index lesion response for patients with available response data.

	Best overall response^a^ N = 940		Best index lesion response N = 796
	RECIST 1·1 N = 498 n (%)	mRECIST N = 442 n (%)	RECIST 1·1/mRECIST N = 796 n (%)
Complete response	82 (16·5)	87 (19·7)	202 (25·4)
Partial response	146 (29·3)	162 (36·7)	337 (42·3)
Stable disease	107 (21·5)	74 (16·7)	160 (20·1)
Progressive disease	147 (29·5)	99 (22·4)	90 (11·3)
Other	2 (0·4)	3 (0·7)	–
Not evaluable	7 (1·4)	11 (2·5)	–
Missing/Not assessed	7 (1·4)	6 (1·4)	7 (0·9)

a Best response was determined using Investigator reported response per RECIST or mRECIST, N denominator for RECIST and mRECIST are different patients.

**Fig. 1:**
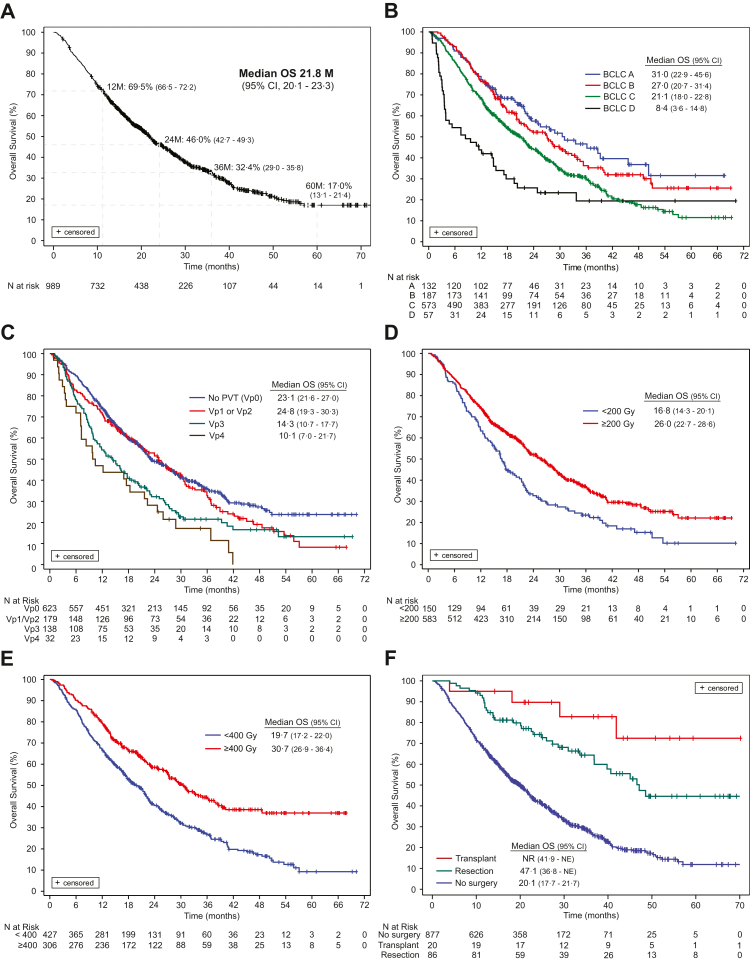
**Overall Survival (OS).** OS (A) for all 989 HCC patients; (B) across BCLC stages A, B, C, and D; (C) in patients with no PVT versus PVT (Vp1-2, Vp3, Vp4); by dose according to (D) < 200 Gy versus ≥200 Gy, and (E) < 400 Gy versus ≥400 Gy; and (F) further stratified by subsequent transplantation, resection, or no surgery. Abbreviations: M, months; NE, not evaluable; N, number; NR, not reached.

Median follow-up [95% CI] lasted 34·7 M [33·1–37·0]. In the overall cohort, median OS (mOS) was 21·8 M [20·1–23·3] (Fig. 1A). In multivariable Cox regression models, age, Child-Pugh score, ALBI score, tumor size, tumor burden, PVT, and pretreatment tumor absorbed dose to the index lesion were independently associated with survival (Table 4). By BCLC stage, mOS was 31·0 M for A, 27·0 M for B, 21·1 M for C, and 8·4 M for D (Table 4, Fig. 1B; p < 0·001). mOS was 23·1 M [21·6–27·0] for patients without PVT, and 18·3 M [15·4–22·2] for all patients with PVT (p < 0·001). Patients with Vp1/Vp2 PVT had a mOS of 24·8 M, essentially equivalent to patients without PVT (HR 0·99 [95% CI, 0·68–1·45]) (Fig. 1C; p < 0·001). In contrast, more extensive PVT (Vp3 and Vp4) was associated with a higher risk of death (HR 1·73 [95% CI, 1·13–2·67], and HR 2·82 [95% CI, 1·40–5·69], respectively) (Table 4). Supplementary Table S2 provides the mOS for patients with advanced disease (Child-Pugh B7, >B7, ECOG 1, 2).

**Table 4:** Overall survival by subgroup.

	N (%)	Overall Survival		Univariable Analysis			Multivariable Analysis		
		Median (months)	95% CI	HR	95% CI	p-value	HR	95% CI	p-value
Age^a^	–	–	–	–	–	0·002	–	–	0·001
≥18–<65	260 (26·3)	20·6	17·5–25·5	1	1	–	1	1	–
≥65–<75	401 (40·5)	26·0	22·5–29·7	0·86	0·70–1·04	–	1·03	0·73–1·46	–
≥75	328 (33·2)	17·9	16·1–21·1	1·19	0·97–1·45	–	1·77	1·23–2·56	–
BCLC Stage^b^	–	–	–	–	–	<0·001	–	–	0·220
A	132 (13·3)	31·0	22·9–45·6	1	1	–	1	1	–
B	187 (18·9)	27·0	20·7–31·4	1·17	0·85–1·59	–	0·73	0·44–1·20	–
C	573 (57·9)	21·1	18·0–22·8	1·60	1·23–2·09	–	0·52	0·28–0·98	–
D	57 (5·8)	8·4	3·6–14·8	2·79	1·89–4·11	–	0·66	0·25–1·74	–
Child-Pugh^a^	–	–	–	–	–	<0·001	–	–	0·023
A5	618 (62·5)	25·5	22·8–28·4	1	1	–	1	1	–
A6	190 (19·2)	16·0	12·6–20·0	1·67	1·37–2·02	–	1·42	1·04–1·96	–
B and C	68 (6·9)	10·4	7·2–14·8	2·02	1·51–2·70	–	1·79	1·06–3·02	–
ALBI Grade^a^	–	–	–	–	–	<0·001	–	–	0·230
1	380 (38·4)	29·6	26·7–34·9	1	1	–	1	1	–
2	440 (44·5)	18·8	16·4–20·8	1·62	1·36–1·93	–	1·20	0·90–1·59	–
3	15 (1·5)	3·0	2·0–4·7	7·12	4·12–12·29	–	2·82	0·65–12·20	–
ECOG^a^	–	–	–	–	–	0·001	–	–	0·369
0	568 (57·4)	25·2	22·4–27·9	1	1	–	1	1	–
>0	349 (35·3)	17·7	14·5–21·1	1·33	1·13–1·57	–	1·17	0·83–1·64	–
Number of Tumors^c^	–	–	–	–	–	0·105	–	–	0·678
1	485 (49·0)	22·7	20·8–26·5	1	1	–	1	1	–
> 1	484 (48·9)	20·8	17·7–23·1	1·14	0·97–1·33	–	1·16	0·57–2·35	–
Index Lesion Size^c^ (mRECIST)	–	–	–	–	–	–	–	–	–
≤5 cm	294 (31·8)	25·2	21·2–28·6	1	1	0·045	1	1	0·247
>5 cm	631 (68·2)	21·2	18·5–22·8	1·20	1·00–1·43	–	0·81	0·57–1·16	–
≤7 cm	517 (55·9)	24·8	22·1–28·0	1	1	<0·001	1	1	0·019
>7 cm	408 (44·1)	18·5	16·2–21·7	1·36	1·16–1·60	–	1·55	1·08–2·24	–
Liver Tumor Burden^c^	–	–	–	–	–	<0·001	–	–	0·003
<25%	606 (61·3)	24·8	22·1–27·4	1	1	–	1	1	–
≥25%	128 (12·9)	12·9	9·6–17·2	1·77	1·41–2·22	–	2·14	1·31–3·49	–
Liver Status^a^, ^d^	–	–	–	–	–	0·150	–	–	–
Cirrhosis/fibrosis	798 (80·7)	21·2	18·3–22·7	1	1	–	–	–	–
No cirrhosis/fibrosis	138 (14·0)	25·2	20·3–33·2	0·82	0·64–1·04	–	–	–	–
Unknown	53 (5·4)	28·4	18·3–39·2	0·81	0·57–1·16	–	–	–	–
Disease Extent^a^	–	–	–	–	–	0·001	–	–	0·537
Unilobar	763 (77·1)	22·5	21·2–25·2	1	1	–	1	1	–
Bilobar	184 (18·6)	16·4	14·0–19·6	1·38	1·14–1·67	–	1·10	0·81–1·50	–
Disease Etiology^a^, ^d^	–	–	–	–	–	0·271	–	–	–
No underlying liver disease	168 (17·0)	25·0	20·8–31·2	1	1	–	–	–	–
Alcohol	283 (28·6)	17·7	15·4–21·8	1·32	1·03–1·68	–	–	–	–
HBV	35 (3·5)	29·2	9·5–43·6	0·92	0·57–1·51	–	–	–	–
HCV	102 (10·3)	21·7	16·4–27·4	1·13	0·83–1·54	–	–	–	–
MASH	57 (5·8)	26·6	17·4–35·9	0·99	0·68–1·43	–	–	–	–
Mixed	217 (21·9)	22·7	18·3–26·9	1·12	0·87–1·46	–	–	–	–
Other	127 (12·8)	20·0	16·5–26·6	1·21	0·90–1·62	–	–	–	–
PVT^c^	–	–	–	–	–	<0·001	–	–	0·003
Vp0	623 (63·0)	23·1	21·6–27·0	1	1	–	1	1	–
Vp1 or Vp2	179 (21·5)	24·8	19·3–30·3	1·15	0·94–1·41	–	0·99	0·68–1·45	–
Vp3	138 (39·5)	14·3	10·7–17·7	1·63	1·31–2·02	–	1·73	1·13–2·67	–
Vp4	32 (9·2)	10·1	7·0–21·7	2·22	1·52–3·24	–	2·82	1·40–5·69	–
Prior Systemic Treatment^a^, ^d^	–	–	–	–	–	0·340	–	–	–
Yes	124 (12·5)	21·3	16·4–27·4	1	1	–	–	–	–
No	864 (87·4)	22·1	20·1–24·0	0·89	0·71–1·13	–	–	–	–
Prior TACE Treatment^a^	–	–	–	–	–	0·049	–	–	0·065
Yes	218 (22·0)	19·5	15·9–22·9	1	1	–	1	1	–
No	770 (77·9)	22·4	21·1–25·0	0·83	0·70–1·00	–	0·75	0·56–1·02	–
Dosimetry^a^	–	–	–	–	–	0·403	–	–	–
SCD	251 (25·4)	21·3	17·6–23·3	1	1	–	–	–	–
MCD	736 (74·4)	22·1	20·1–24·8	0·93	0·78–1·11	–	–	–	–
SIRT Administration^a^	–	–	–	–	–	0·009	–	–	0·155
Selective	530 (53·6)	23·9	21·9–27·0	1	1	–	1	1	–
Non-selective	425 (43·0)	18·8	16·1–21·7	1·28	1·09–1·50	–	1·25	0·96–1·62	–
Mixed	24 (2·4)	17·2	9·2 – NE	1·11	0·66–1·86	–	1·71	0·75–3·88	–
Pretreatment AD to Index Lesion^c^ (Gy)		–	–	0·95	0·91–0·98	0·002	1·08	1·02–1·15	0·008
<200	150 (15·2)	16·8	14·3–20·1	1	1	<0·001	1	1	0·090
200–<300	163 (16·5)	21·7	17·7–26·7	0·80	0·61–1·04	–	0·92	0·58–1·46	–
300–<400	114 (11·5)	22·0	16·0–25·5	0·89	0·67–1·18	–	1·05	0·63–1·74	–
≥400	306 (30·9)	30·7	26·9–36·4	0·52	0·41–0·66	–	0·61	0·34–1·09	–

Abbreviations: AD, absorbed dose; ALBI, albumin-bilirubin; BCLC, Barcelona Clinic Liver Cancer; CI, confidence interval; ECOG, Eastern Cooperative Oncology Group; HBV, hepatitis B; HCV, hepatitis C; HR, hazard ratio; MASH, metabolic dysfunction-associated steatohepatitis; MCD, multicompartment dosimetry; mRECIST, modified Response Evaluation Criteria in Solid Tumors; NE, not evaluable; PVT, portal vein thrombosis; SCD, single compartment dosimetry; TACE, transarterial chemoembolization.

a Assessment by investigator.

b Programmatically derived using data entered by investigator.

c Assessment by central review.

d Only subgroups with p-value <0·15 by univariable analysis were included in multivariable analysis.

Pretreatment AD to the index lesion (continuous variable) was significantly associated with OS (HR 1·08 [1·02–1·15]; p = 0·008). In prespecified dose subgroups, mOS increased across higher pretreatment AD strata; 16·8 M for <200 Gy, 21·7 M for 200 to <300 Gy, 22·0 M for 300 to <400 Gy, and 30·7 M for ≥400 Gy (p < 0·001). mOS was 16·8 M for patients with pretreatment AD to the index lesion <200 Gy and 26·0 M for those receiving ≥200 Gy (p < 0·001; Fig. 1D); mOS was 19·7 M for patients with a pretreatment AD to the index lesion <400 Gy versus 30·7 M for those receiving ≥400 Gy (p < 0·001) (Fig. 1E), confirming patients with a pretreatment tumor absorbed dose of ≥200 Gy had prolonged mOS compared with <200 Gy, and ≥400 Gy exhibiting additional benefit. When stratified by tumor dose and tumor size, mOS was numerically higher for patients with small lesions (≤5 cm) who were treated with a tumor dose ≥200 Gy (27·4 M [22·1–31·2]) compared with <200 Gy (17·8 M [16·3–32·0]; p = 0·450). In large lesions (>5 cm), mOS was significantly higher in patients treated with ≥200 Gy (25·0 M [21·7–28·3]) compared with <200 Gy (15·4 M [12·0–19·7]; p < 0·001). Similarly, mOS was 27·4 M [21·8–32·7] for patients with lesions ≤5 cm who were treated with a tumor dose ≥400 Gy versus 22·4 M [17·7–29·7] with <400 Gy (p = 0·235). In lesions >5 cm, mOS was 36·4 M [27·1 – not evaluable] versus 18·5 M [14·9–21·3] in patients treated with ≥400 Gy versus <400 Gy, respectively (p < 0·001).

Subsequent anti-cancer therapy was administered to 531 of 989 patients (53·7%) (Supplementary Table S3); 401 of 989 (40·5) had no subsequent treatment; and treatment status was unknown in 57 of 989 patients (5·7%). Per local decision, 106 (10·7%) patients were considered eligible for surgery after SIRT, of whom, 24 (18·2%), 28 (15·0%), 50 (8·7%), and 7 (12·3%) patients were BCLC A, B, C, and D, respectively; 26 (14·5%) were Vp1/Vp2 and 17 (12·3%) were Vp3. Patients who underwent subsequent surgery had markedly improved OS compared to those who did not. mOS was not reached after transplantation (n = 20) and was 47·1 M [36·8 – not evaluable] after resection (n = 86), whereas it was 20·1 M [17·7–21·7] for patients without subsequent surgery (n = 877) (Table 4, Fig. 1F; p < 0·001).

QoL was reported for 504 patients at baseline, 240 patients at 4 M post-Y90, 122 patients at 8 M, and 69 patients at 12 M. Median TTD QoL was 10·6 M [9·6–11·7], censoring at the last QoL assessment prior to subsequent treatment.

All safety events were considered treatment-emergent since all events occurred after Y90 treatment; 78 of 989 (7·9%) patients experienced 114 AEs, including 12 patients (1·2%) with grade 2 events, 45 (4·6%) with grade 3, 5 (0·5%) with grade 4, and 16 patients (1·6%) with grade 5 events. SAEs (n = 96) occurred in 74 of 989 (7·5%) patients, and 37 of 989 (3·7%) had at least one treatment-related SAE (42 events). The most frequent SAEs were ascites (13/989 [1·3%]), gastrointestinal hemorrhage (5/989 [0·5%]), hepatic encephalopathy 5/989 [0·5%]), septic shock (4/989 [0·4%]), and myocardial infarction (3/989 [0·3%]) (Table 5). Ascites was the most common treatment-related SAE (11/989 [1·1%]) (Table 5). In total, 19 patients had AEs leading to death (2 liver failure, 5 cardiac/vascular disorders, 3 infections, 3 general disorders, 2 hemorrhage, 1 ascites, 1 hepatic encephalopathy, 1 renal impairment, and 1 acute respiratory failure). Among these 19 events, 16 were categorized as grade 5, and 3 events (cardiac failure, renal impairment, and general health deterioration) were categorized as grade 3 by the investigators. Due to a low number of related liver AEs, no correlation was assessed between any factors (including pretreatment normal liver tissue absorbed dose) and occurrence of AEs. Median duration of hospitalization for the 12 patients with device-related complications was 7·5 days (IQR, 5·5–15·0). No patients were hospitalized for greater than 30 days. Investigators reported clinical and/or biological liver decompensation for 40 of 657 patients (6·1%) at 4 M, 23 of 364 (6·3%) at 8 M, and 8 of 243 (3·3%) at 12 M.

**Table 5:** Summary of SAEs reported in ≥0·2% of patients and treatment-emergent SAEs.

MedDRA SOC Preferred term	Any SAE	Treatment-related SAE^a^
	n (%)	n (%)
Hepatobiliary disorders	21 (2·1)	19 (1·9)
Ascites	13 (1·3)	11 (1·1)
Hepatic failure	2 (0·2)	2 (0·2)
Hepatic function abnormal	2 (0·2)	2 (0·2)
Gastrointestinal disorders	16 (1·6)	10 (1·0)
Gastrointestinal hemorrhage	5 (0·5)	3 (0·3)
Abdominal pain	2 (0·2)	1 (0·1)
Duodenal ulcer	2 (0·2)	2 (0·2)
Infections and Infestations	10 (1·0)	1 (0·1)
Septic shock	4 (0·4)	1 (0·1)
Pneumonia	2 (0·2)	0
General disorders and administration site conditions	9 (0·9)	3 (0·3)
Asthenia	2 (0·2)	2 (0·2)
Death	2 (0·2)	0
General physical health deterioration	2 (0·2)	0
Cardiac disorders	7 (0·7)	0
Myocardial infarction	3 (0·3)	0
Cardiac failure	2 (0·2)	0
Nervous system disorders	6 (0·6)	2 (0·2)
Hepatic encephalopathy	5 (0·5)	2 (0·2)
Respiratory, thoracic and mediastinal disorders	5 (0·5)	0
Acute pulmonary edema	2 (0·2)	0
Vascular disorders	5 (0·5)	4 (0·4)
Shock hemorrhagic	2 (0·2)	2 (0·2)
Renal and urinary disorders	3 (0·3)	0
Acute kidney injury	2 (0·2)	0

Abbreviations: MedDRA, Medical Dictionary for Regulatory Activities; SAE, serious adverse event; SOC, System Organ Class.

a Suspected to be related to procedure and/or device.

## Discussion

PROACTIF was an all-comers study for patients with locally advanced hepatic malignancies treated with Y90 glass microspheres following multidisciplinary tumor board decision. This study represents the largest prospective evaluation of HCC patients treated with SIRT, providing invaluable real-world data on current clinical practice. All centers using Y90 glass microspheres in France were invited without restriction, favoring unbiased and comprehensive patient inclusion. Most patients had intermediate stage (18·9%) or advanced (63·7%) disease, and preserved liver function. The efficacy of SIRT in clinical practice is reflected by the reported mOS of 21·8 M in the overall HCC population, with stage-specific outcomes of 31·0, 27·0, 21·0, and 8·4 M for BCLC A, B, C, and D, respectively, 24·8 M in patients with Vp1/Vp2 PVT, and more than 47·1 M in patients who became suitable for subsequent surgery.

PROACTIF results compare favorably with other SIRT real-world studies. In the US RESIN registry, mOS for patients with BCLC B, C, and D were 19·5, 13·6, and 11·5 M, respectively.^16^ In the European CIRT registry, also using resin microspheres, mOS was 20·4, 12·6, and 12·5 M for patients with BCLC B, C and D, respectively.^17^^,^^18^

The PROACTIF protocol recommended personalized dosimetry. The recommended dose threshold was ≥400 Gy to the perfused volume with SCD for radiation segmentectomy, and ≥300 Gy to the tumor with MCD, while respecting dose to normal tissue. PROACTIF investigators largely implemented these recommendations, highlighting the theranostic ability of Y90 glass microspheres. Mean AD to the index lesion was 435·4 Gy, and increasing dose was associated with improved survival. mOS rose from 16·8 M for patients with a pretreatment AD to the index lesion <200 Gy to 26·0 M for those receiving ≥200 Gy. These findings are consistent with DOSISPHERE-01, which demonstrated a survival benefit for patients with a dose >205 Gy.^8^ Interestingly, patients treated with ≥400 Gy to the index lesion had substantially longer survival (median, 30·7 M) than those treated with <400 Gy (19·7 M). Further, the survival benefit was superior in patients with an index lesion >5 cm and index lesion dose ≥400 Gy (36·4 M) versus <400 Gy (18·5 M; p < 0·001). As the tumor heterogeneity increases with tumor size, the benefit of further increasing the median tumor dose for large lesions is the consequence of a sufficient dose delivered to the less vascularized tumor area, leading to an overall better tumor control. This suggests that a median tumor AD ≥400 Gy might be considered as a threshold for lesions larger than 5 cm. These data are consistent with a recent study evaluating Y90 glass microspheres in large tumors (≥5 cm) which identified a threshold of 586 Gy that more than doubled OS,^6^ further supporting an increase to the mean AD for large tumors to overcome the heterogeneity. Therefore, targeting the highest possible AD to the target lesion while respecting the dose to normal liver tissue and maintaining hepatic reserve should remain the goal for treatment planning.

mOS observed in BCLC A is lower compared with results typically reported with ablative strategies such as radiation segmentectomy.^1^^,^^19^ This can be explained mainly by the large tumor size in PROACTIF where 68·9% of BCLC A patients had solitary tumors >5 cm versus only 5·6% in LEGACY.^1^ Median survival for patients with BCLC B (18·9%) and C (57·9%) disease – the majority in PROACTIF – was comparable to contemporary studies with glass microspheres,^20^^,^^21^ including DOSISPHERE-01, where optimized tumor dosing yielded a mOS of 26·7 M.^8^

RCTs evaluating systemic treatments have included a similar proportion of BCLC B and C patients who were unsuitable for resection or locoregional treatment. The mOS reported in PROACTIF were in line with survival outcomes reported in pivotal systemic RCTs.^22^, ^23^, ^24^, ^25^, ^26^ However, cross-trial comparisons must be interpreted with caution due to differences in study design and patient baseline characteristics. For example, few patients in PROACTIF had extrahepatic disease compared with 30% in systemic trial populations. In addition, survival in real-world systemic cohorts is typically lower than in RCTs owing to broader eligibility and more comorbidities.^23^^,^^27^, ^28^, ^29^, ^30^

PVT presence and extent are consistently associated with poor prognosis in HCC.^31^^,^^32^ When and how to integrate locoregional treatment in patients with PVT remains debated.^3^^,^^33^, ^34^, ^35^ In PROACTIF, mOS was 18·3 months in a large population of 349 (35·3%) patients with PVT (including Vp4). Patients with segmental/sectorial PVT (Vp1/Vp2) had a mOS comparable to those without PVT, suggesting that personalized dosimetry-based treatment can at least partially mitigate the poor prognosis of Vp1-Vp2 PVT, although OS beyond the median still favored patients without PVT. Extended OS for well-selected PVT patients has previously been reported in a single-center retrospective study including 120 PVT patients, where mOS ranged from 7·8–32·2 M, depending on the extent of PVT and other baseline characteristics (baseline bilirubin level and tumor burden).^36^ For the subgroup of Vp1 patients (also with bilirubin levels ≤1·2 mg/dL and tumoral burden ≤50%) median OS was 32·2 M, but the population of this retrospective study was slightly different compared with PROACTIF (a younger population and mostly ECOG 0 [95·8%]).

Despite the limitations of cross-study comparison, these outcomes compare equally or even better than those obtained with systemic therapies. In IMbrave150, 177 patients in the investigational arm had PVT (42% Vp1–2; 31% Vp3; 27% Vp4)^37^; mOS for all PVT patients without Vp4 was 21·1 M, and 7·6 M for patients with Vp4; mOS was not reported for Vp1/Vp2, but in both cases, the risk of death was doubled compared with patients without PVT (HR 2·06 and 2·08, respectively).^37^ Moreover, OS by detailed PVT extent is variably reported or not reported in other systemic RCTs, and many exclude Vp4.^23^^,^^25^ This is clinically relevant, as PVT extension is a key determinant of treatment selection, including for SIRT. In CheckMate 9DW, mOS was 22·9 M among 77 patients with PVT, without stratification between Vp1-Vp3, and no information regarding the proportion of non-viral liver disease in these patients.^26^

Fifty-seven BCLC D patients were treated in this study; mOS was 8·4 M. These patients are normally excluded in SIRT RCTs and other trials with systemic therapies, however, PROACTIF was a real-world study, and there was no restriction on treating BCLC D patients. mOS of patients with baseline reported ALBI grade 3 (3·0 M) and Child-Pugh score > B7 (3·6 M) were dramatically reduced and should not be treated. For patients with ECOG 3 or 4, as for patients with severe ascites, no specific recommendation can be made based on our results (due to a low number of patients) but the general recognized recommendation is that those patients should not be treated with SIRT. In addition, the survival curve for BCLC D patients dropped from treatment to Month 4 but then remained parallel with BCLC B and C patients, suggesting that select BCLC D patients may benefit from treatment but not all.

Several features of PROACTIF help to explain the meaningful mOS, particularly in advanced disease. Most patients considered for treatment had a favorable prognosis: 77% had unilobar disease, 49% had a solitary tumor, and 82% were Child-Pugh A; patients with these characteristics are the most suitable for SIRT as a high AD can be delivered to a limited liver volume, thus sparing more normal liver tissue and preserving liver function. Other features of PROACTIF may have negatively impacted survival outcomes. First, the cohort was older than typical RCT populations (median age of 71·0 y versus 53–65 in most systemic trials). Second, patients had substantial comorbidities, including a high prevalence of alcohol use. Third, 58% of patients had non-viral etiology cirrhosis, while it generally represents 15–41% of patients enrolled in systemic RCTs (up to 95% in Chinese studies) where patients with underlying liver disease of viral etiology have a longer median OS relative to alcohol-induced cirrhosis.^24^, ^25^, ^26^ Fourth, PROACTIF included patients with large tumors (median index lesion 6·8 cm), a well-established adverse prognostic factor across treatments, including systemic therapy.

Another salient finding is the conversion to surgery among initially unresectable patients, including some with BCLC C and D. Prior studies have consistently reported downstaging/bridging to transplantation or resection in ∼18–35% of patients after SIRT^1^^,^^6^^,^^8^^,^^20^; the rate in PROACTIF was 10·7%. This lower proportion likely reflects the pragmatic, unselected, nationwide design (34 centers, including many with low SIRT volumes and without large hepatobiliary units) together with an older case-mix, substantial tumor burden (median index lesion, 6·8 cm), and frequent PVT. Despite this, patients who proceeded to surgery achieved the best outcomes: mOS was not reached after transplantation, 47·1 M after resection, and 20·1 M in patients without subsequent surgery. Notably, similar proportions of patients with Vp1-Vp3 (no Vp4) underwent surgery. Such conversion rates and survival gains are rarely observed in systemic therapy trials, where subsequent surgery is not frequent. These data emphasize the importance of repeated multidisciplinary assessment to identify delayed candidates to curative-intent surgery.

Regarding QoL in PROACTIF, the analysis was impacted by the low rate of completion due to the voluntary nature of the questionnaire. Median TTD was 10·6 M, and despite differences in the definition/calculation, seems in line with the results obtained with immunotherapy in IMbrave150 (11·2 M).

For safety, only related grade ≥3 AEs and all SAEs were required to be reported, therefore, unrelated non-serious AEs were not collected. Consequently, the observed rate of related grade ≥3 AEs (7·9%) was lower than what is usually reported,^8^^,^^38^ and lower than the CIRT (10·3%) and RESIN registries (16·9%). Importantly, despite high tumor doses, longitudinal liver function data indicated that SIRT generally preserved eligibility for subsequent locoregional or systemic therapies, as 53·7% received additional anti-cancer treatment, confirming the safety of SIRT.

PROACTIF has limitations inherent to a phase IV post-approval design, including some missing data. Collection of non-standard data (QoL, non-serious AEs) and documentation was limited. AEs were likely underreported; however, the high proportion of patients receiving subsequent therapy after SIRT supports a favorable safety profile. Although post-approval studies provide a lower level of evidence than RCTs, data capture and monitoring in PROACTIF were extensive, with long follow-up, approaching RCT standards.

Overall, PROACTIF was a large all-comers study in a representative, real-world population, demonstrating clinically meaningful effectiveness and safety of Y90 glass microspheres across HCC stages. Accordingly, these data allowed the maintenance of reimbursement in France and should further help with reimbursement in other European Union countries, as it demonstrated that the treatment is well integrated in the armamentarium of HCC treatments, and appropriately used in selected patients for specific clinical purposes. The best outcomes were observed in patients achieving index tumor absorbed doses ≥400 Gy, and in those who subsequently underwent curative surgery after downstaging with SIRT, including patients with PVT. These findings reinforce the central importance of rigorous patient selection and treatment technique, using personalized dosimetry, in optimizing outcomes, and also demonstrate that SIRT with accurate personalized dosimetry is feasible at a large scale. This high-quality real-world data should inform the optimal use of Y90 and help refine future HCC treatment recommendations.

## Contributors

BG, EB, KF, BP, EG were involved in the conception and design. BG, CB, EVib, GT, DMG, JE, YT, ED, JFB, JC, HR, AB, GS, CSe, SR, AR, CSo, PC, VH, IBR, ADo, LT, SM, ADi, EVic, and EG provided data from patients included in the study. BG, EB, KF, BP, PS, EG were involved in analysis and interpretation. BG, EB, KF, BP, PS, EG contributed to manuscript development. All authors reviewed the manuscript. BG, EB, KF, BP, PS, EG had access to raw data. The corresponding author (EG) had final responsibility for the decision to submit for publication. All authors had access to the data and accept responsibility to submit for publication.

## Data sharing statement

Data from this study will be shared upon reasonable request. Individual participant data will not be shared.

## Declaration of interests

BG reports grants or contracts from Boston Scientific Corporation and Roche/Genentech; consulting fees from Boston Scientific Corporation, Canon Medical Systems, Roche/Genentech, and AstraZeneca; honoraria from Boston Scientific Corporation, Canon Medical Systems, Roche/Genentech, AstraZeneca, Ipsen, Terumo, and Guerbet; travel, accommodations, and expenses from Boston Scientific Corporation and Canon Medical Systems; Data Safety Monitoring Board or Advisory Board participant for Terumo; CB reports consulting fees and honoraria from Boston Scientific Corporation. EVib reports grants or contracts from Relyens; patronage from Johnson & Johnson, Fuji Film, and Vermon through the AP-HP Fondation; Board Membership for Boston Scientific Corporation. JE reports grants or contracts from Bristol Myers Squibb, BeiGene, Boston Scientific Corporation, Exeliom Biosciences, SUMMIT, and AstraZeneca; consulting fees from Merck Sharp & Dohme, Eisai, Bristol Myers Squibb, AstraZeneca, Bayer, Roche/Genentech, Ipsen, Basilea, Merck Serono, Incyte, Servier, BeiGene, Taiho, Boston Scientific Corporation, Guerbet, Jazz, Captor Therapeutics; advisory board participant for Captor Therapeutics. YT reports honoraria from Roche/Genentech, AstraZeneca, Bristol Myers Squibb; and travel, accommodations, and expenses from Roche/Genentech and AstraZeneca. ED reports holding a treasurer role for Association Biophysique - Bicêtre. JFB reports consulting fees from Merck Sharp & Dohme, Roche/Genentech, AstraZeneca, and Bristol Myers Squibb; honoraria from Roche/Genentech, AstraZeneca, and Sirtex Medical; travel, accommodations, and expenses from Roche/Genentech, AstraZeneca, and Merck Sharp & Dohme; advisory board participation for Roche/Genentech, AstraZeneca, Merck Sharp & Dohme, and Bristol Myers Squibb. HR reports consulting fees from Boston Scientific Corporation, and Sirtex Medical. VH reports renumerations from Advanced Accelerator Applications; travel, accommodations, and expenses from Advanced Accelerator Applications, Boston Scientific Corporation, and Sirtex Medical; member of the Targeted Radionuclide Therapy Working Group of the French Society of Nuclear Medicine. ADo reports honoraria from Merit Medical, Varian Medical, and Boston Scientific Corporation. LT reports consulting fees from Quantum Surgical, GE Healthcare; honoraria from Boston Scientific Corporation, Terumo, and Guerbet; patents for Pickering emulsions, and for a radiological clip; advisory board participant for Terumo. TDB reports consulting fees from Boston Scientific Corporation, General Electric, Guerbet, Terumo, and Quantum; honoraria from Guerbet and Terumo; patents for Pickering emulsions; advisory board participant for Terumo. SM reports travel, accommodations, and expenses from AstraZeneca and Merck Sharp & Dohme; advisory board participant for Boston Scientific Corporation. ADi reports grants from Guerbet, Siemens, Normandy Region, Normandy League Against Cancer; honoraria from Sirtex Medical; patent with SimpleDose software; advisory board participant for Boston Scientific Corporation; and a leadership role in the French Society of Medical Physics (SFPM). KF reports being a non-commercial employee of Boston Scientific Corporation. EB reports being a non-commercial employee of Boston Scientific Corporation. BP reports being a non-commercial employee of Boston Scientific Corporation. EVic reports consulting fees and Scientific Committee participant with Boston Scientific Corporation. EG reports consulting fees and honoraria from Boston Scientific Corporation. All other authors (GT, DMG, JC, AB, CSe, AR, CSo, IBR, GS, SR, and PC) declare no competing interests.

## Appendix A Supplementary data

Supplementary Figure and Tables

PROACTIF Protocol

## References

[1] Salem R., Johnson G.E., Kim E. (2021). Yttrium-90 radioembolization for the treatment of solitary, unresectable HCC: the LEGACY Study. Hepatology.

[2] Vogel A., Martinelli E., Vogel A. (2021). Updated treatment recommendations for hepatocellular carcinoma (HCC) from the ESMO Clinical Practice Guidelines. Ann Oncol.

[3] (2025). EASL Clinical Practice Guidelines on the management of hepatocellular carcinoma. J Hepatol.

[4] Reig M., Forner A., Rimola J. (2022). BCLC strategy for prognosis prediction and treatment recommendation: the 2022 update. J Hepatol.

[5] Reig M., Sanduzzi-Zamparelli M., Forner A. (2025). BCLC strategy for prognosis prediction and treatment recommendations: the 2025 update. J Hepatol.

[6] Choi J.W., Suh M., Choi Y., Lee M., Paeng J.C., Kim H.C. (2025). Yttrium-90 glass microsphere radioembolization as frontline treatment for hepatocellular carcinoma with localized portal vein invasion. Eur Radiol.

[7] Montazeri S.A., De la Garza-Ramos C., Silver C. (2025). Achieving complete pathologic necrosis in hepatocellular carcinoma treated with radiation segmentectomy before liver transplantation: a comprehensive glass microsphere analysis. Eur J Nucl Med Mol Imaging.

[8] Garin E., Tselikas L., Guiu B. (2021). Personalised versus standard dosimetry approach of selective internal radiation therapy in patients with locally advanced hepatocellular carcinoma (DOSISPHERE-01): a randomised, multicentre, open-label phase 2 trial. Lancet Gastroenterol Hepatol.

[9] De la Garza-Ramos C., Overfield C.J., Montazeri S.A. (2021). Biochemical safety of ablative Yttrium-90 radioembolization for hepatocellular carcinoma as a function of percent liver treated. J Hepatocell Carcinoma.

[10] Weber M., Lam M., Chiesa C. (2022). EANM procedure guideline for the treatment of liver cancer and liver metastases with intra-arterial radioactive compounds. Eur J Nucl Med Mol Imaging.

[11] Garin E., Pinaquy J.B., Bailly C. (2022). Evaluating the effectiveness of Yttrium-90 glass microspheres in the treatment of hepatocellular carcinoma, intrahepatic cholangiocarcinoma, and metastatic colorectal cancer in practice: protocol for the prospective PROACTIF phase IV registry study in France. Cardiovasc Interv Radiol.

[12] Salem R., Padia S.A., Lam M. (2019). Clinical and dosimetric considerations for Y90: recommendations from an international multidisciplinary working group. Eur J Nucl Med Mol Imaging.

[13] Brookmeyer R., Crowley J.J. (1982). A confidence interval for the median survival time. Biometrics.

[14] Kalbfleisch J., Prentice R. (2011).

[15] Menard S. (2002).

[16] Frantz S., Matsuoka L., Vaheesan K. (2021). Multicenter evaluation of survival and toxicities of hepatocellular carcinoma following radioembolization: analysis of the RESiN registry. J Vasc Interv Radiol.

[17] Helmberger T., Golfieri R., Pech M. (2021). Clinical application of trans-arterial radioembolization in hepatic malignancies in Europe: first results from the prospective Multicentre observational Study CIRSE registry for SIR-Spheres therapy (CIRT). Cardiovasc Interv Radiol.

[18] Kolligs F., Arnold D., Golfieri R. (2023). Factors impacting survival after transarterial radioembolization in patients with hepatocellular carcinoma: results from the prospective CIRT study. JHEP Rep.

[19] Kim E., Sher A., Abboud G. (2022). Radiation segmentectomy for curative intent of unresectable very early to early stage hepatocellular carcinoma (RASER): a single-centre, single-arm study. Lancet Gastroenterol Hepatol.

[20] Garin E., Tselikas L., Guiu B. (2024). Long-term overall survival after selective internal radiation therapy for locally advanced hepatocellular carcinomas: updated analysis of DOSISPHERE-01 trial. J Nucl Med.

[21] Salem R., Gabr A., Riaz A. (2018). Institutional decision to adopt Y90 as primary treatment for hepatocellular carcinoma informed by a 1,000-patient 15-year experience. Hepatology.

[22] Ren Z., Xu J., Bai Y. (2021). Sintilimab plus a bevacizumab biosimilar (IBI305) versus sorafenib in unresectable hepatocellular carcinoma (ORIENT-32): a randomised, open-label, phase 2-3 study. Lancet Oncol.

[23] Abou-Alfa G.K., Lau G., Kudo M. (2022). Tremelimumab plus durvalumab in unresectable hepatocellular carcinoma. NEJM Evid.

[24] Qin S., Chan S.L., Gu S. (2023). Camrelizumab plus rivoceranib versus sorafenib as first-line therapy for unresectable hepatocellular carcinoma (CARES-310): a randomised, open-label, international phase 3 study. Lancet.

[25] Finn R.S., Qin S., Ikeda M. (2020). Atezolizumab plus bevacizumab in unresectable hepatocellular carcinoma. N Engl J Med.

[26] Yau T., Galle P.R., Decaens T. (2025). Nivolumab plus ipilimumab versus lenvatinib or sorafenib as first-line treatment for unresectable hepatocellular carcinoma (CheckMate 9DW): an open-label, randomised, phase 3 trial. Lancet.

[27] Rimini M., Persano M., Tada T. (2023). Survival outcomes from atezolizumab plus bevacizumab versus Lenvatinib in Child Pugh B unresectable hepatocellular carcinoma patients. J Cancer Res Clin Oncol.

[28] Lo Prinzi F., Rossari F., Silletta M. (2025). Comparative effectiveness of atezolizumab plus bevacizumab versus tremelimumab plus durvalumab in patients with hepatocellular carcinoma (HCC) in a real-world setting. Target Oncol.

[29] Allaire M., Thiam E.M., Amaddeo G. (2025). Real-world outcomes of atezolizumab–bevacizumab in hepatocellular carcinoma: the prospective French CHIEF cohort. Liver Int.

[30] Cheng A.L., Qin S., Ikeda M. (2022). Updated efficacy and safety data from IMbrave150: atezolizumab plus bevacizumab vs. sorafenib for unresectable hepatocellular carcinoma. J Hepatol.

[31] Abdelhamed W., Shousha H., El-Kassas M. (2024). Portal vein tumor thrombosis in hepatocellular carcinoma patients: is it the end?. Liver Res.

[32] Qadan M., Kothary N., Sangro B., Palta M. (2020). The treatment of hepatocellular carcinoma with portal vein tumor thrombosis. Am Soc Clin Oncol Educ Book.

[33] Korean Liver Cancer Association, National Cancer Center Korea (2022). 2022 KLCA-NCC Korea practice guidelines for the management of hepatocellular carcinoma. Clin Mol Hepatol.

[34] Liu X., Xia F., Chen Y. (2024). Chinese expert consensus on refined diagnosis, treatment, and management of advanced primary liver cancer (2023 edition). Liver Res.

[35] Blanc J.F., Debaillon-Vesque A., Roth G. (2021). Hepatocellular carcinoma: French Intergroup Clinical Practice Guidelines for diagnosis, treatment and follow-up (SNFGE, FFCD, GERCOR, UNICANCER, SFCD, SFED, SFRO, AFEF, SIAD, SFR/FRI). Clin Res Hepatol Gastroenterol.

[36] Spreafico C., Sposito C., Vaiani M. (2018). Development of a prognostic score to predict response to Yttrium-90 radioembolization for hepatocellular carcinoma with portal vein invasion. J Hepatol.

[37] Finn R.S., Galle P.R., Ducreux M. (2024). Efficacy and safety of atezolizumab plus Bevacizumab versus Sorafenib in hepatocellular carcinoma with main trunk and/or contralateral portal vein invasion in IMbrave150. Liver Cancer.

[38] Lam M., Garin E., Maccauro M. (2022). A global evaluation of advanced dosimetry in transarterial radioembolization of hepatocellular carcinoma with Yttrium-90: the TARGET study. Eur J Nucl Med Mol Imaging.

