# Supplementary material for: Effectiveness and safety of selective internal radiation therapy using yttrium-90 glass microspheres for hepatocellular carcinoma: real-world results from the multi-center prospective PROACTIF cohort of 989 patients
Source: eClinicalMedicine. 2026 Apr 17;95:103884. doi: 10.1016/j.eclinm.2026.103884 (PMC13098352; doi:10.1016/j.eclinm.2026.103884)
Supplement: Supplementary Figure and Tables [file mmc1.docx]

**Supplementary Material**

**Title**: Effectiveness and Safety of Selective Internal Radiation Therapy using Yttrium-90 Glass Microspheres for Hepatocellular Carcinoma: Real-world Results from the Multi-center Prospective PROACTIF Cohort of 989 Patients

**Authors**: Boris Guiu, MD^1^; Clément Bailly, MD^2^; Eric Vibert, MD^3^; Ghoufrane Tlili, MD^4^; Denis Mariano-Goulart, MD^1,^, Julien Edeline, MD^5^; Yann Touchefeu, MD^2^; Emmanuel Durand, MD^6^; Jean Frédéric Blanc, MD^4^; Julia Chalaye, MD^7^; Hélène Regnault, MD^7^; Antoine Bouvier, MD^8^; Geraldine Sergent, MD^9^; Christian Sengel, MD^10^; Stéphane Renaud, MD^11^; Agnès Rode, MD^12^; Claude Somma, MD^13^; Patrick Chevallier, MD^14^; Vincent Habouzit, MD^15^; Isabelle Brenot-Rossi, MD^16^ ; Anthony Dohan, MD^17^; Lambros Tselikas, MD^18^; Thierry DeBaère, MD^18^; Sylvain Manfredi, MD^19^; Arnaud Dieudonné, PhD^20^; Kirk Fowers, PhD^21^; Eveline Boucher, MD^21^; Binal Patel, MPH^21^; Eric Vicaut, MD^22^; Etienne Garin, MD^5^*; and the PROACTIF Investigators Group**

**Affiliations:** ^1^Hôpital Saint-Eloi, Montpellier, France; ^2^Centre Hospitalier Universitaire de Nantes, Nantes, France; ^3^Assistance Publique-Hôpitaux de Paris, Hôpital Paul Brousse, Villejuif, France; ^4^Centre Hospitalier Universitaire de Bordeaux, Hôpital du Haut-Lévêque, Bordeaux, France; ^5^Centre Eugène Marquis, Rennes, France; ^6^Assistance Publique-Hôpitaux de Paris, Hôpital Bicêtre, Le Kremlin-Bicêtre, France; ^7^Assistance Publique-Hôpitaux de Paris, Hôpital Henri-Mondor, Créteil, France; ^8^Centre Hospitalier Universitaire de Angers, Angers, France; ^9^Centre Hospitalier Universitaire de Lille, Hôpital Claude Huriez, Lille, France; ^10^Centre Hospitalier Universitaire de Michallon, Grenoble, France; ^11^Centre Hospitalier de Perpignan, Perpignan, France; ^12^Hospices Civils de Lyon, Hôpital de la Croix Rousse, Lyon, France; ^13^Centre Hospitalier Universitaire de la Timone, Hôpital Adultes, Marseille, France; ^14^Centre Hospitalier Universitaire de Nice, Nice, France; ^15^Centre Hospitalier Universitaire de Saint-Étienne, Hôpital Nord, Saint-Étienne, France; ^16^CLCC Institut Paoli-Calmettes, Marseille, France; ^17^Assistance Publique-Hôpitaux de Paris, Hôpital Cochin, Paris, France; ^18^Institut Gustave Roussy, Villejuif, France; ^19^Centre Hospitalier Universitaire de Dijon, Université Bourgogne Europe, Dijon, France; ^20^Centre Henri Becquerel, Rouen, France ; ^21^ Boston Scientific Corporation, Malborough, MA, USA; ^22^Assistance Publique-Hôpitaux de Paris, Hôpital Lariboisière – Fernand-Widal, Paris, France

**All members of the PROACTIF Registry Group are listed in Supplementary Material

**Table of Contents**

[**Tables** 2](#_Toc225159356)

[**Supplementary Table 1. PROACTIF Investigator Group** 2](#_Toc225159357)

[**Supplementary Table 2. Baseline Characteristics** 4](#_Toc225159358)

[**Supplementary Table 3. Subsequent Anti-Cancer Treatment** 4](#_Toc225159359)

[**Figures** 4](#_Toc225159360)

[**Supplementary Figure 1. Study Consort** 4](#_Toc225159361)

[**Supplementary Methods** 4](#_Toc225159362)

# **Tables**

## **Supplementary Table 1. PROACTIF Investigator Group**

| Site | Investigators |
| --- | --- |
| Assistance Publique-Hôpitaux de Paris, Hôpital Bicêtre, Le Kremlin-Bicêtre, France | Emmanuel Durand, MD; Olivier Meyrignac, MD, PhD; Clara Prud’homme, MD; Gilles Grimon, MD |
| Assistance Publique-Hôpitaux de Paris, Hôpital Cochin, Paris, France | Anthony Dohan, MD, PhD; Maxime Barat, MD; Raphael Dautry, MD |
| Assistance Publique-Hôpitaux de Paris, Hôpital Henri-Mondor, Créteil, France | Julia Chalaye, MD; Hélène Regnault^1^, MD; Hicham Kobeiter, MD; Haytham Derbel, MD |
| Assistance Publique-Hôpitaux de Paris, Hôpital Lariboisière – Fernand-Widal, Paris, France | Eric Vicaut, MD, PhD |
| Assistance Publique-Hôpitaux de Paris, Hôpital Paul Brousse, Villejuif, France | Eric Vibert^1^, MD, PhD; Olivier Rosmorduc, MD, PhD |
| Assistance Publique-Hôpitaux de Paris, Hôpital Saint-Louis, Paris, France | Anton Pachev, MD; Thomas Aparicio, MD, PhD; Laetitia Vercellino, MD, PhD |
| Centre Eugène Marquis, Rennes, France | Etienne Garin^1^, MD, PhD; Julien Edeline, MD, PhD; Yann Rolland, MD; Julien Farce, MD; Samuel Lesourd, MD; Xavier Palard-Novello, MD, PhD |
| Centre George-François Leclerc, Dijon, France | Inna Dygai-Cochet, MD; François Godard^2^, MD; Romain Popoff, PhD^2^; Julie Pellegrinelli, MD |
| Centre Henri Becquerel, Rouen, France | Arnaud Dieudonné^1^, PhD; Stéphanie Becker, MD |
| Centre Hospitalier de Perpignan, Perpignan, France | Stéphane Renaud, MD; Aymeric Guibal, MD; Mohamed Abdel-Rehim, MD; Faiza Khemissa Akouz, MD |
| Centre Hospitalier Lyon Sud, Lyon, France | Sibel Isal, MD; Charles Mastier, MD; Arnaud Muller, MD; Guillaume Guthier, MD |
| Centre Hospitalier Régional Universitaire, Besançon, France | Paul Calame, MD; Hatem Boulahdour, MD, PhD; Vincent Di Martino, MD, PhD |
| CHRU Brabois Adultes, Nancy, France | Elodie Chevalier, MD; Valérie Laurent, MD; Xavier Orry, MD |
| CHU de Amiens, Amiens, France | Thierry Yzet, MD; Eric Nguyen-Khac, MD, PhD; Vandici Ovidiu-Florian, MD; Chalabia Fergani, MD |
| CHU de Angers, Angers, France | Antoine Bouvier, MD; Pacome Fosse, MD; Frederic Oberti, MD, PhD |
| CHU de Bordeaux, Hôpital du Haut- Lévêque, Bordeaux, France | Ghoufrane Tlili, MD; Jean Frédéric Blanc, MD, PhD; Panteleimon Papadopoulos, MD |
| CHU de Brest, Brest, France | Ronan Abral, MD, PhD; Antoine Boizet, MD; Jean Romain Risson, MD |
| CHU de Dijon, Université Bourgogne Europe, Dijon, France | Sylvain Manfredi^1^, MD, PhD; Francois Ghiringhelli, MD; Romaric Loffroy, MD |
| CHU de Hautepierre, Strasbourg, France | Michel Greget, MD; Fabrice Hubele, MD, PhD |
| CHU de la Timone, Hôpital Adultes, Marseille, France | Claude Somma, MD, PhD; Olivier Durieux, MD |
| CHU de Lille, Hôpital Claude Huriez , Lille, France | Géraldine Sergent, MD; Clio Baillet, MD; Massih Ningarhari, PhD |
| CHU de Michallon, Grenoble, France | Christian Sengel, MD; Julie Roux, MD; Julien Ghelfi, MD; Thomas Decaens, MD, PhD |
| CHU de Nantes, Nantes, France | Clément Bailly^2^, MD, PhD; Yann Touchefeu, MD, PhD; Frederic Douane, MD; Matthieu Barbaud^2^, MD |
| CHU de Nice, Nice, France | Patrick Chevallier, MD, PhD; Mohamed El Zibawi, MD; Philippe Viaud, MD; Micheline Razzouk, MD |
| CHU de Nimes, Hôpital Carremeau, Nimes, France | Jean Goupil, MD; Melanie Sainmont, MD |
| CHU de Purpan, Toulouse, France | Jean-Marie Peron, MD, PhD; Philippe Otal, MD; Fatima-Zohra Mokrane, MD, PhD |
| CHU de Rouen, Rouen, France | David Sefrioui, MD, Frederic Di Fiore, MD, PhD |
| CHU de Saint-Étienne, Hôpital Nord, Saint-Étienne, France | Vincent Habouzit, MD, MSc; Remi Grange, MD; Jean Marc Phelip, MD, PhD |
| CHU La Miletrie, Poitiers, France | Jean-Pierre Tasu, MD; David Tougeron, MD, PhD; Rémy Perdrisot MD, PhD |
| CLCC Institut Paoli Calmette, Marseille, France | Isabelle Brenot-Rossi, MD; Gilles Piana, MD; Brice Chanez, MD, PhD; Daniel Ouk, MD; Clément Mennetrey, MD |
| CRLCC Centre Léon Bérard, Lyon, France | Franck Grillet, MD; Amine Bouhamama, MD; Sandrine Parisse-Di Martino, MD |
| Hôpital Beaujon, Clichy, France | Marco Dioguardi Burgio, MD; Maxime Ronot, MD; Rachida Lebtahi, MD, PhD; Mohammed Bouattour, MD, PhD |
| Hôpital Edouard Herriot, Lyon, France | Laurent Milot, MD, PhD; Jérôme Dumortier, MD, PhD |
| Hôpital Saint-Eloi, Montpellier, France | Boris Guiu^1^, MD, PhD; Denis Mariano-Goulart, MD, PhD; Eric Assenat, MD, PhD; Carole Allimant^2^, MD; Christine Latry Kuhn, MD; Marjolaine Fourcade, PhD |
| Hospices Civils de Lyon, Hôpital de la Croix-Rousse, Lyon, France | Agnès Rode, MD; Philippe Merle, MD, PhD |
| Institut Bergonié, Bordeaux, France | Jean Palussière, MD; Vincent Prega-Renaud; MD |
| Institut Gustave Roussy, Villejuif, France | Thierry De Baère, MD, PhD; Lambros Tselikas, MD, PhD |
| Nouvel Hôpital Civil, Strasbourg, France | Iulian Enescu, MD |

^1^PROACTIF Steering Committee Member

^2^Central dosimetry reader

## **Supplementary Table 2. Baseline Characteristics**

|  |  | Overall Survival | |
| --- | --- | --- | --- |
|  | **N** | **Median (months)** | **95% CI** |
| Child-Pugh^1^ | ·· | ·· | ·· |
| B and C | 68 (6·9) | 10·4 | 7·2 – 14·8 |
| B7 | 47 (4·8) | 12·1 | 9·0 – 17·7 |
| >B7 | 21 (2·1) | 3·6 | 2·3 – 10·9 |
| ECOG^1,2^ |  |  |  |
| 1 | 329 | 18·0 | 15·4 – 21·3 |
| 2 | 15 | 18·5 | 2·1 – NE |

Abbreviations: CI, confidence interval; ECOG, Eastern Cooperative Oncology Group; N, number of patients; NE, not evaluable

^1^Assessment by investigator

^2^Only 3 patients had ECOG 3 and 2 patients had ECOG 4, so they were excluded from separate survival analyses, and have only been included in the main analysis evaluating 0 versus > 0.

## **Supplementary Table 3. Subsequent Anti-Cancer Treatment**

| Subsequent Anti-cancer Treatment, n(%) | ·· |
| --- | --- |
| Yes^1^ | 531 (53·7) |
| Subsequent Locoregional Treatment^2^ | 168 (31·6) |
| Subsequent Systemic Treatment^2^ | 295 (55·6) |
| Subsequent Liver Surgery^2^ | 106 (20.0) |
| Missing^2^ | 2 (0·4) |

^1^ Patients may have had more than one

**^2^** Percentages are out of the 531 with subsequent treatment

# **Figures**

## **Supplementary Figure 1. Study Consort**

**
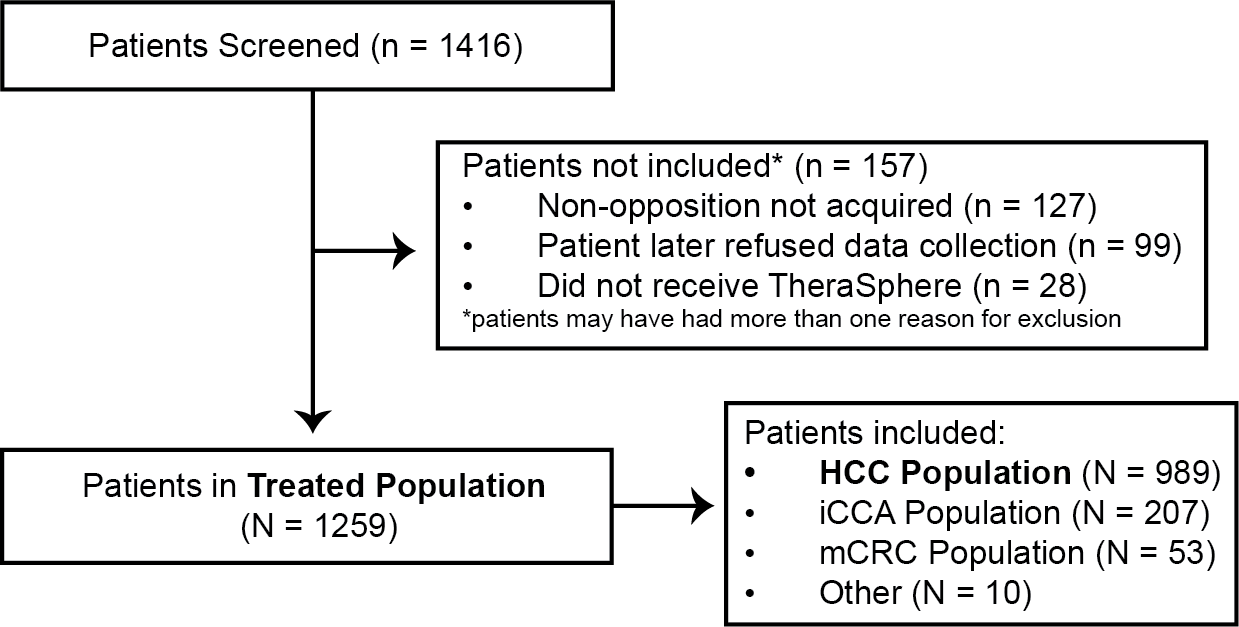
**

# **Supplementary Methods**

*Handling of Missing Data*

Partial dates and missing FACT-Hep total scores had imputation rules described below. For central dosimetry assessments, if the total perfused tumor is missing and there was only one lesion, then the volume and absorbed dose from index lesion were imputed for total perfused tumor.

*Imputation rules*

Adverse event (AE) date imputation

The following algorithm should be used to impute AE start dates for which only partial information is known:

- Missing day and month
  - If the year is the same as the year of first TheraSphere treatment, then the day and month of the start date of treatment will be assigned to the missing fields
- If the year is prior to the year of first TheraSphere treatment, then December 31 will be assigned to missing fields
- If the year is after the year of first TheraSphere treatment, then January 1 will be assigned to the missing fields
- Missing month only
  - Treat day as missing and replace both month and day accordingly to the procedure above
- Missing day only
  - If the month and year are the same as the month and year of first TheraSphere treatment, then the start date of treatment will be assigned to the missing day
- If the month and year are before the month and year of first TheraSphere treatment, then the last day of the month will be assigned to the missing day
- If the month and year are after the month and year of the first TheraSphere treatment, then the first day of the month will be assigned to the missing day

If the imputed AE start date is after the AE stop date (and the stop date is complete), the imputed start date will be reset to the stop date.

The following algorithm should be used to estimate AE stop dates for which only partial information is known:

- Missing year
  - Date left missing
- Missing month
  - Impute “December”
- Missing day
  - Impute last day of that month

Date of birth imputation

The day of date of birth will be imputed as the first day of the month for all missing days. If the month is unknown, it will be imputed as the month before the first TheraSphere treatment.

Date of further anti-cancer treatment imputation

The following algorithm should be used to impute the further anti-cancer treatment start date for which only partial information is known. This includes systemic treatment date, loco-regional treatment date and/or surgery date.

- Missing day and month
  - If the year is the same as the year of initial TheraSphere treatment, then the day and month of the date of TheraSphere will be assigned to the missing fields
- If the year is after the year of initial TheraSphere treatment, then January 1 will be assigned to the missing fields
- Missing month only
- Treat day as missing and replace both month and day accordingly to the procedure above
- Missing day only
- Impute first day of that month

FACT-HEP

If there are missing items, subscale scores can be prorated. This is done by multiplying the sum of the subscale by the number of items in the subscale, then dividing by the number of items actually answered. This can be done on the scoring guide or by using the formula below:

Prorated subscale score **=** [Sum of item scores] **x** [N of items in subscale] / [N of items answered]

When there are missing data, prorating by subscale in this way is acceptable as long as more than 50% of the items were answered (e.g., a minimum of 4 of 7 items, 4 of 6 items, etc). The total score is then calculated as the sum of the un-weighted subscale scores. The FACT scale is considered to be an acceptable indicator of patient quality of life as long as overall item response rate is greater than 80% (e.g., at least 22 of 27 FACT-G items completed). This is not to be confused with individual subscale item response rate, which allows a subscale score to be prorated for missing items if greater than 50% of items are answered. In addition, a total score should only be calculated if ALL of the component subscales have valid scores.
