# Supplementary material for: Effectiveness and safety of selective internal radiation therapy using yttrium-90 glass microspheres for hepatocellular carcinoma: real-world results from the multi-center prospective PROACTIF cohort of 989 patients
Source: eClinicalMedicine. 2026 Apr 17;95:103884. doi: 10.1016/j.eclinm.2026.103884 (PMC13098352; doi:10.1016/j.eclinm.2026.103884)
Supplement: PROACTIF Protocol [file mmc2.pdf]

**Protocol Number:**	BTG-007996-01
**Protocol Short Title:**	PROACTIF
**Protocol Name:**	A **Pro**spective, Post **A**pproval, Multiple Centre**,** Open-Label, Non-Interventional, Registry Study to Evaluate Effe**c**tiveness of **T**heraSphere™ in Cl**i**nical Practice in **F**rance
**Global Sponsor:**	Biocompatibles UK Ltd, a wholly owned indirect subsidiary of Boston Scientific Corporation<br>Lakeview, Riverside Way,<br>Watchmoor Park,<br>Camberley; Surrey GU13 3YL. UK
**Principal Investigators:**	Etienne Garin, MD, PhD<br>Nuclear Medecine specialist<br>Centre Eugène Marquis- CS44229<br>35042 Rennes-France<br>e.garin@rennes.unicancer.fr<br><br>Boris Guiu, MD, PhD<br>Coordinating Investigator for France<br>Interventional Radiology and Radiology Specialist<br>Centre Hospitalier Universitaire de Montpellier<br>191 avenue du Doyen Gaston Giraud<br>34295 Montpellier cedex 5 - France<br>b-guiu@chu-montpellier.fr
**Protocol Project Physician:**	Eveline Boucher, MD.<br>Medical Director, Interventional Oncology<br>Biocompatibles UK Ltd,<br>Lakeview, Riverside Way, Watchmoor Park,<br>Camberley, Surrey, GU15 3YL. UK<br>Telephone:+33 (0)637 690 400<br>evelyne.boucher@bsci.com
**Investigational Product**	TheraSphere™ - Yttrium-90 Glass Microspheres
**Protocol ver. 4.0 Approval Date:**	30 June 2020

This document is the confidential property of the Sponsor. No part of it may be transmitted, reproduced, published, or use by other persons without prior written permission.

Table 1: Protocol Revision History

VERSION NUMBER	AMENDMENT APPROVAL DATE	BRIEF DESCRIPTION OF CHANGES
1.0	23 August 2018	• Original protocol
2.0	03 May 2019	• Protocol Approval and release signature page – change in BTG Clinical Development and Statistical Representative<br>• Reference to steering committee charter added<br>• Study periods have been clearly defined with timelines<br>• Study Scheme updated to provide clear flow for the study<br>• Schedule of assessments – updated with documentation of TheraSphere® contraindication information and data collection post Final Visit added<br>• QoL and survival status will be continue to be collected until end of study (31 Dec 2024)<br>• Added QoL collection is in addition to standard of care<br>• QoL Questionnaire – procedure for collecting QoL added<br>• General Description of Study Device – further device information added<br>• Updated information on conditions for TheraSphere® use and the plan for the data collection<br>• Contraindications to TheraSphere® treatment clarified<br>• Secondary objectives – added treatment procedure (dosimetry)<br>• Dosimetry outcome measures have been separated from secondary outcome measures and more clearly defined<br>• Further details on dosimetry: method for liver volume and volumes of interest assessments<br>• The provision of simplicit^{90}Y™ software is provided by Biocompatibles UK Ltd<br>• Post TS treatment – updated with collection information<br>• Analysis subgroups streamlined to ensure a sufficient number of patients per subgroups to perform statistical analysis<br>• Minor wording and typographical corrections<br>• Minor amendments and additions to Abbreviation list
3.0	23 June 2020	• TheraSphere® amended to TheraSphere™ to be inline with the instructions for use (IFU).<br>• Abbreviation List updated<br>• Protocol updated to include data collection and analysis for new reimbursed indications: mCRC and iCC, this includes;<br>o Additional reimbursement criteria<br>o Additional tumour markers<br>o Analysis for additional indications<br>o Updated study schema

		◦ *Background information: mCRC & iCC*<br>◦ *Central dosimetry review extended to iCC patients*<br>• *Introduction of data collection periods: Initial 12 Month Period and Post 12 Month Follow-Up Period*<br>• *AE follow-up updated to allow for follow-up up to first follow-up visit if not performed within 90 days of treatment*<br>• *Only treatment related SAEs are collected after Initial 12 Month Period*<br>• *Add the collection of concomitant systemic treatment information*<br>• *Guidance for determination of VOI and dosimetry calculation removed (to be provided in separate work instruction)*<br>• *Dose recommendations for all indications added*<br>• *Additional wording around the identification of potential patient via TheraSphere ordering system*<br>• *New safety reporting email added*<br>• *Updated data management section to allow for the use of DataTrak and Medidata RAVE*<br>• *Protocol Deviation wording updated*<br>• *Amended instructions for obtaining current IFU*<br>• *Minor typos amended throughout*
4.0	***30 June 2020***	• *Corrected Warnings for TheraSphere Use wording*

BTG

PROTOCOL APPROVAL & RELEASE SIGNATURE PAGE

**Protocol Number:**	BTG-007996-01
**Protocol Short Title:**	PROACTIF
**Protocol Name:**	A **Pro**spective, Post Approval, Multiple Centre**,** Open-Label, Non-Interventional, Registry Study to Evaluate Effectiveness of TheraSphere™ in Clinical Practice in France
**Protocol Version:**	4.0
**Protocol Approval Date:**	30 June 2020

The above-referenced protocol was reviewed and approved for release by the following:

**Approver**	**Signature and Date**
**Global Sponsor:**<br>**Global Market Access**<br>Robert White<br>Manager, IO, HEMA EMEA & UK/Ireland<br>Biocompatibles UK Ltd.	DocuSigned by:<br>Robert White<br>Signer Name: Robert White<br>Signing Reason: I approve this document<br>Signing Time: 01-Jul-2020 | 01:12 PDT<br>85E90A4A667040E38DADC418CE08564E
**Sponsor: Project Physician**<br>Eveline Boucher, MD<br>Medical Director, Interventional Oncology<br>Biocompatibles UK Ltd	DocuSigned by:<br>Eveline Boucher<br>Signer Name: Eveline Boucher<br>Signing Reason: I approve this document<br>Signing Time: 01-Jul-2020 | 01:35 PDT<br>D1BA4A32376741E0AF8A3FED7F22E389
**Sponsor: Clinical Development**<br>Henk Tissing,<br>VP, IO Clinical Development<br>Biocompatibles UK Ltd	DocuSigned by:<br>Henk Tissing<br>Signer Name: Henk Tissing<br>Signing Reason: I approve this document<br>Signing Time: 01-Jul-2020 | 01:14 PDT<br>4A8BA900BBCB479F9CFC6635678E560B
**Sponsor: Statistician**<br>Binal Patel,<br>Senior Manager Biostatistics<br>Biocompatibles UK Ltd	DocuSigned by:<br>Binal Patel<br>Signer Name: Binal Patel<br>Signing Reason: I approve this document<br>Signing Time: 01-Jul-2020 | 04:52 PDT<br>ABEC98336D00441298B4B00DAC0BE220

GLOBAL PRINCIPAL INVESTIGATOR SIGNATURE

**Protocol Number:**	BTG-007996-01
**Protocol Short Title:**	PROACTIF
**Protocol Name:**	A **Pro**spective, Post **A**pproval, Multiple Centre**,** Open-Label, Non-Interventional, Registry Study to Evaluate Effe**c**tiveness of **T**heraSphere™ in Cl**i**nical Practice in **F**rance
**Protocol Version:**	4.0
**Protocol Approval Date:**	30 June 2020

The Global Principal Investigators (undersigned) hereby declare that they have read this protocol and agree to its contents.

The undersigned confirm that the study will be conducted and documented in accordance with the Declaration of Helsinki, the protocol, standards of International Council for Harmonisation (ICH) Good Clinical Practice, ISO 14155, applicable laws and regulatory requirements specified in the protocol, and the stipulations of the clinical study agreement.

**Investigator Name (please print)**	**Investigator Signature and Date**
Etienne Garin, MD, PhD	DocuSigned by:<br>Etienne Garin<br>Nom du signataire : Etienne Garin<br>Raison de la signature : I approve this document<br>Heure de signature : 01-juil.-2020 | 08:34 PDT<br>38646D401DC744348A36205A7610C03D
Boris Guiu, MD, PhD	DocuSigned by:<br>Boris Guiu<br>Nom du signataire : Boris Guiu<br>Raison de la signature : I approve this document<br>Heure de signature : 02-juil.-2020 | 04:44 PDT<br>BD404409977944BBB15255445EF5B3D3

COUNTRY COORDINATING INVESTIGATOR SIGNATURE

**Protocol Number:**	BTG-007996-01
**Protocol Short Title:**	PROACTIF
**Protocol Name:**	A **Pro**spective, Post **A**pproval, Multiple Centre**,** Open-Label, Non-Interventional, Registry Study to Evaluate Effe**c**tiveness of **T**heraSphere™ in Cl**i**nical Practice in **F**rance
**Protocol Version:**	4.0
**Protocol Approval Date:**	30 June 2020

The Country Coordinating Investigator (undersigned) hereby declares that he/she has read this protocol and agrees to its content.

The undersigned confirms that the trial will be conducted and documented in accordance with the Declaration of Helsinki, the protocol, standards of Good Clinical Practice, applicable laws and regulatory requirements specified in the protocol, and the stipulations of the clinical trial agreement.

Investigator Name (please print): ______________________________

Investigator Signature: ______________________________

Date (DD/MMM/YYYY): ______________________________

INVESTIGATOR PROTOCOL REVIEW STATEMENT

**Protocol Number:**	BTG-007996-01
**Protocol Short Title:**	PROACTIF
**Protocol Name:**	A **Pro**spective, Post **A**pproval, Multiple Centre**,** Open-Label, Non-Interventional, Registry Study to Evaluate Effe**c**tiveness of **T**heraSphere™ in Cl**i**nical Practice in **F**rance
**Protocol Version:**	4.0
**Protocol Approval Date:**	30 June 2020

The site Principal Investigator (undersigned) hereby declares that he/she has read this protocol and agrees to its contents.

The undersigned confirms that the study will be conducted and documented in accordance with the Declaration of Helsinki, the protocol, standards of Good Clinical Practice, applicable laws and regulatory requirements specified in the protocol, and the stipulations of the clinical study agreement.

By written consent to this protocol, the investigator agrees to the above and to fully co-operate with all monitoring and audits in relation to this study by allowing direct access to all documentation, including source data, by authorised individuals representing Biocompatibles UK Ltd, and IRB/IECs and/or by regulatory authorities.

Investigator Name (please print): ______________________________

Investigator Signature: ______________________________

Date (DD/MMM/YYYY): ______________________________

TABLE OF CONTENTS

PROTOCOL SYNOPSIS 14

2.0 STUDY SCHEME 26

3.0 SCHEDULE OF VISITS AND ASSESSMENTS 27

4.0 BACKGROUND AND RATIONALE 30

4.1 Disease Background and Clinical Summary of the Study Device Experience in HCC 30
4.2 Ongoing TheraSphere Clinical Program in HCC 33
4.3 Disease Background and Clinical Summary of the STudy Device Experience of mCRC 33
4.4 Ongoing TheraSphere Clinical Program IN mCRC 34
4.5 Disease Background and Clinical Summary of the Study Device Experience in iCC 34
4.6 Ongoing Therasphere Clinical Program in iCC 36
4.7 General Description of Study Device 36
4.8 Rationale for the post Registration study 36
4.9 Rationale for the study Design and relevancy of data collected. 37
4.10 Dosimetry 37

5.0 STUDY OBJECTIVES 41

5.1 The Primary objective 41
5.2 The Secondary objectives: 41
5.3 Study Design 41
5.4 Primary Outcome measures 42
5.5 Secondary Outcomes measures 42
5.6 Dosimetry Outcomes measures 43

6.0 PATIENT SELECTION 43

6.1 Patient Population 43
6.2 Indications and Conditions for TheraSphere Reimbursement 43
6.3 Contraindications for TheraSphere treatment 45
6.4 Warnings for TheraSphere Use 45
6.5 Study duration 45

7.0 TREATMENT AND FOLLOW UP OF PATIENTS 46

7.1 Study Product 46
7.2 Opposition for data collection 46

7.3 Pre-Treatment procedures 46
7.4 TheraSphere Treatment 46
7.5 Post TheraSphere treatment 47
7.6 AdditionalTreatment(s) 47
7.7 Final visit 47
7.8 Lost to Follow-up patient 47
7.9 Quality of Life Collection 47
7.10 Survival 48
8.0 MEASUREMENTS AND EVALUATIONS 48
8.1 General Considerations 48
8.2 Multi Compartment Dosimetry Assessment 48
8.3 Liver Volume assessment, Volumes of interest 49
8.3.1 Volumes of Interest (VOI) 49
8.3.2 Multi Compartment Pre treatment dosimetry 49
8.3.3 Dose Recommendation 50
8.3.4 Multi Compartment Post-Treatment Dosimetry 51
8.4 Scientific Steering Committee (SSC) 52
8.5 Quality of life questionnaire 52
8.6 Data Collection Schedule and Assessments (see section 3.0) 52
8.6.1 Identification of patients eligible for data collection 52
8.6.2 Patient Registration in the database (figure 2) 53
8.6.3 Eligibility: Patient Information process for non opposition 54
8.6.4 Baseline / Visit 1 54
8.6.5 Treatment Visit Data Collection / Visit 2 55
8.6.6 Initial 12 Month Period: Post-Treatment Follow-up Visits (collected until 12 months) 57
8.6.7 Final Visit (or to be completed at study withdrawal) 57
8.6.8 Follow-up Period: Post treatment follow-up after 12 months 58
8.6.9 Survival Status: Deceased or alive 59
9.0 ADVERSE EVENTS 60
9.1 Contact for Vigilance: 60
9.2 Adverse Event (AE) Definitions 60
9.3 Definitions of SAE/SADE/ADE/USADE/device defficiency for devices 60

9.3.1 Serious Adverse Event (SAE) ... 60

9.3.2 Serious Adverse Device Effect (SADE) ... 60

9.3.3 Adverse Device Effect (ADE) ... 61

9.3.4 Unanticipated Adverse Device Effect (UADE)/Unanticipated Serious Adverse Device Effect (USADE) ... 61

9.3.5 Device Deficiency ... 61

9.4 Recording of AEs and SAEs ... 62

9.5 Causality (Relationship to Device or Procedure) Assessment ... 62

9.6 Suspected Device Malfunction or Deficiency ... 63

9.7 Submitting Expedited Safety Reports ... 63

9.8 Anticipated Adverse Events ... 63

10.0 STATISTICAL CONSIDERATIONS ... 65

10.1 Study Design and Determination of Sample Size ... 65

10.2 Statistical Analysis ... 65

10.2.1 Analysis Populations and Sub-Groups ... 65

10.3 Baseline and Demographic Characteristics ... 66

10.4 Effectiveness Analyses ... 66

10.5 Safety Analyses ... 67

10.6 Dosimetry Analyses ... 67

10.7 Quality of Life ... 68

10.8 Other Analyses ... 68

10.9 Interim Analyses ... 68

10.10 Final Analyses ... 69

11.0 DATA MANAGEMENT ... 70

12.0 LEGAL/ETHICS AND ADMINISTRATIVE PROCEDURES ... 71

12.1 Good Clinical Practice/Regulatory Compliance ... 71

12.2 Study Site and Investigator Qualification ... 71

12.2.1 Statement of Investigator ... 71

12.2.2 Site qualifications ... 71

12.3 Independent Ethics Committee (Iec) ... 72

12.4 Information document and data collection non-opposition ... 72

12.5 Patient Privacy and Confidentiality ... 72

12.6 Study Monitoring ... 73
12.7 Modification of the Registry ... 73
12.8 Protocol Deviations ... 74
12.9 Recording Access to and Retention of Source Data ... 74
12.10 Electronic Case Report Forms ... 74
12.11 Publications ... 75
12.12 Audit/Inspections ... 75
13.0 APPENDICES ... 76
13.1 Appendix 1: Table of Classification of Portal Vein Thrombosis (PVT) ... 76
13.2 Appendix 2: ECOG PERFORMANCE STATUS ... 77
13.3 Appendix 3: HCC Stage and Scores... 78
13.4 Appendix 4: ALBI Score ... 79
13.5 Appendix 5: Categorisation of Agreement between CT/MRI baseline imaging and ^{99m}Tc-MAA deposition and TheraSphere deposition on SPECT ... 80
13.6 Appendix 6: TheraSphere Instructions For Use ... 81
13.7 Appendix 7: Ascites assessment ... 82
14.0 References ... 83

LIST OF TABLES

Table 1: Protocol Revision History ... 2
Table 2: Terms, Acronyms, Abbreviation ... 12
Table 3: Anticipated Serious adverse device effects defined as related to the dose of TheraSphere administered ... 61
Table 4: Adverse Events reported as TheraSphere device or procedure-related ... 64
Table 5: Schedule of Study Analyses ... 68

LIST OF FIGURES

Figure 1: Barcelona Clinic Liver Cancer (BCLC) Staging Classification ... 30
Figure 2: Patient Identification Flow ... 53

Table 2: Terms, Acronyms, Abbreviation

The following abbreviations and specialist terms are used in this protocol.

AASLD	America Association for the Study of Liver Diseases
ADE	Adverse Device Event
AE	Adverse Event
AFP	Alphafetoprotein
ALBI	Albumin/bilirubin
ALT	Alanine Aminotransferase
AST	Aspartate Aminotransferase
BCLC	Barcelona Clinic Liver Cancer
BMI	Body Mass Index
CA 1-9-9	Carbohydrate Antigen 19-9
CEA	Carcinoembryonic antigen
CI(s)	Confidence Interval(s)
CBCT	Cone Beam Computed Tomography
CM	Centimetre
CP	Child Pugh
CPN	Complete Pathologic Necrosis
CR	Complete Response
CT	Computed Tomography
DMP	Data Management Plan
DM	Data Management
EASL	European Association for the Study of the Liver
EC	Ethics Committee
ECOG	Eastern Cooperative Oncology Group
(e)CRF	(Electronic) Case Report Form
EDC	Electronic Data Capture
EHD	Extrahepatic Disease
FACT-Hep	Functional Assessment of Cancer Therapy-Hepatobilary
FLR	Future Liver Remnant

GBq	GigaBecquerel
GCP	Good Clinical Practice
Gy	Gray – a measure of absorbed dose
HAS-CNEDiMTS	Haute Autorité de Santé-Commission Nationale D'évaluation Des Dispositifs Medicaux Et Des Technologies De Santé
HCC	Hepatocellular Carcinoma
HDE	Humanitarian Device Exemption
HUD	Humanitarian Use Device (HUD)
iCC	Intra-hepatic Cholangiocarcinoma
ICH	International Council for Harmonisation
ID	Identification
IEC	Independent Ethics Committee
IFU	Instructions for Use
INR	International Normalised Ratio
ISO	International Organisation for Standardisation
LPPR	Liste de produits et prestations remboursables (List of reimbursable products and services)
MCi	Millicurie
mCRC	Metastatic Colorectal Cancer
MEDDEV	Medical Devices Directive
MedDRA	Medical Dictionary for Regulatory Activities
MIRD	Medical Internal Radiation Dose
µm	Micrometre
mL	Milliliter
Mm	Millimeter
mRECIST	Modified Response Evaluation Criteria in Solid Tumours
MRI	Magnetic Resonance Imaging
MTB	Multi-disciplinary Tumour Board
NCI-CTCAE	National Cancer Institute-Common Terminology Criteria for Adverse Events
OPTN	Organ Procurement and Transplantation Network

ORR	Objective Response Rate
OS	Overall Survival
PD	Progressive Disease
PDs	Protocol Deviations
PET	Positron Emission Tomography
PR	Partial Response
PVTT	Portal Vein (Tumour) Thrombosis
QoL	Quality of life
RECIST	Response Evaluation Criteria in Solid Tumours
RIPH	Recherche Impliquant la Personne Humaine (Research Involving Human Persons)
SADE	Serious Adverse Device Event
SAE	Serious Adverse Event
SAP	Statistical Analysis Plan
SSC	Scientific Steering Committee
SD	Stable Disease
SIRT	Selective Internal Radiation Therapy
SIV	Site Initiation Visit
SPECT	Single Photon Emission Computed Tomography
^{99m}Tc MAA	Technetium-99m Macroaggregated albumin
TACE	Transarterial Chemoembolisation
TTP	Time to Progression
UADE	Unanticipated Adverse Device Effect
UNOS	United Network for Organ Sharing
USADE	Unanticipated Serious Adverse Device Effect
U.S.	United States
v.	Version
VOI	Volumes of Interest
WHO	World Health Organisation
Y-90, Y-89, Y-91	Yttrium-90 and isotopes

PROTOCOL SYNOPSIS

**Protocol Number:**	BTG-007996-01
**Protocol Short Title:**	PROACTIF
**Protocol Name:**	A **Pro**spective, Post-**A**pproval, Multiple Centre**,** Open-Label, Non-Interventional, Registry Study to Evaluate Effe**c**tiveness of **T**heraSphere™ in Cl**i**nical Practice in **F**rance
**Protocol Number:**	BTG-007996-01
**Study Product**	TheraSphere™ Yttrium-90 Glass Microspheres
**Type of Study**	Prospective, non-interventional, post-approval study. Recherche Impliquant la Personne Humaine de catégorie 3 (RIHP 3)
**Study Rationale**	On 20 February 2018, the Commission Nationale d'evaluations des dispositifs Médicaux et des Technologies de Santé (CNEDiMTS) issued a positive recommendation to the Haute Autorité de Santé (HAS) to reimburse TheraSphere in the treatment of Hepatocellular Carcinoma (HCC) for a period of five years. This recommendation was conditional on the collection of survival and health-related quality of life (QoL) data in a post registration study to support the extension of this reimbursement beyond five years.<br><br>On 18 February 2020 the CNEDiMTS issued a positive recommendation to the HAS expanding the reimbursement of TheraSphere to treat metastatic colorectal cancer (mCRC) and intra hepatic cholangiocarcinoma (iCC) until 01 Jan 2024. This recommendation was conditional on the collection of survival and health-related QoL data in a post registration study to support the extension of this reimbursement beyond the 01 Jan 2024.<br><br>This post registration study (registry) will be conducted to provide valuable insight into the management of HCC, mCRC and iCC treated with TheraSphere in a real world clinical practice setting in France.<br><br>The study is designed to capture:<br>• Diversity of patient selection and treatment decision<br>• Disease presentation<br>• Treatment procedures<br>• Treatment effectiveness including, but not restricted to, survival<br>• Safety<br>• QoL<br>• Dosimetry parameters that are associated with treatment efficacy & safety<br><br>Patient and tumour characteristics at baseline and physician/patient expectations are important treatment decision factors which need to be assessed.

	Regarding treatment procedure, this registry is a unique opportunity to provide guidance and training to the French hospitals involved in the treatment of patients with TheraSphere in order to achieve excellence in treatment delivery, as it has been demonstrated in HCC and iCC (retrospectively, and in numerous publications) that multi compartment dosimetry treatment is the best way to optimise treatment outcomes.<br><br>The assessment of QoL broadens the evaluation of medical treatments assessing factors other than those bio-medically related. This is particularly important when the disease prognosis is poor or when alternative treatments have equivalent effectiveness but different tolerability. Information collected from the QoL questionnaires will help in treatment decision-making. They are also useful tools in identifying supportive care needs and improving the global care of cancer patients. QoL is both a clinically and physiologically meaningful endpoint and is best defined from the patient's perspective.<br><br>The results from this registry could potentially create a place for TheraSphere in the Barcelona Clinic Liver Cancer (BCLC) treatment algorithm for HCC and the European and U.S. guidelines for iCC and mCRC and prompt research into its use in alternative treatment indications.
**Study Objectives**	The purpose of this registry study is to gather effectiveness, QoL and safety information on the current clinical use of TheraSphere in France for the renewal of reimbursed indications.
**Primary outcome measures**	1. Overall survival (OS)<br>2. QoL before and after treatment using the FACT-Hep questionnaire
**Secondary outcome measures**	1. Serious Adverse Events (SAEs) graded using the National Cancer Institute-Common Terminology Criteria for Adverse Events version 5.0 (NCI-CTCAE v 5.0).<br>2. Grade 3 or higher adverse events (AEs) (graded using NCI-CTCAE v 5.0) related or possibly related to the device, or the device administration procedure, that occur up to 90 days after every TheraSphere administration or the first follow-up visit (if after 90 days).<br>3. Number and duration of re-hospitalisations related to TheraSphere treatment up to 30 days after first treatment administration.<br>4. Description of treatment expectation (e.g. benefit to survival and disease control) before administration of TheraSphere and number of patients achieving treatment expectation.<br>5. Qualitative tumour response assessment (Index lesion response and overall response): number of patients having complete response (CR), partial response (PR), stable disease (SD), progressive disease (PD).<br>6. Target tumour marker response defined as a ≥50% decrease in:<br>a. AFP levels for patients with a baseline AFP level ≥200 ng/mL.<br>b. CA 19-9 levels for patients with a baseline CA 19-9 level ≥ twice the upper limit of normal.<br>c. CEA levels for patients with a baseline CEA level ≥ twice the upper limit of normal.

	7. Number of patients receiving a post TheraSphere anti-cancer treatment, including surgery.<br>8. Number of patients receiving a post TheraSphere best supportive care treatment.<br>9. Description of vascular access (radial/femoral) used to administer TheraSphere.
**Dosimetry Outcome Measures**	1. Association between tumour(s) location at baseline and location of lesions targeted by Technetium-99m Macroaggregated albumin (^{99m}Tc-MAA) Single Photon Emission Computed Tomography (SPECT) or ^{99m}Tc-MAA SPECT/CT*.<br>2. Association between tumour(s) location at baseline and location of lesions targeted by Y-90 Positron Emission Tomography (PET)/CT or Y-90 PET/MRI or Y-90 SPECT/CT*.<br>3. Association between tumour(s) location based on ^{99m}Tc-MAA (SPECT or SPECT/CT), and location of tumour targeted by Y-90 using post-treatment (PET/CT or PET/MRI or SPECT/CT)*.<br>4. Association between Portal Vein Thrombosis (PVT) at baseline and PVT targeted by ^{99m}Tc-MAA (SPECT or SPECT/CT), Y-90 (PET/CT or PET/MRI or SPECT/CT).<br>5. Association between tumour and normal tissue liver absorbed doses, determined with ^{99m}Tc-MAA (SPECT or SPECT/CT), with qualitative tumour response (CR or PR), OS and safety, respectively.<br>6. Association between tumour and normal tissue liver absorbed doses, determined with Y-90 (PET/CT or PET/MRI), with qualitative tumour response (CR or PR), OS and safety, respectively.<br>7. Association between tumour and normal tissue liver absorbed doses determined with ^{99m}Tc-MAA (SPECT or SPECT/CT) and with Y-90 (PET/CT or PET/MRI).<br>8. Determination of Dose volume histogram (DVH) for total perfused tumour, Index lesion and whole normal liver tissue, using ^{99m}Tc-MAA (SPECT or SPECT/CT) and Y-90 (PET/CT or PET/MRI).<br>* A score that describes the intensity of distribution of ^{99m}Tc-MAA / Y-90 in tumour versus normal tissue, and the tumour coverage with ^{99m}Tc-MAA / Y-90 in tumour will be created (Appendix 5)<br><br>Dosimetry measures will be assessed locally by investigator for all patients and centrally for HCC and iCC patients. Images used for dosimetry assessments will be uploaded to a central imaging database and reviewed independantly
**Study duration**	<u>**Enrolment period:**</u> Five years – 01 Jan 2019 to 31 Dec 2023.<br>• **Day One** is the date of publication of reimbursement of TheraSphere on the *liste des produits et prestations remboursables* (LPPR) list. (01 Jan 2019)<br>• **Date of enrollment close** will be five years after Day One (31 Dec 2023).<br><br><u>**Data collection duration**</u>:<br>• **Start of Data collection:** Data collection will start at each site following the site initiation visit (SIV) and site activation. The availability of reimbursement could occur before sites are activated. In such instances, to ensure the most complete data collection to meet HAS-CNEDiMTS requirements, the data from those patients treated before the SIV date / site activation will be entered into the eCRF retrospectively.

	• **Date of last data collection:** latest 12 months follow-up visit available for the study (~31 Dec 2024).<br><br>**Duration of participation for patient:**<br>• Every patient will participate in the study from Visit 1, prior to initial TheraShere administration until end of study (31 Dec 2024), or death or date when the patient opposes to further participate in the study, whichever comes first.<br>**Data collection periods (see schedule of Visits and Assessments in Section 3.0):**<br>• **Initial 12 Month Period:** Clinical, biological, treatment, safety, QoL and imaging data will be collected from Visit 1, prior to initial TheraSphere administration, until the 12 month follow-up visit.<br>• **Follow-Up Period:** QoL, ECOG, safety data and survival status will be collected at every standard of care follow-up visit until the end of the study.<br>• **Data collection in the above study periods will be collected until:**<br>• the patient opposes to further data collection<br>• the patient withdraws from the registry study (will not attend any further TheraSphere follow up visits), has started another cancer treatment, received best supportive palliative care, or follow-up is no longer possible for any reason<br>• the patient has died<br>• At the end of the study (31 Dec 2024), survival status, including cause and date of death, and any subsequent treatment received since last TheraSphere treatment will be collected<br><br>Study duration: 6 years<br>Final Study report: ~June 2025
**Study Design**	Prospective, non-interventional, single arm, open label, post registration, single territory, multi-centre, study based in France.
**Target Population**	All the patients for whom a treatment with TheraSphere has been prescribed and reimbursed.
**Number of Patients**	>500 patients
**Number and Location of Sites**	All sites certified for TheraSphere use: Approximately 30 sites across France
**Eligibility for Data collection**	• Patient who has not refused data collection<br>• Patient who will receive a reimbursed dose of TheraSphere
**Indications and conditions for TheraSphere reimbursement**	**Indications for reimbursement**<br><br>Patients with HCC who meet the following criteria:<br>1. Confirmed HCC, by histology or America Association for the Study of Liver Diseases (AASLD) or EASL imaging criteria<br>2. Patient scheduled to receive TherasSphere treatment per Multi-disciplinary Tumour Board (MTB) decision

3. Treatment given as a palliative intent (patient not eligible* for resection or ablation)
4. Patient who is BCLC B or BCLC C or with PVT** (Appendices 1 and 3)
5. Patient who is not eligible* for, or has failed sorafenib treatment
6. Patient with good general status (ECOG score 0 or 1) (Appendix 2)
7. Patient with preserved liver function*** (Child Pugh A-B) (Appendix 3)

* Treatment not possible or not recommended
** Portal vein invasion by tumour
***Preserved liver function: includes patients with different degrees of liver functional reserve (non treated liver) that has to be carefully evaluated. Compensated liver disease (without ascites) is required to obtain optimal outcome.
(Forner et al. 2018; EASL Guidelines 2018)

Patients with mCRC who meet the following criteria:

1. Patient scheduled to receive TherasSphere treatment per MTB decision
2. Preserved general health condition (ECOG score ≤ 2).
3. Hepatic tumour load (<25%)
4. Absence of extrahepatic disease (except in situ primary colorectal cancer)
5. Refractory or intolerant to all approved intra venous and oral therapies for colorectal cancer. Progression under chemotherapy should be documented

Patients with iCC who meet the following criteria:

1. Patient scheduled to receive TherasSphere treatment per MTB decision
2. First line palliative treatment for iCC
3. Patient unresectable at diagnosis or in a recurrence after resection
4. With or without association with chemotherapy
5. Preserved general health condition (ECOG ≤ 1) when treated with TheraSphere with concomitant chemotherapy
6. Preserved general health condition (ECOG score ≤ 2) when treated with TheraSphere alone
7. Absence of extrahepatic disease
8. Hepatic tumour load <50%
9. Patient with preserved liver function (Child-Pugh score A or B in case of cirrhosis).

Conditions for TheraSphere use

- The use of TheraSphere will be carried out in accordance with the decree number 2007-389 dated 21 March 2007 relative to the technical operating conditions applicable to cancer care activity.
- TheraSphere is used by multidisciplinary teams that include: a nuclear physician, an interventional radiologist with the expertise of hepatic embolisation in oncology, a radiophysicist and a radiopharmacist. This activity must be carried out in centres with sufficient infrastructure to be authorised by the French Nuclear Safety Authority (Agence de Sécurité Nucléaire - ASN) to carry out internal radiation activities.

	• The decision to perform the treatment and the post-treatment follow-up has been taken under patient agreement and after a positive treatment recommendation of the local/regional MTB specialised in HCC, mCRC and iCC. The MTB must include at least: an oncologist, a hepatologist, or a hepatologist skilled in oncology, a surgeon specialist in liver surgery, an interventional radiologist, a nuclear medicine specialist, a radiation oncologist, and a palliative care specialist.<br>• The patient must have access to a supportive care team.
**Contra-indications and warnings for TheraSphere treatment**	TheraSphere is **contraindicated** in patients who:<br>• ^{99m}Tc-MAA (SPECT/CT or SPECT) shows any deposition to gastro intestinal tract that could not be corrected by angiography techniques.<br>• ^{99m}Tc-MAA (SPECT/CT or SPECT) show shunting to blood to the lung that could result in delivery of greater than 16.5 mCi of yttrium-90 to the lungs, 30 Gy in a single treatment and 50Gy in cumulative treatments.<br>• hepatic artery catheterisation is contraindicated (vascular abnormalities or bleeding diasthesis).<br>• have severe hepatic dysfunction or pulmonary insufficiency.<br>• are pregnant.<br>**Warnings for TheraSphere Use**<br>The physician should always take into consideration any pre-treatment risk factors (listed below) when making the decision treat with TheraSphere:<br>• Patient with bulky disease (measured tumour volume >70% or tumour nodules too numerous to count).<br>• Patient with tumour volume >50% combined with albumin <30g/L.<br>• Patient with infiltrative disease.<br>• Patient with hepatic functional reserve (non treated liver) ≤30% of total liver volume/function and dose to the perfused normal tissue liver ≥120 Gy, determined by ^{99m}Tc-MAA (SPECT or SPECT/CT).<br>• Patient with Portal Vein Thrombosis (PVT) type Vp4 with **complete** main portal vein invasion, whichever the ^{99m}Tc-MAA targeting.<br>**Note: Patients with incomplete main portal vein invasion and good MAA targeting can be considered.**<br>• Bilirubin >2 mg/dL or >34 µmol/L<br>• AST/ALT >5 x ULN<br>• Ascites > grade 1 under well managed diuretic treatment (Appendix 7)<br>• Child Pugh score >B7
**TheraSphere® treatment**	• TheraSphere treatment will be performed according to the Instructions for Use (IFU).<br>• TheraSphere infusion can be selective (tumour feeding artery, liver segment or liver sector) or non-selective (right/left liver, whole liver).<br>• The treatment could be administered in multiple infusions to address vascular abnormalities and tumour distribution (see IFU).

	• If two TheraSphere treatment sessions are required to complete tumour treatment, the liver part (right or left liver) with the highest tumour burden should be scheduled for first treatment. Before the second treatment session, a second angiogram with ^{99m}Tc-MAA scan should be performed. A second treatment would typically take place 30-45 days after the treatment to the first lobe, provided the patient has tolerated the first treatment and the condition for TheaSphere® administration as described above are met.
**Dosimetry**	• TheraSphere treatment planning will be performed according to local procedure (standard/multi compartment dosimetry).<br>• Simplicit^{90}Y™ dosimetry software will be provided to the site, as required. The use of Simplicit^{90}Y™ is preferred but not mandatory.<br>• A central dosimetry assessment is planned for HCC and iCC, the site will upload baseline images (CT/MRI), and images needed to perform dosimetry assessments ^{99m}Tc-MAA SPECT or ^{99m}Tc-MAA SPECT/CT, Y-90 SPECT/CT or Y-90 PET/CT or Y-90 PET/MRI.
**Laboratory Tests**	Collected as part of routine institutional practice:<br>ALT; AST; INR or prothrombin time (PT) or prothrombin ratio; albumin; bilirubin, creatinine and tumour markers (AFP, CA 19-9, CEA).
**Sample Size**	Not applicable for a real world registry; all the patients will be included providing TheraSphere has been reimbursed.
**Statistical Methods**	<u>Analysis Populations and Subgroups</u><br><br>The treated population will comprise of all patients who have received a reimbursed dose of TheraSphere and are not opposed to their data being collected.<br><br>The dosimetry population will comprise of patients in the treated population with dosimetry data available. Dosimetry data will be collected for all patients.<br><br>All analyses will be performed on the treated population, except for dosimetry analyses. All analyses will be performed according to the disease indication (HCC, mCRC and iCC).<br><br>Analyses of study outcomes will also be performed on the following subgroups of interest:<br><br>For all patients:<br>• Age group (≥18 to <65 years, ≥65 to <75 years, ≥75 years)<br>• Unilobar or bilobar disease at baseline<br>• ECOG status (0, >0) at baseline<br>• Albumin/bilirubin (ALBI) score (1 or 2, 3) at baseline<br>• Liver tumour burden at baseline (<25%, ≥25%)<br>• Target lesion size (≤ 5cm vs >5cm, ≤7cm vs >7cm,≤ 10cm vs >10cm)<br>• Selective versus non-selective (lobar or whole liver) administration

	• Standard versus multi compartment dosimetry treatment<br><br>For HCC patients:<br>• Etiology of underlying disease<br>• Child Pugh score (A or B) at baseline for cirrhotic patients<br>• Cirrhosis versus no cirrhosis<br>• Prior TACE treatment (Yes, No)<br>• PVT classification (Vp0, Vp1 or Vp2, Vp3 or Vp4) at baseline<br>• BCLC stage (B, C) at baseline<br>• Prior systemic treatment, including sorafenib (Yes, No)<br>• AFP (<200 ng/ml, ≥200 ng/mL, <400ng/mL, ≥400 ng/mL) at baseline<br>• Threshold absorbed doses to the tumour ≥ 205, < 205 Gy and ≥250, <250 Gy (by local and central assessment)<br><br>For mCRC patients:<br>• CEA (<2xULN, ≥ 2xULN) at baseline<br>• Previous line of systemic chemotherapy (≤ 2, >2)<br>• Prior local or/and locoregional treatment (Yes, No)<br>• Concomitant chemotherapy versus non concomitant chemotherapy<br>• Threshold absorbed doses to the tumour <100 and ≥ 100 Gy (by local assessment)<br><br>For iCC patients:<br>• Prior resection (Yes, No)<br>• CA 19-9 (<2xULN, ≥ 2xULN) at baseline<br>• Cirrhosis versus no cirrhosis<br>• Concomitant chemotherapy versus non concomitant chemotherapy<br>• Threshold absorbed doses to the tumour < 205 Gy, 205-250 Gy, >250 Gy (by local and central assessment)<br><br><u>Baseline and Demographic Characteristics</u><br>Demographic data and baseline characteristics will be summarised. Continuous data will be summarised with means, medians, standard deviations, minima and maxima. Categorical data will be summarised with observed counts and percentages for each category.<br>Length of follow-up will be summarised as mean, median, standard deviation, minimum and maximum. The median length of follow-up will also be computed using the reverse Kaplan-Meier method.<br><br><u>Effectiveness Analyses</u><br>All effectiveness outcomes will be assessed in the treated population.<br><br>A Kaplan Meier (KM) analysis will be performed for OS and median OS will be computed with a corresponding 95% confidence interval (CI).

	Univariable and multivariable Cox regression analyses of OS will be performed to assess the impact of the subgroup factors listed above.<br><br>The number of patients achieving their treatment expectation will be summarised as observed counts and percentages, with corresponding 95% CI.<br><br>Tumour marker response will be summarised as observed counts and percentages, with corresponding 95% CI.<br><br>Qualitative tumour response will be summarised as the number and percentage of patients having a response (CR or PR), with corresponding 95% CI.<br><br>Univariable and multivariable logistic regression analyses of binary effectiveness endpoints (i.e. achievement of treatment expectation, tumour marker response and qualitative tumour response) will be performed to assess the impact of the subgroup factors listed above.<br><br><u>Safety Analyses</u><br><br>Incidence of SAEs and grade 3 or higher AEs, coded according to MedDRA (Medical Dictionary for Regulatory Activities), will be tabulated. Descriptive summaries of laboratory results, including changes from baseline, will be presented by study visit. The number of re-hospitalisations related to TheraSphere treatment will be summarised as observed counts and percentages and the duration of re-hospitalisations will be summarised with means, medians, standard deviations, minima and maxima.<br><br><u>Dosimetry Analyses</u><br>The following outcomes will be summarised as observed counts and percentages for local assessments in all indications and for central assessments in HCC and iCC patients:<br>• Association between tumour location at baseline and location of lesions targeted by ^{99m}Tc-MAA (SPECT or SPECT/CT)*<br>• Association between tumour location at baseline and location of lesions targeted by Y-90 PET/CT, Y-90 PET/MRI or Y-90 SPECT/CT*<br>• Association of lesion location based on ^{99m}Tc-MAA (SPECT or SPECT/CT), and location of lesions targeted by Y-90 using post-treatment PET/CT, PET/MRI or SPECT/CT*<br>• Association between PVT at baseline and PVT targeting by ^{99m}Tc-MAA (SPECT or SPECT/CT), Y-90 (PET/CT, PET/MRI or SPECT/CT), when applicable<br>• DVH for total perfused tumour, index lesion and whole normal liver tissue, using ^{99m}Tc-MAA (SPECT or SPECT/CT) and Y-90 (PET/CT or PET/MRI), when applicable.<br><br>* A score that describe the intensity of distribution of ^{99m}Tc-MAA / Y-90 in the tumour versus normal tissue, and the tumour coverage with ^{99m}Tc-MAA / Y-90 in tumour will be created (Appendix 5)

	In patients with HCC and iCC, a Cox regression analyses of OS will be performed to assess the impact of the tumour absorbed doses. This will be done separately for absorbed doses determined by ^{99m}Tc-MAA (SPECT or SPECT/CT) and by post-treatment Y-90 (PET/CT or PET/MRI). Similarly, in patients with mCRC, a similar cox regression analysis of OS will be performed to assess the impact of absorbed dose to perfused tumours and liver volume.<br><br>In patients with HCC and iCC, a logistic regression analysis of qualitative tumour/index lesion response (CR or PR) will be performed to assess the impact of the tumour/index lesion absorbed doses. This will be done separately for tumour/index lesion absorbed doses determined by ^{99m}Tc-MAA (SPECT or SPECT/CT) and determined by post treatment Y-90 (PET/CT or PET/MRI). Similarly, in mCRC patients, a logistic regression analysis of qualitative tumour/index lesion response will be performed to assess the impact of the absorbed dose to perfused tumours and liver volume.<br><br>In patients with HCC and iCC, a logistic regression analyses of the occurrence of SAEs will be performed to assess the impact of the normal tissue liver absorbed doses. This will be done separately for absorbed doses from pre-procedural ^{99m}Tc-MAA (SPECT or SPECT/CT) and for post-treatment Y-90 (PET/CT or PET/MRI). Similarly, in patients with mCRC, logistic regression analyses of the occurrence of SAEs will be performed to assess the impact of absorbed dose to normal tissue liver.<br><br>In patients in HCC and iCC, the relationship between absorbed doses derived from post-treatment Y-90 (PET/CT or PET/MRI) and from pre-procedural ^{99m}Tc-MAA (SPECT or SPECT/CT) will be assessed separately for normal tissue liver absorbed doses and tumoural liver absorbed doses (perfused liver and total liver) and tumour absorbed doses using Bland-Altman analysis. Similarly, in patients with mCRC, the relationship between absorbed dose to perfused liver volume will be assessed for absorbed dose to perfused liver volume and absorbed dose to normal tissue liver.<br><br>In patients with HCC and iCC, a linear regression of absorbed dose from pre-procedural ^{99m}Tc-MAA (SPECT or SPECT/CT) imaging and post-treatment Y-90 (PET/CT or PET/MRI) imaging will be performed and Pearson's correlation coefficient will be calculated. Similarly, in patients with mCRC, a linear regression will also be performed for absorbed dose to perfused liver volume and absorbed dose to normal tissue liver.<br><br><u>Quality of Life analysis</u><br>QoL scores of each domain and each question at each time-point and their differences from baseline will be summarised. A deterioration in QoL is defined as a 7-point decline in the total score or death, whichever comes first. The time to deterioration in QoL will be calculated as the interval between the date of first TheraSphere treatment and deterioration in QoL. If a patient is lost to follow-up, the

<table>
<tr><td></td><td>patient will be considered as a death in the analyses. A KM analysis will be performed and median value will be computed with corresponding 95% CI.

<u>Other Analyses</u>
The following outcomes will be summarised as observed counts and percentages:
• The number of patients receiving a post TheraSphere anti-cancer treatment.
• The number of patients receiving a post TheraSphere best supportive care treatment.
• The type of vascular access (radial/femoral) used to administer TheraSphere.

<u>Interim and Final Analyses</u>
Interim analyses will be performed when sufficient data have been completed in the eCRF to support interim study publications.

<table><tr><th>Analysis number</th><th>Based on first patient enrolled until:</th><th>Data to be included in analysis:</th></tr><tr><td>1st Interim analysis</td><td>1 year</td><td>Baseline characteristics, TheraSphere treatment and safety data</td></tr><tr><td>2nd Interim analysis</td><td>2 years</td><td>All data</td></tr><tr><td>3rd Interim analysis (5 year clinical study report to HAS)</td><td>4 years</td><td>All data</td></tr><tr><td>Final analysis</td><td>5 years (after last patient enrolled)</td><td>All data</td></tr></table>Note: 1st Interim analysis will only have data available to HCC as this was prior to the addition of iCC and mCRC indications.</td></tr>
<tr><td>Data Collection Visits</td><td>See Schedule Visits and Assessments (Section 3.0).</td></tr>
<tr><td>Scientific Advisory Board Steering Committee</td><td>Members selected based on clinical and technical expertise related to TheraSphere and/or unresectable HCC management will form the Scientific Advisory Board / Steering Committee for the registry. Details can be found in the Steering Committee Charter.</td></tr>
</table>

The study will be conducted and documented in accordance with the Declaration of Helsinki, the protocol, standards of Good Clinical Practice, applicable laws and regulatory requirements specified in the protocol, and the stipulations of the clinical study agreement.

2.0 STUDY SCHEME

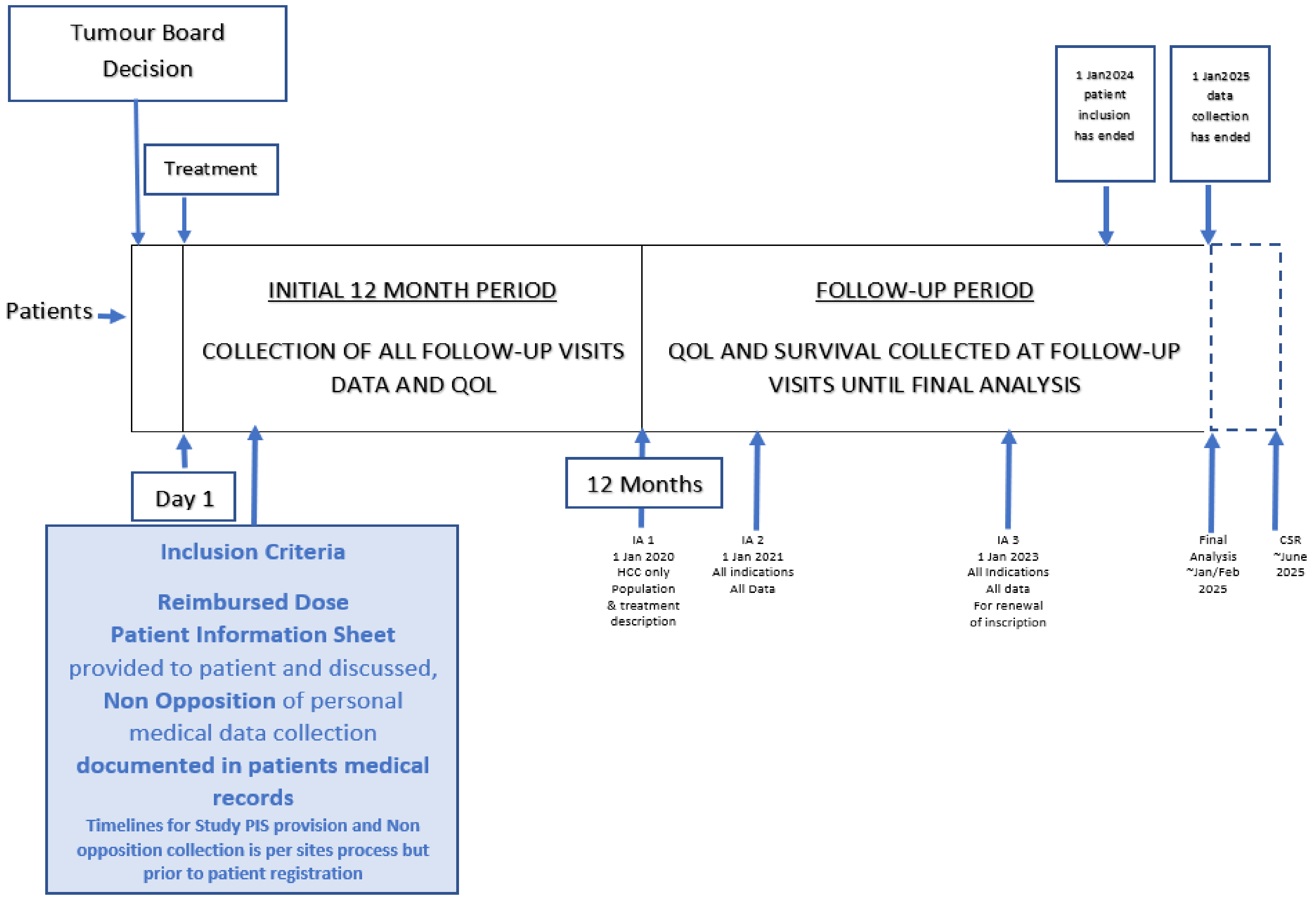

3.0 SCHEDULE OF VISITS AND ASSESSMENTS

Grey: the assessment must be performed within 14 days of pre-TheraSphere angiography

Data or Assessment Recorded	Initial 12 Month Period							Follow-Up Period
	Patient Registration	TheraSphere Treatment				Follow up Visits	Final Visit of Initial 12 month follow-up period [12]	Follow up Visits after 12 months
		Day 1 First treatment		Day 28 Second treatment (If needed)		Every 2/4 months	12 months post initial treatment / Time of study withdrawal	At standard of care follow up visits
	Baseline / Visit 1	Visit 2a		Visit 2b				
		Pre-TS	TS	Pre-TS	TS			
Information to Patient, Documentation of non-opposition to data collection, eligibility[1]	X							
Enrollment, Patient registration and Identification (ID, partial date of birth)	X							
Compliance to HAS requirement and to TheraSphere treatment requirement	X							
Documentation of TheraSphere contraindication	X							
Patient Characteristics (gender, BMI/weight, Main Comorbidities [2])	X							
Set treatment goal / Expectation	X							
**Questionaire QoL (FACT-Hep)**	X					X	X	X
**ECOG Performance Status**	X		X	X	X	X	X (if possible)	X (if possible)
**Disease Characteristics**								
Liver disease description [3]	X							
Liver Cancer History [4]	X							
CT or MRI (Tumour Characteristics[5])	X							
PVT description [6]	X			X		X		
**Liver function score and Tumour score**								
Ascites/Encephalopathy	X			X		X	X	
Child Pugh Score	X			X		X	X	
BCLC stage (HCC patients)	X					X	X	
**Laboratory tests**								
Biochemistry-coagulation (Albumin, Bilirubin, AST, ALT, INR, Prothrombin Ratio or PT), including ALBI score derivation, creatinine	X		X	X	X	X	X	
Tumour Marker: AFP (for HCC), CEA (for mCRC) CA 19-9 (for iCC)	X					X	X	

Data or Assessment Recorded	Initial 12 Month Period							Follow-Up Period
	Patient Registration	TheraSphere Treatment				Follow up Visits	Final Visit of Initial 12 month follow-up period[12]	Follow up Visits after 12 months
		Day 1 First treatment		Day 28 Second treatment (If needed)		Every 2/4 months	12 months post initial treatment / Time of study withdrawal	At standard of care follow up visits
	Baseline / Visit 1	Visit 2a		Visit 2b				
		Pre-TS	TS	Pre-TS	TS			
**TheraSphere Treatment parameters**								
Pre-TheraSphere angiography[7], Coils placement; ^{99m}Tc-MAA administration		X		X				
^{99m}Tc-MAA SPECT or SPECT/CT imaging[8]		X		X				
TheraSphere Administration[9]			X		X			
Y-90 SPECT/CT, PET/CT or PET/MRI imaging[9]			X		X			
Dosimetry: liver/tumour fractions, calculation of absorbed dose to the tumour, perfused liver, non tumoural liver and lung.[10]		X	X	X	X			
Collection of concomitant systemic treatment information, if applicable	X			X		X	X	
**Treatment Follow-up**								
Treatment expectation met, qualitative response assessment [11]	X					X	X	
Subsequent anti-cancer treatment [13]							X	X
Study Withdrawal [14]							X	
Survival[15]						X	X	X
**Adverse Events[16]**								
SAEs		X		X		X		X
AEs grade 3 or higher related to device or device procedure		X		X		X		
Abbreviations: ^{99m}Tc-MAA=Technetium-99m Macroaggregated albumin AE=adverse event; AFP= Alphafetoprotein; ALBI= Albumin/bilirubin; ALT= Alanine Aminotransferase; AST= Alanine Aminotransferase; BCLC=Barcelona Clinic Liver Cancer; BMI=Body Mass Index; CT= Computed Tomography; ECOG=Eastern Cooperative Oncology Group; HCC= hepatocellular carcinoma; INR= International Normalised Ratio; M=month; MRI= Magnetic Resonance Imaging; PET=position emission tomography; PR=Partial Response; PT=Prothrombin Time; PVT= Portal vein (tumour) thrombosis; QoL=Quality of Life; OS= Overall survival; SAE= Serious adverse event; SPECT= single proton emission computed tomography								

Foot Notes:

1. Study information provided to patient and non-opposition to data collection to be documented in the patients record
2. Main Comorbidities: diabetes, hypertension, hyperuricemia, BMI>28 or overweight, coronary disease, pulmonary disease, other relevant disease
3. Liver status: fibrosis, cirrhosis, normal liver. HCC etiology, previous liver decompensation.
4. Histology, date and method of diagnosis, previous liver cancer treatment received
5. CT or MRI imaging, determination of location and number of liver lesions, presence of extra hepatic disease. A recent (within one month prior to TheraSphere treatment) imaging is highly preferable. This imaging will be uploaded to an imaging database for central review)
6. Portal vein thrombosis status according to Ikai classification (Appendix 1)
7. Describe Vascular Access, use of Cone Beam Computed Tomography (CBCT) during angiography procedure is recommended
8. Pre-treatment ^{99m}Tc-MAA SPECT/CT and post-treatment Y-90 SPECT/CT, Y-90 PET/CT or Y-90 PETMRI image scans as part of usual care. Assessment of association of ^{99m}TC-MAA targeting and Y-90 targeting and location of target lesions. The imaging data will be uploaded for central review.
9. Describe type of administration: selective/non selective/whole liver; number of vials, and vial activity administered
10. Dosimetry assessment will be assessed locally for all indications and centrally (uploaded onto a portal for central review) for HCC and iCC.

11. At baseline, a description of expected treatment benefit, thereafter assessment of achievement of this expectation and qualitative assessment of response
12. The Final Visit of the 12 months occurs 12 months after the last TheraSphere treatment, or earlier, if the patient withdraws from the study (discontinues the usual standard of care TheraSphere follow-up i.e, followed in another institution, lost to follow-up, refuses data collection, has started another non TheraSphere treatment or receiving best supportive palliative care)
13. Recorded at the point the patient withdraws from the study, at study close (31 Dec 2024), death or patient opposes further collection of data, whichever occurs sooner.
14. Data collection, except for survival status data, will stop if the patient received a subsequent anti-cancer treatment, received best supportive palliative care, is lost to follow up, or the patient recinded authorisation to collect data. The name of the subsequent anti-cancer treatment and the reason for stopping data collection will be recorded.
15. Survival status data will be collected until death or study close, whichever occurs first.
16. SAEs, related or not, must be collected from any TheraSphere administration during the initial Month 12 period. During the follow-up period only related SAE must be collected. Grade 3 or higher adverse events (AEs) (graded using NCI-CTCAE v 5.0) related or possibly related to the device, or the device administration procedure, that occur up to 90 days after every TheraSphere administration or the first follow-up visit (if after 90 days).

4.0 BACKGROUND AND RATIONALE

4.1 DISEASE BACKGROUND AND CLINICAL SUMMARY OF THE STUDY DEVICE EXPERIENCE IN HCC

The most common primary liver cancer (80%-90%) is hepatocellular carcinoma (HCC) followed by intrahepatic ductal cancers. HCC carries a poor prognosis for many patients given that diagnosis occurs at an advanced disease stage when median survival time is less than one year. Left untreated, HCC is uniformly fatal, with an incidence to death ratio close to 1.0. The mean age at diagnosis is approximately 65 years of age, however, the incidence rate has risen over the past decades and the number of new cases in patients 45-60 years of age has also risen.

HCC is a heterogeneous cancer with patients with solitary and multinodular tumours having varying degrees of tumour burden, unilobar or bilobar involvement, invasion into the liver vascular system, extrahepatic disease (EHD) spread and metabolic liver dysfunction. As HCC is primarily a disease of older patients, HCC is often accompanied by a varying number of comorbidities and states of overall well-being. Cirrhosis is present concomitantly with HCC in approximately 80% of patients. Patients often present when they become symptomatic from advancing cirrhosis at which stage their cancer has progressed such that treatment options may be limited. In the face of progressive liver disease, the differing disease etiologies for HCC and the high molecular variability of HCC, its treatment is unique amongst cancers. These variables introduce an additional level of complexity in evaluating HCC disease and choosing optimal therapy for a patient. Given the presence of both progressive cirrhosis and liver cancer in most HCC patients, optimal treatment should be directed at both preserving liver function and halting cancer progression. Patients must continually be monitored and treatment choices re-evaluated as the dynamics of both diseases change over time.
Current treatment options are either curative or palliative in nature with the choice of therapy dependent on a thorough understanding of the characteristics of a patient's disease from both an HCC and liver cirrhosis perspective. The Barcelona Clinic Liver Cancer (BCLC) staging classification takes both of these disease states into account and provides recommended treatments for HCC across the spectrum of disease severity, cirrhosis and performance status.

Figure 1: Barcelona Clinic Liver Cancer (BCLC) Staging Classification

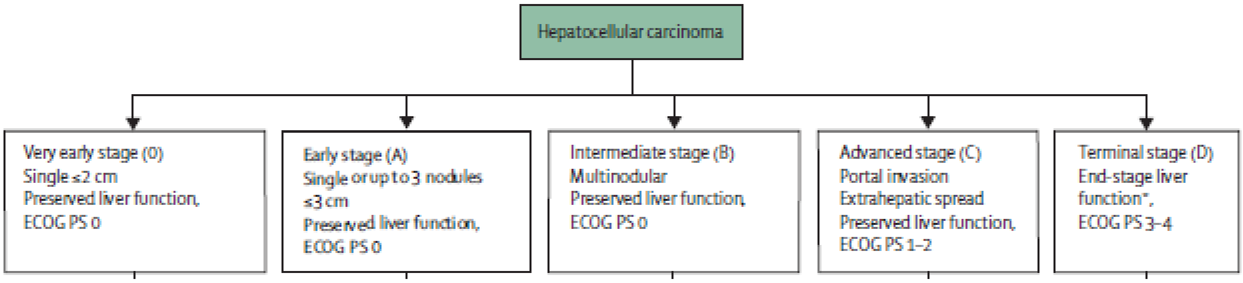

- Portal vein invasion (portal vein tumour thrombosis).

- Preserved liver function: includes a group of patients with different degrees of liver function reserve that has to be carefully evaluated. For most treatment options, compensated liver disease (without ascites) is required to obtain optimal outcomes (Forner et al., 2018).

Numerous case-cohort clinical studies have demonstrated that Selective Internal Radiation Therapy (SIRT) with TheraSphere is an effective locoregional treatment for primary unresectable HCC with or without Portal Vein Thrombosis (PVT). Moreover, there has been a high degree of consistency in the literature in terms of survival outcomes.

Evidence of outcomes of SIRT with Yttrium-90 (Y-90) stems from large, generally, retrospective-cohort studies in unresectable HCC patients, with or without PVT. Long-term experience of TheraSphere in the treatment of patients with HCC was reported in 291 patients as part of a single-center, prospective, longitudinal cohort study (Salem et al. 2016). Toxicities were recorded using the Common Terminology Criteria version 3.0. Response rate and time to progression (TTP) were determined using World Health Organization (WHO) and European Association for the Study of the Liver (EASL) guidelines. Survival by BCLC stage was also assessed. Univariate and multivariate analyses were performed to identify prognostic factors. A total of 526 treatments were administered (mean, 1.8; range, 1-5). Toxicities included fatigue (57%), pain (23%), and nausea/vomiting (20%); 19% exhibited grade 3/4 bilirubin toxicity. The 30-day mortality rate was 3%. Objective response rates (ORRs) were 42% and 57% based on WHO and EASL criteria, respectively. The overall median TTP was 7.9 months (95% confidence interval [CI], 6-10.3) determined by either WHO, EASL, United Network for Organ Sharing (UNOS) stage or PVT status. Median Overall Survival (OS) differed between patients with Child Pugh (CP) A and B disease (A, 17.2 months; B, 7.7 months; P = 0.002). Patients with CP B and PVT had a median OS of 5.6 months (95% CI, 4.5-6.7). Baseline age, gender, performance status, presence of portal hypertension, tumour distribution, levels of bilirubin, albumin, alpha-fetoprotein (AFP) and WHO/EASL tumour response rate predicted survival. These investigators concluded that patients with CP A disease, with or without PVT, benefited most from treatment. Patients with CP B disease and PVT had poor outcomes. TTP and OS varied by patient BCLC stage at baseline.

Hilgard et al, 2010 reported treatment of 108 consecutive patients with advanced HCC using TheraSphere. Median OS was 16.4 months for all patients, with median TTP of 10.0 months determined by Modified Response Evaluation Criteria in Solid Tumours (mRECIST). Objective response rate at 90 days, evaluated by Response Evaluation Criteria in Solid Tumours (RECIST) in 62 patients, was 16%. Toxicities were generally mild to moderate with the most common being transient fatigue during the week following administration (61%) and abdominal pain (56%). One case of radiation cholecystitis was treated with cholecystectomy. There were no grade 3 or 4 lung toxicities. Lymphopenia was observed without clinical sequelae. Because hepatic decompensation is a known risk of Y-90 microsphere therapy, all elevations in bilirubin values were considered to be treatment related hepatotoxicities. For patients with normal bilirubin at enrollment, 32% experienced grade 1 or 2 elevations and three patients developed grade 3 elevations.

The clinical application of TheraSphere expands beyond its use in advanced HCC. The clinical benefit and safety of TheraSphere has been well published in earlier stage HCC disease. TheraSphere can be used as a neoadjuvant or adjuvant treatment in BCLC A and B patients who may then become

eligible for curative treatments such as ablation, resection or transplant. For some transplant-eligible patients, TheraSphere can be used as a “bridge to transplant” agent, slowing or halting the progression of HCC, allowing transplant candidates to remain transplant-eligible. A longer TTP has been demonstrated in such patients and may identify patients who are more likely to benefit from a transplant given that their disease can be monitored, e.g., biological test of time. This treatment benefit has increased relevance as a result of the recent Organ Procurement and Transplantation Network (OPTN)/UNOS revisions requiring a 6-month waiting period prior to being considered for transplant eligibility, and if AFP levels exceed 1000 ng/mL, the guidance recommends locoregional therapy to reduce levels below 500 ng/mL. Used for this purpose, TheraSphere provides an alternative to TACE which often requires hospitalisation, is associated with chemotherapy-related side effects and has a higher rate of post-embolisation syndrome versus TheraSphere. TheraSphere has shown improved tumour response and durability versus TACE extending the time to transplant in a randomised and a case control study (Lewandowski et al., 2009; Salem et al., 2016).

TheraSphere can also be used as a neoadjuvant agent to decrease tumour size or burden such that curative therapies become an option for some patients with otherwise unresectable HCC disease. In patients who have unresectable disease isolated to ≤2 liver segments, SIRT confined to these segments limits normal parenchyma exposure and radiation-associated liver toxicities. This application of TheraSphere, called radiation segmentectomy, is a safe and beneficial technique in solitary HCC tumours ≦5 cm not amenable to ablation, resulting in improved tumour response and pathological necrosis. In comparison to lobar treatments, Y-90 radioembolisation (Y-90) radiation segmentectomy provides a transarterial method of selectively targeting HCC tumours while minimising the normal hepatic parenchyma exposure to toxic effects of radiation. As a result of the segmental treatment approach, segmental doses are higher than the prescribed dose by the ratio of lobar to segmental volumes (Riaz et al., 2011, Vouche et al., 2013). However, the overall dose to the lobe remains within the desired range. Radiation segmentectomy treatment along an arterial anatomic plane may result in complete necrosis of targeted segments and regression of targeted region. More complete necrosis was observed when segmental irradiation dose exceeded 190 Gy suggesting possibility of a threshold dose needed to achieve complete pathologic necrosis (CPN) (Forner et al., 2018).

For patients who have healthy livers but whose disease is not amenable to Transarterial Chemoembolisation (TACE), TheraSphere has been demonstrated to be a viable, and at times preferred, alternative. For patients who have unilobar disease and good liver function but cannot undergo liver resection due to insufficient future liver remnant volume (FLRV), TheraSphere can effectively manage the tumour while the contralateral liver volume increases as a result of SIRT to the treated lobe. This lobar atrophy-hypertrophy phenomenon is referred to as radiation lobectomy and may offer some unresectable HCC patients the opportunity to qualify for curative treatments, i.e., resection. Improvements in the administration technique and the understanding of the importance of optimal dosing, has highlighted the potential for improved outcomes when tumour-targeted dosing is prescribed. Quality of life (QoL) data supports the use of TheraSphere and safety events reported in publications (Salem et al, 2013, Rognoni et al, 2017) remain consistent with the profile for TheraSphere as described in the United States (U.S.) Package Insert.

Fifteen years of clinical experience in 1000 patients was recently reported by Salem et al, 2017. The cohort was stratified by UNOS stage, CP and BCLC status. In CP A patients, median OS for BCLC A was 47.3 (95% CI: 39.5-80.3) months, BCLC B 25.0 (95% CI: 17.3-30.5) months, and BCLC C 15.0 (95% CI: 13.8-17.7) months. In CP B patients, median OS for BCLC A was 27 (95% CI: 21-30.2) months, BCLC B 15.0 (95% CI: 12.3-19.0) months, and BCLC C 8.0 (95% CI: 6.8-9.5) months. A total of 49 (5%) and 110 (11%) patients developed grade 3/4 albumin and bilirubin toxicities, respectively.

4.2 Ongoing TheraSphere Clinical Program in HCC

Currently, one phase III randomised clinical study prospectively investigating the use of TheraSphere in HCC has completed enrollment with 526 patients randomised, and patients currently in follow-up. This study will provide level one evidence on the effectiveness and safety of TheraSphere in combination with sorafenib for solitary or multinodular, unilobar or bilobar HCC patients with or without PVT compared to sorafenib alone. This clinical study will be used to support Pre-Market Approval (PMA) status for TheraSphere in the U.S. The study is:

- STOP-HCC – Phase III Clinical Study of Intra-arterial TheraSphere® in the **T**reatment **o**f **P**atients with Unresectable Hepatocellular Carcinoma (**HCC**). (TS-103 NCT01556490)

4.3 Disease Background and Clinical Summary of the STudy Device Experience of mCRC

Approximately 50% of colorectal cancer deaths are due to liver metastases (Bengtsson et al 1981). The management of colorectal cancer liver metastases (CLMs) continues to evolve, and long-term survival is now not only a distinct possibility but also a reality for many patients (Van den Eynde, 2009, Chiappa 2009). Systemic chemotherapy without surgical resection is generally non-curative and requires continuous therapy. This treatment approach alone rarely yields long-term survivors with a 2-year survival rate of approximately 40% (Ksienski et al, 2010). Standard treatment for these patients involves chemotherapy based on fluoropyrimidines, oxaliplatin, and irinotecan (used in combination and sequentially); and monoclonal antibodies targeting vascular endothelial growth factor (VEGF; bevacizumab). In patients with KRAS wild-type tumours, monoclonal antibodies targeting epidermal growth factor receptor (EGFR; cetuximab and panitumumab) are also used (Van Cutsem et al, 2015). Additional options are available for patients that maintain good performance status and are putative candidates for further therapy. Regorafenib was the first small-molecule multikinase inhibitor with survival benefits in metastatic colorectal cancer which has progressed after all standard therapies. Median overall survival was 6·4 months in the regorafenib group versus 5·0 months in the placebo group (hazard ratio 0·77; 95% CI 0·64–0·94; one-sided p=0·0052) (Grothey A et al 2013). TAS-102 is an orally administered combination of a thymidine-based nucleic acid analogue trifluridine, and a thymidine phosphorylase inhibitor, tipiracil hydrochloride. Trifluridine is the active cytotoxic component of TAS-102; its triphosphate form is incorporated into DNA, with such incorporation appearing to result in its anti-tumour effects. Tipiracil hydrochloride is a potent inhibitor of thymidine phosphorylase and, when combined with trifluridine to form TAS-102, prevents the rapid degradation of the trifluridine, allowing for the maintenance of adequate plasma levels of the active drug, The RECOURSE trial demonstrate the benefit of TAS102 versus placebo in patient with mCRC. The median overall survival improved from 5.3 months with placebo to 7.1 months with TAS-102, and the hazard ratio for death

in the TAS-102 group versus the placebo was 0.68 (95% confidence interval [CI], 0.58 to 0.81; P<0.001) (Mayer R et al 2015). The Metastatic colorectal cancer liver metastases Outcomes after RadioEmbolization (MORE) study (Hickey et al 2016, Kennedy A et al, 2017) was a retrospective analysis of 606 patients with unresectable colorectal liver metastases treated with SIRT. The first analysis of this study was completed with a last patient follow-up of 77.7 months. SIRT treatment with Y-90 was considered for patients with advanced liver-only or liver-dominant metastatic colorectal cancer which was deemed not suitable for surgery, ablation, or systemic therapy, and which had progressed or become refractory to at least one line of systemic therapy. Dates of death were obtained for 574 out of a total of 606 patients, and overall survival (OS) data analyzed. Updated median OS was 10.0 months (95% CI: 9.2-11.8 months) at a median follow-up of 9.5 months. Patients received a median (range) of 2 (0 to 6) lines of chemotherapy. Baseline characteristics and factors significantly associated with patient survival (P<0.01) include poor ECOG performance status, markers of advanced disease such as increased extent of tumour-to-target liver involvement, poor baseline liver function, pre-treatment anemia, lung shunt fraction, and number of lines of prior chemotherapy. Patient age did not significantly affect survival outcomes. Another 531 patients study has evaluated SIRT for colorectal liver metastases. The most common clinical adverse events were fatigue (55%), abdominal pain (34%), and nausea (19%). Grade 3 or 4 hyperbilirubinemia occurred in 13% of patients at any time. The median overall survival from the first Y-90 treatment was 10.6 mo (95% confidence interval, 8.8–12.4). Performance status, no more than 25% tumour burden, no extrahepatic metastases, albumin greater than 3 g/dL, and receipt of no more than 2 chemotherapeutic agents independently predicted better survival outcomes. Conclusion: This multiinstitutional review of a large cohort of patients with colorectal liver metastases treated with Y-90 radioembolisation using resin microspheres has demonstrated promising survival outcomes with low toxicity and low side effects. The outcomes were reproducible and consistent with prior reports of radioembolisation. Therefore, there is growing evidence, to support of Y-90 for treatment of colorectal liver metastases. Collaboration among oncologists and interventional radiologists is needed to more precisely define the role of Y-90 radioembolisation in the treatment of colorectal liver metastases. This registry will be key to get a lot of information from multiple sites across a large territory (Kennedy et al 2019).

4.4 Ongoing TheraSphere Clinical Program IN mCRC

Currently, one phase III randomised clinical study prospectively investigating the use of TheraSphere in mCRC has completed enrollment with 428 patients randomised, and patients are currently in follow-up. This study will provide level one evidence on the effectiveness and safety of TheraSphere in second line treatment and combination with systemic chemotherapy for patients liver metastases from colorectal cancer, compared to systemic chemotherapy alone. This clinical study will be used to support Pre-Market Approval (PMA) status for TheraSphere in the U.S. The study is:
EPOCH: TheraSphere Yttrium-90 Glass Microspheres in Second-line Treatment of Patients With Metastatic Colorectal Carcinoma of the Liver: Protocol for the EPOCH Phase 3 Randomized Clinical Trial (NCT01483027).

4.5 Disease Background and Clinical Summary of the Study Device Experience in iCC

Cholangiocarcinoma (CC) is a primary liver cancer with features of cholangiocyte differentiation, the epithelial cell lining the intra and extrahepatic portions of the biliary tree. Intrahepatic cholangiocarcinomas (iCC) are located within the hepatic parenchyma. The second-order bile ducts serve as the point of separation between iCC and extrahepatic CC (eCC), which is divided into perihilar CCs (pCCs) and distal CCs (dCCs). In a large series of patients with bile duct cancer, 8% had iCCA, 50% had pCC, and 42% had dCC. Each subtype has distinct clinical feature, risk factors, molecular pathogenesis, therapeutic options, and prognosis. Classically, CC has been always regarded as a 'rare' tumour, at least in Western countries, but over the last 15 years, its incidence has steadily increased worldwide, and nowadays, it represents the second most common type of primary malignancy in the liver (15%-20% of cases) after hepatocellular carcinoma.(Pellino A et al, 2018; Rivzi S et al, 2013) CC is an aggressive malignancy with a poor overall prognosis and median survival of less than 2 years in patients with advanced disease. Potentially curative surgical treatment options are limited to the small subset of patients with early stage disease. Presently, the available systemic medical therapies for advanced or metastatic CC have limited therapeutic efficacy. (Rivzi S et al, 2017; GBD, 2015).

Gemcitabine plus cisplatin remains the standard first-line systemic therapy for advanced cholangiocarcinoma and offers a median survival of approximately 1 year. No standard regimens beyond the first line and no targeted or immunotherapy agents are approved yet in this disease. Development of molecular targeted therapy in this heterogenous and relatively rare malignancy continues to be a challenging area , (Valle J et al 2010, Lamarca A et al 2020). Transarterial radioembolisation with yttrium-90 (Y-90) microspheres has been included in the armamentarium of treatment options for iCC. Published studies have reported a wide range of survival outcomes after radioembolisation treatment (6.1–22 months), which is likely due in part to the small number of patients (18–46 patients) included in those studies and the heterogeneity of selection criteria in the different protocols (9–19). A recent Retrospective review of 85 consecutive patients demonstrated a Median overall survival (OS) from diagnosis was 21.4 months (95% confidence interval [CI]: 16.6–28.4); median OS from radioembolisation was 12.0 months (95% CI: 8.0–15.2). Patients with solitary tumours had significantly longer median OS from radioembolisation than patients with multifocal disease (25 vs. 6.1 months, P=0.006) (Gangi A et al 2018). Also, a phase 2 clinical trial, in Cholangiocarcinoma (MISPHEC) trial (Edeline J et al, 2019), included patients with unresectable iCC who have never received chemotherapy or intra-arterial therapy treated at 7 centers, patient received TheraSphere in association with chemotherapy (GEMCIS). Primary efficacy end point was response rate at 3 months. Secondary end points were toxic effects, progression-free survival, overall survival, disease control rate, and response rate according to Choi criteria. The study reached the end points for 41 patients included in the study, response rate according to RECIST was 39% (90% CI, 26%-53%) at 3 months according to local review and was confirmed at 41% as best response by central review; disease control rate was 98%. According to Choi criteria, the response rate was 93%. After a median follow-up of 36 months (95% CI, 26-52 months), median progression-free survival was 14 months (95% CI, 8-17 months). Median overall survival was 22 months (95% CI, 14-52 months). Of 41 patients, 29 (71%) had grades 3 to 4 toxic effects; 9 patients (22%) could be downstaged to surgical intervention, with 8 (20%) achieving R0 (microscopic-free margins) surgical resection. After a median of 46 months (95%CI, 31 months to not reached) after surgery, median relapse-free survival was not reached among patients who underwent resection.

These data support the therapeutic role of radioembolisation for the treatment of unresectable iCC with good efficacy, and a high rate of downstaging to resection in patient with unilobar disease and in association with chemotherapy. The safety profile was manageable, however patients with cirrhosis had more AEs than the patients without associated cirrhosis.

4.6 Ongoing TheraSphere Clinical Program in iCC

Currently, there is no randomised clinical study prospectively investigating the use of TheraSphere in iCC

4.7 General Description of Study Device

TheraSphere is a radioembolic therapeutic device used in the treatment of liver cancers. This device delivers locoregional radiation via insoluble glass microspheres where Y-90 is an integral constituent of the glass matrix. The mean sphere diameter ranges from 20 to 30 µm. Each milligram contains between 22,000 and 73,000 microspheres. TheraSphere is supplied in 0.6 mL of sterile, pyrogen-free water contained in a 1.0 mL vee-bottom vial secured within a 12 mm clear acrylic vial shield. TheraSphere is available in custom dose sizes in increments of 0.5 GBq between 3 GBq and 20 GBq.

The microspheres are delivered to the liver tumour through a catheter placed into hepatic artery that supplies blood to the tumour. The microspheres, unable to pass through the vasculature of the liver due to arteriolar capillary blockade, are trapped in the tumour and exert a local radiotherapeutic effect with some concurrent damage to the surrounding normal liver tissue (TheraSphere™ Y-90 Y^{90} Glass Microspheres Instruction for Use (IFU)).

In the U.S., TheraSphere is available as a Humanitarian Use Device (HUD) available to patients through the Humanitarian Device Exemption (HDE) provision. TheraSphere is approved under this program for use in radiation treatment or as a neoadjuvant to surgery or transplantation in patients with unresectable HCC who can have placement of appropriately positioned hepatic arterial catheters. The device is also indicated for HCC patients with partial or branch PVT/occlusion, when clinical evaluation warrants the treatment.

In the European Union, TheraSphere is approved for the treatment of hepatic neoplasia.

4.8 Rationale for the post Registration study

On 20 February 2018, the Haute Autorité de Santé-Commission Nationale d'evaluations des dispositifs médicaux et des Technologies de Santé (CNEDiMTS) approved the reimbursement of TheraSphere for the treatment of HCC in France for five years (until 31 Dec 2023).

On 18 February 2020, the CNEDiMTS approved the reimbursment of TheraSphere for the treatment of mCRC and iCC in France until 31 Dec 2023.

A post registration study was requested to collect survival and safety data on all patients treated with TheraSphere over this initial five year period. Reimbursement is available to those patients for whom the therapeutic alternatives available are limited and a therapeutic interest in TheraSphere is relevant.

4.9 RATIONALE FOR THE STUDY DESIGN AND RELEVANCY OF DATA COLLECTED.

To document the safety, efficacy and QoL of patients treated with TheraSphere in a real life setting, HAS-CNEDiMTS has requested a follow-up of the targeted population under a registry study.

Biocompatibles UK Ltd will conduct this post registration study to provide valuable information on the management of HCC, mCRC and iCC patients treated with TheraSphere in a real world clinical practice setting in France.

The study will be designed in order to capture:

- The diversity of patient selection and treatment decision
- Disease presentation
- Treatment procedures
- Treatment effectiveness including, but not restricted to, survival
- Safety
- QoL
- Dosimetry parameters that are associated with treatment efficacy & safety

Patient and tumour characteristics at baseline and treatment physician/patient expectations, are important treatment decision factors and need to be assessed.

Regarding treatment procedure, this registry is a unique opportunity to provide guidance and training to the French hospitals involved in the treatment of patients with TheraSphere in order to achieve excellence in treatment delivery. It has been demonstrated in HCC (retrospectively, and in numerous publications) that multi compartment dosimetry treatment is the best way to optimise treatment outcomes.

The assessment of QoL broadens the evaluation of medical treatments assessing factors other than those bio-medically related. This is particularly important when the disease prognosis is poor or when alternative treatments have equivalent effectiveness but different tolerability. Information collected from the QoL questionnaires will help in treatment decision-making. They are also useful tools in identifying supportive care needs and improving the global care of cancer patients. QoL is both a clinically and physiologically meaningful endpoint and is best defined from the patient's perspective.

The results from this registry could potentially create a place for TheraSphere in the BCLC treatment algorithm of HCC, and in the European and U.S. guidelines for mCRC and iCC and prompt research into its use in alternative treatment indications.

4.10 DOSIMETRY

Radionucleide therapy is relevant for hypervascular liver cancers and this approach allows for selective irradiation of tumours while maximally preserving the normal tissue hepatic parenchyma. External beam radiation therapy and radioembolisation share the same therapeutic principles.

There are two types of radiobiological ionising radiation:

- Cells death and tissue necrosis that occur only after exceeding an absorbed dose threshold that varies according to the organs. Once this threshold is reached, the radiobiological effect occurs with a proportionality between the dose and the percentage of cell death.
- Random effects which correspond to the risk of carcinogen and disease which may be secondary to irradiation. These occur randomly and do not respond to the notion of threshold dose.

The careful planning of a radiation treatment is required to be able to deliver treatment above the threshold dose and below a toxic dose of a radiotherapy. It is mandatory to know the absorbed dose to tumours and the dose absorbed by the critical organs.

If these parameters are relatively easy to identify in external radiotherapy, they are much more difficult to calculate in the case of radionucleide therapy, in particular because of possible biological elimination of the product used. Moreover, the biological effects of external radiotherapy or radionucleide therapy on the tissues are different because a heterogeneous dose distribution in the case of radionucleide therapy, additionally a much lower dose rate is required compared to external radiotherapy to achieve the same absorbed dose.

The IFU recommendation for TheraSphere is to deliver an absorbed dose of 80 to 150 Gy to the liver without exceeding 50 Gy cumulatively in the lungs.

$$Dose\ (Gy) = \frac{50[Injected\ Activity\ (GBq)][1-F]}{Liver\ Mass\ (kg)}$$ (where F= lung fraction)

Liver absorbed dose determination based on the above formula has been utilised to treat >19,000 patients worldwide and has exhibited acceptable safety and tolerability, as well as clinical benefit in treated patients.

Ho et al., 1996, proposed a partition method that separately accounts for the tumour and normal tissue absorbed dose and applies the medical internal radiation dose (MIRD) schema. The partition model assumes that Y-90 microspheres (physical properties) distribute similarly to ^{99m}Tc-MAA (physical properties).

The correlation of ^{99m}Tc-MAA and Y-90 microspheres has been debated in the literature but there is sufficient evidence to suggest that the correlation is clinically useful including a reasonable correlation between absorbed dose estimated from ^{99m}Tc-MAA scintigraphy and direct intra-operative measurements of dose (correlation coefficient = 0.862 for tumour (Knesaurek et al., 2010)). The correlation is more robust in HCC than in other liver tumours and when the catheter location is similar for ^{99m}Tc-MAA and Y-90 microspheres. In a study conducted by Chiesa, (Chiesa et al., 2011) correlation of ^{99m}Tc-MAA particles relative to TheraSphere ranged from 71-100%, with the majority of discordance attributed to intentional differences in catheter placement.

Further, from the pioneering work by Ho et al. (Ho et al., 1996) who have used scintigraphy (2D), scintigraphy 3D absorbed dose estimates have been used to evaluate pretreatment ^{99m}Tc-MAA SPECT or SPECT/CT in a similar manner and post-treatment Y-90 imaging measurement using Bremsstrahlung and/or Positron Emission Tomography (PET) Y-90 imaging.

Both post-treatment Bremsstrahlung and PET Y-90 imaging provide correlative data with ^{99m}Tc MAA, although PET Y-90 has superior resolution. Importantly, absorbed dose estimates with ^{99m}Tc-MAA have demonstrated correlation with Adverse Event (AE) incidence, tumour response, tumour complete pathological necrosis and OS (Walrand S et al., 2014). The goal of advanced dosimetry is to facilitate improved tolerability by providing pre-therapy information to allow the physician to limit absorbed dose to normal liver parenchyma and/or to increase the absorbed dose to the tumour while maintaining a safe dose to normal liver. In this manner, patient selection and optimal dose selection can minimise AEs and increase tumour response.

A number of retrospective single institution studies have demonstrated the benefits of dosimetry calculations based on ^{99m}Tc-MAA as well as the benefits of post-administration calculation of Y-90 absorbed dose. Standardisation of dosimetry techniques is required to better determine the optimal tumour and normal liver parenchyma dose.

For HCC, the current threshold absorbed dose recommendations in the scientific literature for TheraSphere varies from 205 to 500 Gy to the tumour using a two compartment model. In these studies, the image acquisition, reconstruction, region segmentation and dose calculation methods all vary, and likely contributes to the great variability in the recommendations.

Several studies have confirmed this notion of dose-response in patients treated with Y-90-labeled glass microspheres. In particular, it has been demonstrated, using a simple dosimetric method, that an absorbed threshold of 205Gy was associated with achievement of a tumour response; (Garin et al., 2013; Strigari et al., 2014), also the recent results of the DOSISPHERE-01 trial (Garin et al 2020) has confirmed in a small randomised study that personalised dosimetry that delivers a threshold dose of 205 Gy to the tumour resulted in a significant increase in response rate and survival compared to a standard dose of 120 Gy to the perfused liver.

Thus, in the preliminary study of 36 patients (Garin et al., 2013), none of the 8 patients who presented tumour absorbed dose less than 205 Gy responded to treatment, while 89% of patients who received a tumour absorbed dose of at least 205 Gy responded. These results were confirmed by the addition of a cohort of 71 patients, including 17 patients exposed to intensification of treatment, with the aim of exceeding a tumour absorbed dose of 205 Gy without an increase in hepatic toxicity (5.8% for intensified patients vs. 9.2% for non “boosted” treatment).

The two-compartment dosing method, coupled with the determination of pre-treatment MAA of tumour dose and dose to the perfused healthy liver, have been shown to be reliable in patients that received a lobar treatment. Therefore, it is proposed that patients scheduled to receive TheraSphere treatment, should benefit from a multicompartment dosimetry approach, which will optimise tumour response while minimising treatment risk. Sites that are not experienced in dosimetry assessments, will receive expert training and guidance.

The novel dosimetry tool (Simplicit^{90}Y™, Mirada) utilised to ascertain the tumour absorbed dose, non-tumour tissue absorbed dose and total liver absorbed dose, could be provided free of charge for the duration of the current study, however, other software is available and could be used.

An international multidisciplinary working group has reviewed existing data and addressed gaps in knowledge related to dosimetry and has provided dosimetric recommendations (Salem R et al, 2019).

The goal of these recommendations is to optimise glass microspheres radiation therapy for HCC while accounting for variables including disease presentation, tumour vascularity, liver function, and curative/palliative intent. The recommendations aim to unify glass microsphere users behind standardised dosimetry methodology that is simple, reproducible and supported by clinical data, with the overarching goal of improving clinical outcomes and advancing the knowledge of dosimetry.

Also, while the relationship of dose and response has been widely demonstrated, there is less evidence for a threshold safety dose to normal liver when the treatment planned is at least a lobar treatment. It is suggested that the underlying liver function and the hepatic reserve play a major role. Targeting up to 75 Gy absorbed dose to the entire healthy tissue has been proposed (consensus recommendation).

Regarding a threshold safety dose to normal liver when the treatment planned is at least a lobar treatment. It is suggested that the underlying liver function and the hepatic reserve play a major role.

- Factors significantly associated with increased toxicities (>15% of liver decompensation)
    - For the PVT patients, poor MAA PVT targeting
    - Normal perfused tissue absorbed dose of ≥120 Gy and a hepatic reserve <30%.
      Or
    - Average absorbed dose to the whole non tumoural liver of 50 Gy if bilirubin > 1.1 mg/dL
      Or
    - Average absorbed dose to the whole non tumoural liver of 90 Gy if bilirubin ≤1.1 mg/dL

Chiesa et al. (EJNM 2020) recently published a study that aimed to determine a safety limit through a retrospective analysis of ^{99m}Tc-MAA based pre-treatment dosimetry. Patients that received lobar, or bilobar TheraSphere administration were evaluated for liver decompensation (LD), defined as the occurrence, within 6 months from treatment, of any of the following six features: total bilirubin level >3 g/dL, prothrombin time INR >2.2, clinically detectable ascites, encephalopathy, oesophageal varices bleeding and death, which was added to this composite definition of toxicity. The absorbed dose averaged over the whole normal tissue liver (including the non-injected lobe) was a prognostic indicator correlated with liver decompensation (odds ratio = 4.24). Basal bilirubin >1.1 mg/dL was a second even more significant risk factor (odds ratio = 6.35). Normal Tissue complication probability analysis, stratified with this bilirubin cut-off, determined a 15% liver decompensation risk at 50 Gy / 90 Gy for bilirubin >/< 1.1 mg/dL. These results are valid for a Y-90 glass microsphere administration 4 days after the reference time. They proposed a threshold absorbed dose averaged over the whole non tumoural liver of 50 Gy for basal bilirubin > 1.1 mg/dL and of 90 Gy for basal bilirubin ≤1.1 mg/dL.

For mCRC there is no consensus dose recommendation to tumour/normal liver. In the BTG sponsored trial EPOCH, the dosimetry objective was 120 Gy +/- 10% (Chauhan et al 2019). TheraSphere was evaluated in a cohort of seventy-two patients with unresectable hepatic colorectal metastases, who were treated at a targeted absorbed dose of 120 Gy with a median delivered dose of 118 Gy. Treatment-related toxicities included fatigue (61%), nausea (21%), and abdominal pain (25%) with Grade 3 and 4 bilirubin toxicities observed in 9 of 72 patients (12.6%) (Mulcahy et al 2009). In a recent

trial, where 23 patients were treated with SirSphere (Abott et al 2020), a tumour-by-tumour dosimetric analysis, following SIRT in patients with mCRC, a dose-response relationship was explored. The mean radiation absorbed tumour dose of 35.5±9.4 Gy and mean normal liver dose of 26.4±6.8 Gy. Threshold mean, median and DVH 70 (Dose in 70% of the volume) doses for response were 48.3, 48.8, and 41.8 Gy respectively. In another publication on 133 patients, Van den Hoven (JNM 2016) has demonstrated, in patients treated with SirSphere, that a median average tumour dose of 40-60 Gy led to a 50% metabolic response and that there was a trend for improved OS for tumour dose > 60 Gy.

For iCC, there is no tumour/non tumour dose recommendation to tumour/normal liver. In the MISPHEC trial (Edeline J et al Jama Oncol 2019), the median dose delivered to the tumour was 317 Gy (range, 64-1673 Gy), and the median dose delivered to the non tumour liver was 87 Gy (range,4-235 Gy). The SIRT doses recommended in this study were defined using manufacturer label instruction.
However, accumulating evidence suggest that the definition of an appropriate dose delivered to the tumour, rather than a generic dose delivered to the targeted liver, might improve results. In a small study Nezami et al, 2018 described a mean tumour dose of 205.7±19.7 with a ratio tumour/non tumour dose of 4.9±0.7, therefore ensuring a low dose to perfused normal tissue even with high tumour dose. A study from Manceau et al 2018, decribed the treatment of 40 non resectable iCC patients treated with Y-90 glass microspheres combined with chemotherapy. Tumour dose (TD), dose to perfused normal liver, and dose to perfused liver were calculated with Tc MAA SPECT/CT. Response, factors associated with response and toxicity were analysed. Mean TD was 322 ± 165Gy, mean dose to perfused normal liver was 74 ± 24Gy, mean dose to perfused liver was 128 ± 28Gy. Threshold dose associated with response was 158Gy. Mean dose to perfused non tumoural liver was not associated with liver toxicity but Child-Pugh score and underlying cirrhosis were associated with liver toxicity.

5.0 STUDY OBJECTIVES

The purpose of this registry study is to gather effectiveness, QoL and safety information on the current clinical use of TheraSphere in France for the renewal of the reimbursed indications.

5.1 The Primary objective

The primary purpose of this registry study is collect survival and QoL information on the current clinical use of TheraSphere in France.

5.2 The Secondary objectives:

- To assess safety
- To assess treatment expectation/goal and achievement of that expectation
- To describe the patient population and treatment
- To compare outcomes according to baseline patient, tumour characteristics and treatment procedure (dosimetry).

5.3 Study Design

Prospective, non-interventional, single arm, open label post registration, single territory, multi-centre study based in France.

Study Type: Recherche Impliquant la Personne Humaine de type 3 (RIPH type 3).

Patient characteristics and treatment data will be collected during the period from patient registration prior to TheraSphere administration until death, study withdrawal or study termination, whichever occurs first. This will allow the description of treatment procedure, the assessment of effectiveness and the identification of prognostic and predictive factors for outcomes patients receiving TheraSphere treatment for the reimbursed indications included in the registry.

The registry includes patients for which TheraSphere has been prescribed and reimbursed, and who have not opposed the collection of their data. The registry is non-interventional and therefore no assessments outside of local standard medical practice will be required except collection of QoL information.

Data generated during visits conducted as part of local standard medical practices will be included in this registry. As the standard of medical practice may differ between sites, data collected may vary.

It is anticipated that the registry will enroll >500 patients from approximately 30 sites.

5.4 Primary Outcome measures

Overall Survival (OS) combined with variation of QoL measurements before and after treatment.

QoL will be assessed by FACT-Hep questionnaire collected at baseline, first pre-TheraSphere treatment visit, and then at every follow-up visit until the patient opposes the further collection of their data, withdraws from the study registry, end of study date or death, whichever occurs first.

5.5 Secondary Outcomes measures

1. SAEs graded using the National Cancer Institute-Common Terminology Criteria for Adverse Events version 5.0 (NCI-CTCAE v 5.0).
2. Grade 3 or higher adverse events (AEs) (graded using NCI-CTCAE v 5.0) related or possibly related to the device, or the device administration procedure, that occur up to 90 days after every TheraSphere administration or the first follow-up visit (if after 90 days).
3. Number and duration of re-hospitalisations related to TheraSphere treatment up to 30 days after treatment administration.
4. Description of treatment expectation (e.g. benefit to survival and disease control) before administration of TheraSphere and number of patients achieving treatment expectation.
5. Qualitative tumour response assessment (Index lesion response and overall response): number of patients having complete response (CR), partial response (PR), stable disease (SD), progressive disease (PD).
6. Target tumour marker response defined as a ≥50% decrease in:
    a. AFP levels for patients with a baseline AFP level ≥200 ng/mL).
    b. CA 19-9 levels for patients with a baseline CA 19-9 level ≥ twice the upper limit of normal.
    c. CEA levels for patients with a baseline CEA level ≥ twice the upper limit of normal.
7. Number of patients receiving a post TheraSphere anti-cancer treatment, including surgery.

8. Number of patients receiving a post TheraSphere best supportive care treatment.
9. Description of vascular access (radial/femoral) used to administer TheraSphere.

5.6 Dosimetry Outcomes measures

1. Association between tumour(s) location at baseline and location of lesions targeted by Technetium-99m Macroaggregated albumin (^{99m}Tc-MAA) (Single Photon Emission Computed Tomography SPECT or SPECT/CT*).
2. Association between tumour(s) location at baseline and location of lesions targeted by Y-90 (Positron Emission Tomography (PET)/CT, Y-90 PET/MRI or Y-90 SPECT/CT*).
3. Association between tumour(s) location based on ^{99m}Tc-MAA (SPECT or SPECT/CT), and location of tumour targeted by Y-90 using post-treatment (PET/CT, PET/MRI or SPECT/CT)*.
4. Association between PVT at baseline and PVT targeted by ^{99m}Tc-MAA (SPECT or SPECT/CT), Y-90 (PET/CT or PET/MRI or SPECT/CT).
5. Association between tumour and normal tissue liver absorbed doses, determined with ^{99m}Tc-MAA (SPECT or SPECT/CT), with qualitative tumour response (CR or PR), OS and safety, respectively.
6. Association between tumour and normal tissue liver absorbed doses, determined with Y-90 (PET/CT or PET/MRI), with qualitative tumour response (CR or PR), OS and safety, respectively.
7. Association between tumour and normal tissue liver absorbed doses determined with ^{99m}Tc-MAA (SPECT or SPECT/CT) and with Y-90 (PET/CT or PET/MRI).
8. Determination of Dose volume histogram (DVH) for total perfused tumour, Index lesion and whole normal liver tissue, using ^{99m}Tc-MAA (SPECT or SPECT/CT) and Y-90 (PET/CT or PET/MRI).

* A score that describes the intensity of distribution of ^{99m}Tc-MAA / Y-90 in tumour versus normal tissue, and the tumour coverage with ^{99m}Tc-MAA / Y-90 in tumour will be created (Appendix 5)

Dosimetry measures will be assessed locally by investigator for all patients and centrally for HCC and iCC patients. Images used for dosimetry assessements will be uploaded to a central imaging database and reviewed independently.

6.0 PATIENT SELECTION

6.1 Patient Population

All patients for whom treatment with TheraSphere has been prescribed and reimbursed in France will be eligible for this study.

It is estimated that data from >500 patients will be entered into this registry from approximately 30 sites in France.

6.2 Indications and Conditions for TheraSphere Reimbursement

Indications for reimbursement

Patients with HCC who meet the following criteria:
1. Confirmed HCC, by histology or America Association for the Study of Liver Diseases (AASLD) or EASL imaging criteria

2. Patient scheduled to receive TheraSphere treatment per MTB decision
3. Treatment given as a palliative intent (patient not eligible* for resection or ablation)
4. Patient who is BCLC B or BCLC C or with PVT** (Appendices 1 and 3)
5. Patient who is not eligible* for, or has failed sorafenib treatment
6. Good general status (ECOG score 0 or 1) (Appendix 2)
7. Patient with a preserved liver function*** (Child Pugh A-B) (Appendix 3)

*Treatment not possible or not recommended
** Portal vein invasion by tumour
***Preserved liver function: includes patients with different degrees of liver functional reserve (non treated liver) that has to be carefully evaluated. Compensated liver disease (without ascites) is required to obtain optimal outcomes.
(Forner et al., 2018; EASL Guidelines 2018)

Patients with mCRC who meet the following criteria:

1. Patient scheduled to receive TherasSphere treatment per MTB decision
2. Preserved general health condition (ECOG score ≤ 2)
3. Hepatic tumour load (<25%)
4. Absence of extrahepatic disease
5. Refractory or intolerant to all approved intra venous and oral therapies for colorectal cancer. Progression under chemotherapy should be documented.

Patients with iCC who meet the following criteria:

1. Patient scheduled to receive TherasSphere treatment per MTB decision
2. First line palliative treatment for iCC
3. Patient unresectable at diagnosis or in a recurrence after resection
4. With or without association with chemotherapy
5. Preserved general health condition ECOG ≤ 1) when treated with TheraSphere in combination with concomitant chemotherapy
6. Preserved general health condition (ECOG score ≤ 2) when treated with TheraSphere alone
7. Absence of extrahepatic disease
8. Hepatic tumour load <50%
9. Patient with preserved liver function (Child-Pugh score A or B in case of cirrhosis).

Conditions for TheraSphere use

- The use of TheraSphere will be carried out in accordance with the decree number 2007-389 dated 21 March 2007 relative to the technical operating conditions applicable to cancer care activity.
- TheraSphere is used by multidisciplinary teams that include: a nuclear physician, an interventional radiologist with the expertise of hepatic embolisation in oncology, a radiophysicist and a radiopharmacist. This activity must be carried out in centres with sufficient infrastructure to be authorised by the French Nuclear Safety Authority (Agence de Sécurité Nucléaire - ASN) to carry out internal radiation activities.
- The decision to perform the treatment and the post-treatment follow-up has been taken under patient agreement and after a positive treatment recommendation of the local/regional MTB specialised in HCC, mCRC and iCC. The MTB must include at least: an oncologist, a hepatologist, or a hepatologist skilled in oncology, a surgeon specialist in liver surgery, an interventional radiologist, a nuclear medicine specialist, a radiation oncologist, and a palliative care specialist.

- The patient must have access to a supportive care team.

6.3 Contraindications for TheraSphere treatment

The use of TheraSphere is **contraindicated** in patients who:

1. ^{99m}Tc-MAA SPECT/CT or SPECT shows any deposition to gastro intestinal tract that could not be corrected by angiography techniques.
2. ^{99m}Tc-MAA SPECT/CT or SPECT show shunting to blood to the lung that could result in delivery of greater than 16.5 mCi of yttrium-90 to the lungs, 30Gy in a single treatment and 50Gy in cumulative treatments
3. hepatic artery catheterisation is contraindicated (vascular abnormalities or bleeding diasthesis).
4. have severe hepatic dysfunction or pulmonary insufficiency
5. are pregnant

6.4 Warnings for TheraSphere Use

The physician should always take into consideration any pre-treatment risk factors (listed below) when making the decision to treat with TheraSphere:

1. Patient with bulky disease (measured tumour volume >70% or tumour nodules too numerous to count)
2. Patient with tumour volume > 50% combined with albumin < 30g/L.
3. Patient with infiltrative disease.
4. Patient with hepatic functional reserve (non treated liver) ≤30% of total liver volume/function and dose to the perfused normal tissue liver ≥120 Gy, determined by ^{99m}Tc-MAA (SPECT or SPECT/CT)
5. Patients with Portal Vein Thrombosis (PVT) type Vp4 with **complete** main portal vein invasion, whichever the ^{99m}Tc-MAA targeting.

   Note: Patients with incomplete main portal vein invasion and good ^{99m}Tc-MAA targeting can be considered.
6. Bilirubin >2 mg/dL or >34 µmol/L
7. AST/ALT >5 x ULN
8. Ascites > grade 1 under well conducted diuretic treatment (Appendix 7)
9. Child Pugh score > B7

6.5 Study duration

- **<u>Enrolment period:</u>** Five years – 01 Jan 2019 to 30 Dec 2023.
  - **Day One** is the date of publication of reimbursement of TheraSphere on the *liste de produits et prestations remboursables* (LPPR) list (01 Jan 2019).
  - **Date of enrollment close** will be five years after Day One (31 Dec 2023).

- **<u>Data collection duration</u>:**
  - **Start of Data Collection:** Data collection will start at each site following the site initiation visit (SIV) and site activation. The availability of reimbursement will occur before sites are activated. In such instances, to ensure the most complete data collection to meet HAS-

CNEDiMTS requirements, the data from those patients treated before the SIV date / site activation will be entered into the eCRF retrospectively.
    - **Date of last data collection:** latest 12 months follow-up visit available for the study (~31 Dec 2024).

- **<u>Duration of participation for patients:</u>**
    - Every patient will participate in the study from Visit 1, prior to initial TheraShere administration until end of study (31 Dec 2024), or death or date when the patient opposes to further participate in the study, whichever comes first.
- **<u>Data collection</u> periods (see schedule of Visits and Assessments in Section 3.0):**
    - **Initial 12 Month Period:** Clinical, biological, treatment, safety, QoL and imaging data will be collected from Visit 1, prior to initial TheraSphere administration, until the 12 month follow-up visit.
    - **Follow-Up period:** QoL, ECOG, safety data and survival status will be collected at at every standard of care follow-up visit until the end of the study:
    - **Data collection in the above study periods will be collected until:**
        - the patient opposes further data collection
        - the patient withdraws from the registry study (will not attend any further TheraSphere follow up visits), has started another cancer treatment, received best supportive palliative care, follow-up being no longer possible for any reason
        - the patient has died
    - At the end of the study (31 Dec 2024), survival status, including cause and date of death, and any subsequent treatment received since last TheraSphere treatment will be collected.

- <u>Study Duration</u>: 6 years
- <u>Final study report</u>: ~June 2025

7.0 TREATMENT AND FOLLOW UP OF PATIENTS

7.1 STUDY PRODUCT

Commercially available product at each site will be used to treat patients. Institutional labelling, storage, handling and accountability practices will apply.

7.2 OPPOSITION FOR DATA COLLECTION

Patients can oppose to have their data collected in this registry at any time, at such a time no further data will be collected in the eCRF. Data collected up to and including the date of opposition for further data collection will be included in the study.

7.3 PRE-TREATMENT PROCEDURES

All pre-treatment procedures, the ^{99m}Tc-MAA dosage selected and the administration procedures, will be perfomed per site procedure.

7.4 THERASPHERE TREATMENT

All pre-treatment procedures, the dosage selected and the administration procedures for TheraSphere will be perfomed in accordance with the IFU.

- TheraSphere infusion can be selective (tumour feeding artery, liver segment or liver sector) or non-selective (right/left liver, whole liver).
- The treatment could be administered in multiple infusions to address vascular abnormalities and tumour distribution (see IFU).
- If two TheraSphere treatment sessions are required to complete tumour treatment, the liver part with the highest tumour burden should be scheduled for first treatment. Before the second treatment session, a second angiogram with ^{99m}Tc-MAA scan should be performed. A second treatment would typically take place 30-45 days after the treatment to the first lobe, provided the patient has tolerated the first treatment.

7.5 Post TheraSphere treatment

Post TheraSphere treatment management of the patient is as per institutional practice.

Post TheraSphere treatment data will be collected at routine follow-up visit. Where follow-up is performed outside of the study institution, all attempts should be made to collect this information from the patient's usual care institution. The Patient Information Sheet has a provision allowing the collection of this information.

7.6 AdditionalTreatment(s)

Since TheraSphere treatment protocol lies outside the remit of this data capture registry, the number of TheraSphere treatments is up to physician discretion. All treatments and associated information will be collected as guided in Section 3.0.

7.7 Final visit

Every attempt should be made to conduct a Final Visit, which should be completed 12 months after initial treatment with TheraSphere or when the data collection is no longer relevant or possible:

- If the patient receives further anti-cancer treatent for the HCC, mCRC and iCC (the type and start date of treatment will be collected).
- If the patient is no longer able to attend follow-up visits (best supportive care, follow-up in another institution, patient has moved)

In these situations, no other follow up data will be collected in the eCRF except patient survival status (dead/alive) at interim analysis timepoints and at date of study termination.

7.8 Lost to Follow-up patient

In case the patient is lost to follow up, the follow-up data will not be collected but the survival status will be collected. The PI will be asked to recontact the patient at least twice before the patient is deemed lost to follow-up.

7.9 Quality of Life Collection

QoL data will be collected at routine follow-up visits, during the initial 12 month period and follow-up period, for all patients until the the study closes on 31 Dec 2024, unless:

- the patient opposes to further data collection
- the patient withdraws from the registry study (will not attend any further TheraSphere follow-up visits), has started another cancer treatment, received palliative care or follow-up being no longer possible at the site for any reason
- the study has ended (31 Dec 2024)
- the patient has died

7.10 SURVIVAL

Survival status will be assessed until study end (31 Dec 2024). For a patient attending a different institution, or lost to follow up, the investigator will attempt to contact the physician or GP that is responsible for that patient. In the situation where the required information cannot be collected from the aforementioned approaches, the civil status office in the patient's place of birth will be contacted.

8.0 MEASUREMENTS AND EVALUATIONS

8.1 GENERAL CONSIDERATIONS

The registry does not require any extra visits, assessments or treatment regimes in addition to those performed as part of standard medical practice at each site, except the collection of QoL information.

All patient visits and treatments are performed independently of this registry and are executed at the discretion of the treating physician following local standard medical practice.

Data recorded as part of these visits, treatments and assessments will be captured in this registry in accordance with Section 3.0.

8.2 MULTI COMPARTMENT DOSIMETRY ASSESSMENT

Where possible, a multi compartment dosimetry assessment should be performed, using dosimetry software (e.g. Simplicit^{90}Y™ software or equivalent) to calculate the absorbed dose in tumours and in normal tissue.

If required, the Simplicit^{90}Y™ software will be provided free of charge by Biocompatibles UK Ltd. A clinical license can be provided under a material loan agreement for the duration of the registry.

For sites who have no expertise or low experience of dosimetry, training can be provided by Biocompatibles UK. Ltd.

The upload of baseline imaging (CT/MRI), pretreatment ^{99m}Tc-MAA SPECT or SPECT/CT, post-treatment Y-90 SPECT/CT or Y-90 PET CT/MRI will be organised for all patients for central review for HCC and iCC patients only.

The lung shunt will be calculated on the **planar acquisitions** (geometric mean if possible) on pre treatment images. **Theses images will not be uploaded for central review.**

8.3 LIVER VOLUME ASSESSMENT, VOLUMES OF INTEREST

8.3.1 Volumes of Interest (VOI)

Definition

Volumes of interest (VOI) are: perfused liver, perfused tumours, normal tissue perfused liver, whole liver normal tissue, whole liver and remnant liver (non perfused liver). They are determined by contouring of images with using Simplicit^{90}Y™or equivalent software.

Determination of VOI

- Whole liver, all tumour volume and remnant liver volume (non perfused liver-tumour volume within the non perfused liver) are assessed on anatomical images (e.g. pre-treatment CTor MRI)

- Perfused liver, perfused tumours, normal tissue perfused liver, whole liver normal tissue volumes, can be assessed using ^{99m}TC-MAA SPECT/CT and/or baseline CT/MRI.

Guidance on the method for determination of VOI and dosimetry calculation will be provided in a work instruction.

8.3.2 Multi Compartment Pre treatment dosimetry

^{99m}TC-MAA imaging

- SPECT images should be reconstructed with attenuation and scatter correction methods when possible.

  - A qualitative and a quantitative analysis of the ^{99m}Tc-MAA deposition on VOI will be carried out with volumetric software analysis (Simplicit^{90}Y™ software or equivalent).

  - The software will perform dosimetric analysis in the VOI defined above.

  - The planned absorbed dose can be calculated:
    - In the perfused treated liver volume (e.g. segment, sector, lobe)
    - In the tumour(s)
    - And deduced for the normal tissue perfused liver, whole liver normal tissue and the whole liver

  - The activity to be administered will be calculated to deliver an absorbed dose, in agreement with the dosimetric objectives of the IFU, considering that 1 GBq of Y-90 delivers 50Gy to a lesion of 1 kg from the following recognised formula:

    A = D (Gy) x W

$$(1-S) \times 50$$

D = absorbed dose to the perfused volume(Gy)
A = activity injected (GBq)
S = pulmonary shunt
W = weight of the volume of perfused liver

- The weight of the liver volume considered is calculated with the following formula:

  Weight = Volume x 1.03

- Tumour and non-tumour liver planned absorbed dose is calculated as follows:

$$D = \frac{A \times (1-S) . 50}{W}$$

Applied to tumour and non-tumour liver parameters (volume and activity) provided by the quantitative analysis of SPECT/CT (Simplicit^{90}Y™ software or equivalent software).

- The recommended dose to the liver is between 80 Gy to 150 Gy (8000 rad to 15000 rad).
- The lung shunt will be calculated on the planar acquisitions (geometric mean).
- The lung dose will be calculated from the following formula: D = A x S x 50 described previously, considering as a first approximation that each lung weighs 1kg. The lung absorbed dose will be determined and
- The recommended dose to the lung is < 30Gy for one treatment and 50 Gy for cumulative treatment.

8.3.3 Dose Recommendation

8.3.3.1 Dose Recommendation for HCC

For dose guidance the investigator should refer to the consensus article (Salem R et al, 2019) and to the DOSISPHERE-01 study results (Garin E et al 2020).

For PVT patients: Poor MAA PVT targeting is a negative factor of response and increases the risk of liver decompensation.

Unicompartment dosimetry:

A future remnant tumour volume (FLRV) ≥ 30% is recommended.

Unique Selective treatment (ablative SIRT):

- Absorbed dose target of 400 Gy (range: 200 Gy – 800 Gy) to the perfused liver volume, where TheraSphere is infused to ≤ 2 liver segments.

Lobar treatment:

- Lobar volume infusion: absorbed dose range of 100 Gy – 150 Gy

Multi-compartment dosimetry:

1. **Tumour Dose**

<u>Lobar Administration</u>

- Target tumour absorbed dose >205 Gy with a goal of >250 Gy, where possible

<u>Selective treatment (ablative SIRT)</u>

- Selective infusion: absorbed dose target of 400 Gy (range: 200 Gy – 800 Gy) to the perfused liver volume, where TheraSphere is infused to ≤ 2 liver segments

One or more selective administration or the combination of lobar and selective administration, could be performed during the same or successive treatment session(s).

2. **Dose to normal tissue**

Dose to normal tissue must be controlled to limit the risk of liver decompensation, the following rules apply:

- Perfused normal tissue absorbed dose of ≤ 120 Gy with hepatic reserve ≥ 30%
  or
- All normal tissue absorbed dose of ≤ 90 Gy with bilirubin < 19 µmol/L,
  or
- All normal tissue absorbed dose of ≤ 50 Gy with a bilirubin ≥ 19 µmol/L

8.3.3.2 Dose Recommendation for mCRC

Multicompartment dosimetry is strongly recommended to determine the tumour and normal liver volume and absorbed dose considering that the patient might have received multiple and long term chemotherapy, therefore is at risk of liver complications.

Therefore an average dose of 120 Gy to the lobe is appropriate, and dose to all normal liver must be evaluated before treatment.

Risk factor for adverse events are:

- Tumour burden > 25%
- Long exposure to chemotherapy
- Albumin < 30g/L
- Elevated bilirubin
- Whole liver treatment

8.3.3.3 Dose Recommendation for iCC

Follow the same dose recommendation as HCC

8.3.4 Multi Compartment Post-Treatment Dosimetry

<u>Y-90 SPECT/CT or Y-90 PET/CT or Y-90 PET/MRI Imaging</u>

For local assessment: Dosimetry for perfused liver, tumours, normal tissue perfused liver and whole liver will be performed using Y-90 (PET/ CT or PET/MRI) imaging. If not available Y-90 SPECT/CT will be used, but only for qualitative analysis (assessments of Y-90 distribution in the tumour(s)), providing that the dosimetry assessed with SPECT/CT is not adequate (see section 8.6.5).

All 3D images must be reconstructed with attenuation and scatter correction methods

- A quantitative analysis of Y-90 deposition will be carried out with Volumetric Software Analysis (Simplicit^{90}Y™ software, or equivalent software) on PET

- VOIs can be determined by the methods described above (8.3.1)

- The software will perform dosimetric analysis in the VOI.

- The volume of Y-90 distribution, as well as the absorbed dose, can be calculated:
    - in the perfused treated liver volume
    - In the tumour(s) ,
    - and deduced for the non tumoural perfused liver, whole liver normal tissue and the whole liver

Data collected in the eCRF for dosimetry evaluation are described in Baseline Section 8.6.4.

8.4 Scientific Steering Committee (SSC)

The SSC will examine data summary tables during the interim analysis to ensure that the data collected will address the objectives as outlined within this registry. If the data collected will not adequately allow conclusions to be drawn, the committee may recommend a protocol amendment and/or revisions to the eCRF. SSC responsibilities and activities are fully detailed in the Steering Committee Charter.

8.5 Quality of life questionnaire

The patient will complete a paper questionnaire provided to them by the physician or study coordinator at baseline and during the follow-up visits.

The study coordinator/data entry person will enter the data from the paper questionnaire into the eCRF, which follows the same sequence as the paper questionnaire.

In the case where the patient is followed up in another centre, efforts to document the questionnaire should be done : the QoL questionnaire can be sent to their home with a "return" envelope. If required, the study coordinator will fill in the questionnaire during a telephone call with the patient, and document the answers on a paper questionnaire ready for data entry.

8.6 Data Collection Schedule and Assessments (see section 3.0)

8.6.1 Identification of patients eligible for data collection

Potential patients will be identified via the TheraSphere ordering system.

The CRA discuss the orders placed on the ordering system with the site and if they can be registered as a patient in the study.

Patient Identification

Each patient enrolled in the registry will be assigned a study identification number via the EDC system which will be used on any study documentation.

Figure 2: Patient Identification Flow

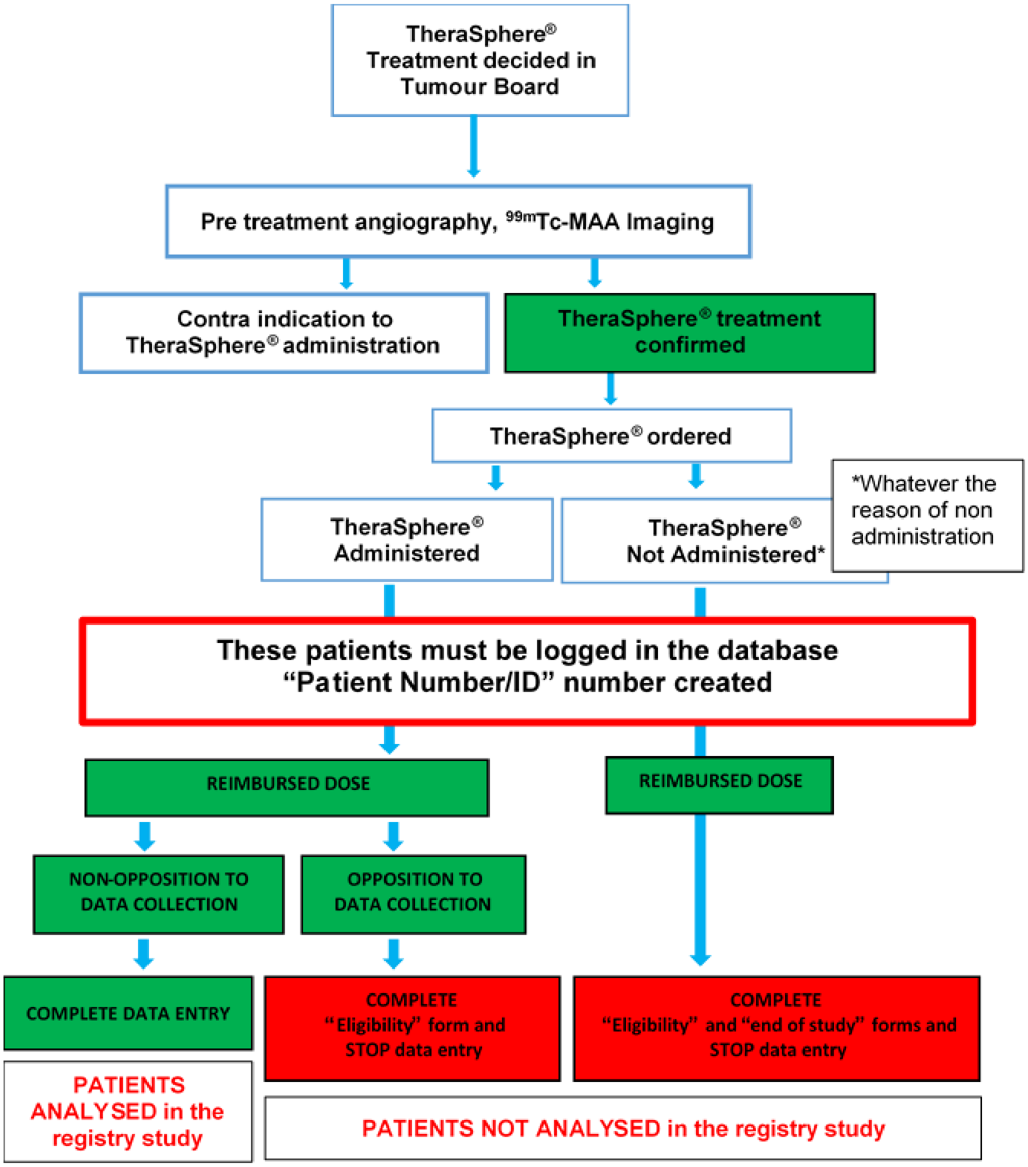

8.6.2 Patient Registration in the database (figure 2)

All patients that are considered eligible for TheraSphere treatment, and for whom an order form is completed, will be registered in the Electronic Data Capture (EDC) system.

- If TheraSphere **is reimbursed** and administered, and the patient **does not oppose** to data collection. All patient data is collected and eCRF forms are completed
- If **TheraSphere is reimbursed and administered**, and the **patient opposes** to data collection. Only the “Eligibility“ form must be completed in the eCRF and no more data is collected
- If **TheraSphere is reimbursed (ordered and delivered) but not administered**, Only the “Eligibility“ and “end of study” forms are completed and no more data is collected

8.6.3 Eligibility: Patient Information process for non opposition

Patients who have been scheduled to receive **TheraSphere treatment with a reimbursed dose**, must receive a Patient Information Sheet (PIS), and the non-opposition for data collection must be documented by the physician in the patients record.

Data collection for the registry can only begin once the PIS has been reviewed by the patient and the non-oppposition for data collection recorded.

The investigator or delegate will review with the patient the data types that will collected for the registry and the patient will have the opportunity to ask any questions about how the data will be used.

In the case of any updates to the PIS, the patient will be informed of all revisions, particularly those relating to changes in health data collection, and will be required to read the amended version of the PIS and their non-opposition for the continued collection of their health data recorded.

8.6.4 Baseline / Visit 1

The following data will be collected:

- Patient Characteristics
  - Gender
  - Body Mass Index (BMI)
  - Weight
  - Height
  - Main comorbidities (cardiac, pulmonary, renal, overweight/obesity, metabolic, other relevant disease)
- Treatment goal/expectation description: to improve (non-exhaustive): QoL, survival, symptoms of disease, disease control, tumour response, improve extension of PVT
- QoL assessment: FACT-Hep questionnaires.

  FACT-Hep will enable functional assessment of cancer therapy and questionnaire completion will take place at baseline.
- Liver disease description: liver status (normal liver, fibrosis, cirrhosis), etiology of underlying liver disease (alcoholism, viral infections…), anti-viral treatment administered, previous liver disease decompensation (ascites, bleeding…).
- Liver cancer history: tumour histology, date and method of diagnosis (biopsy, imaging). Prior anti-cancer therapy (surgery, local treatment, intraarterial treatment, radiation therapy, systemic treatment).

- Tumour Characteristics (use the most recent pre-treatment imaging and standard of care images collected closest to the treatment administration):
  - Method of tumour evaluation: CT or MRI
  - Description of lesion: Solitary /multifocal disease/diffuse/infiltrative
  - Number of lesions
  - Size largest lesion (longest diameter)
  - Location of lesion.
  - Estimation of liver tumour burden
  - Presence or absence of PVT grading (Vp0 to Vp4), and type of PVT (tumour or bland) (Appendix 1)
  - Location of EHD (if present)
    Determine whole liver volume and remnant liver volume using SPECT-CT or baseline CT or MRI and an appropriate volumetric software
- Biochemistry and coagulation test: Baseline serum bilirubin (total), albumin, coagulation (prothrombin time (PT) or prothrombin ratio or INR) ALT/AST, creatinine
- Tumour marker: AFP, CEA, CA19-9
- ECOG Performance Status (Appendix 2)
- Liver function and tumour scores: ascites, encephalopathy, Child-pugh score, BCLC stage. (Appendix 3)
- Collection of concomitant systemic treatment information, if applicable

8.6.5 Treatment Visit Data Collection / Visit 2

Pre-treatment information

- Treatment number
- ECOG (only if previous collection performed more than one month prior to treatment visit).
- Biochemistry and coagulation test: Total Bilirubin, coagulation, albumin, AST/ALT, AFP, creatinine (only if previous collection performed more than one month prior treatment visit).
- Ascites, encephalopathy, Child Pugh score and BCLC score (only if previous collection performed more than one month prior to treatment visit).
- Liver volumes: whole liver and remnant liver volume (non perfused liver)
- Multi compartment dosimetry performed or not
- Collection of concomitant systemic treatment information, if applicable

Pre treatment angiography and ^{99m}Tc-MAA imaging

- Date
- Vascular access: femoral/radial/humeral
- Coils placement
- Infusion and imaging of ^{99m}Tc-MAA:
  - Location of ^{99m}Tc-MAA injection (vessel identity)
  - Time of SPECT/SPECT CT imaging
  - Extrahepatic deposition description
  - Association of lesion location on baseline CT/MRI and location of lesions targeted by pre-treatment ^{99m}Tc-MAA (^{99m}Tc-MAA SPECT or ^{99m}Tc-MAA SPECT/CT imaging)

   - Success of PVT targeted with ^{99m}Tc-MAA (i.e., greater activity than surrounding treated liver parenchyma) on ^{99m}Tc-MAA SPECT or ^{99m}Tc-MAA SPECT/CT imaging.

TheraSphere order form

- Number of vials ordered
- Radiation activity of each vial ordered

TheraSphere Administration

- Vascular acess: femoral/radial-humeral
- TheraSphere specific treatment parameters
   - Calibration date
   - TheraSphere treatment date
   - Treatment time
   - Total activity administered
   - Type of infusion
   - Catheter position
   - Date of SPECT or SPECT/CT or PET/CT or PET/MRI imaging
   - Association of lesion location on baseline CT/MRI and location of lesions targeted by Y-90 (SPECT/CT or PET/CTor PET/MRI imaging);
   - Agreement of lesion location on pre-treatment ^{99m}Tc-MAA SPECT or SPECT/CT and targeted Y-90 SPECT/CT or PET/CT or PET/MRI,
   - Success of PVT targeting by Y-90 (i.e., greater activity than surrounding treated liver parenchyma) on Y-90 SPECT/CT or PET/CT or PET/MRI imaging
   - Lung shunt fraction

Dosimetry: Pre Treatment

- Standard dosimetry

   - Define the treated volume utilising one of the method described in section 8.3.1
   - Define whole liver and future remnant liver volume (non perfused liver)
   - Calculate the activity to be administered
   - Determine the estimated lung shunt fraction and lung absorbed dose
   - Using ^{99m}Tc-MAA (SPECT or SPECT/CT) describe the distribution of ^{99m}Tc-MAA in the tumour according to the score described in appendix 5

- Multi compartment dosimetry determination: to be performed with Y-90 (PET/CT or PET/MRI)

   - Using ^{99m}Tc-MAA (SPECT or SPECT/CT) describe the distribution of ^{99m}Tc-MAA in the tumour according to the score described in appendix 5
   - Define Volume(s) of interest (VOI*) utilising one of the method described in section 8.3.1
   - Define whole liver and remnant liver volume (non perfused liver)
   - Determine the estimated lung shunt fraction and lung absorbed dose
   - Perform dosimetric analysis in the volume(s) of interest (VOI):
      - Calculate the planned absorbed doses:
         - In the perfused liver volume
         - In the tumour(s)
         - In non tumoural perfused liver and the whole liver normal tissue

Dosimetry: Post-Treatment

- Multi compartment dosimetry
    - Using ^{99m}Tc-MAA (SPECT or SPECT/CT) describe the distribution of ^{99m}Tc-MAA in the tumour according to the score described in appendix 5
    - Define Volume(s) of interest (VOI*) utilising one of the method described in section 8.3.1
    - Perform dosimetric analysis in the volume(s) of interest (VOI):
        - Calculate the absorbed dose:
            - In the perfused liver volume(s)
            - In the tumour(s)
            - In non tumoural perfused liver and the whole liver normal tissue

Adverse Events

Adverse events will be recorded in the CRF:
- All SAEs, related or not, will be collected from any TheraSphere administration during ‘initial 12 month period’, then only related SAEs will be collected during ‘Follow-up Period’ (Section 9.0).
- AEs grade 3 or higher, related to device or device administration that occur up to 90 days after every TheraSphere administration or the first follow-up visit (if after 90 days).

8.6.6 Initial 12 Month Period: Post-Treatment Follow-up Visits (collected until 12 months)

The following data points will be captured in the eCRF where available:

- ECOG performance status
- Presence or absence of ascites, encephalopathy, Child Pugh Score, BCLC Stage
- Treatment goal assessment
- Qualitative response assessment
- Biochemistry and coagulation: Total serum bilirubin, albumin, coagulation (PT or Prothrombin Ratio ro INR) ALT/AST, tumour markers, creatinine.
- All SAEs, related or not, will be collected from any TheraSphere administration until month 12, then only related SAEs will be collected (Section 9.0).
- AEs grade 3 or higher, related to device or device administration that occur up to 90 days after every TheraSphere administration or the first follow-up visit (if after 90 days).QoL assessment using FACT-Hep questionnaires
- Subsequent TheraSphere treatment or other anti cancer treatment
- Collection of concomitant systemic treatment information, if applicable

8.6.7 Final Visit (or to be completed at study withdrawal)

A final data collection will be perfomed at the date of patient completion of the initial 12 month period or a the data of study withdrawal (will not attend anymore follow-up visits related to TheraSphere treatment).

Data collection will not be required if any of the following apply:

- Patient opposes further data collection
- Patient is lost to Follow-up:
  - Two documented attempts should be made to contact the patient before the patient is determined as lost to follow up
- The final Follow-up visit is no longer possible:
  - Patient being cared for in another institution
  - Patient is being treated with best supportive palliative care

If the final visit occurs before the 12 month treatment anniversary, the following data will be captured in the eCRF where available; data from the closest hospital visit should be entered:

- ECOG performance status
- Presence or absence of ascites, encephalopathy, Child Pugh Score, BCLC Stage.
- Biochemistry and coagulation: Total serum bilirubin, albumin, coagulation (PT or Prothrombin Ratio or INR) ALT/AST, tumour markers, creatinine
- Treatment goal assessment
- Qualitative respons assessment
- All SAEs, related or not, will be collected from TheraSphere administration during the initial 12 month period (Section 9.0)
- AEs grade 3 or higher, related to device or device administration that occur up 90 days after every TheraSphere administration or the first follow-up visit (if after 90 days).
- QoL assessment using the FACT-Hep questionnaire
- Survival status Survival Status: Deceased or alive (Section 8.6.9)
- Collection of concomitant systemic treatment information, if applicable
- Subsequent anti-cancer therapies (treatment type and start date)

8.6.8 Follow-up Period: Post treatment follow-up after 12 months

The following data points will be captured in the eCRF where available:

- All related SAEs will be collected (Section 9.0).
- Subsequent TheraSphere treatment or other anti cancer treatment, if applicable
- Collection of concomitant systemic treatment information, if applicable
- QoL assessment using the FACT-Hep questionnaire, collected at every follow-up visit until the patient receives subsequent anticancer treatment, receives best supportive palliative care, until it becomes impossible to collected the questionnaire (patient followed in another institution, lost to follow-up)
- ECOG, if possible
- Reason for study withdrawal, if applicable

8.6.9 Survival Status: Deceased or alive

Survival status will be collected for every patient included in the registry, except for those patients, who after inclusion in the registry oppose further data collection.

Additionally, survival status will be collected at the end of the study, and will recorded upon death or opposition to data collection, whichever occurs first.

For the patient alive at survival status time points, disease status will be collected (response, stable disease, recurrence, further treatment administered, best supportive palliative care).

9.0 ADVERSE EVENTS

9.1 CONTACT FOR VIGILANCE:

Contact Safety at PROACTIFSAFETY@bsci.com

9.2 ADVERSE EVENT (AE) DEFINITIONS

Adverse experience will be considered synonymous with the term adverse event and vice versa.

An AE is any untoward medical occurrence or undesirable event(s) experienced in a patient or clinical investigation patient that begins or worsens following TheraSphere administration whether or not considered related to the treatment by the investigator.

An undesirable event(s) can be, but is not limited to, symptoms experienced by a patient or objective findings, such as significant clinical laboratory abnormalities.

9.3 DEFINITIONS OF SAE/SADE/ADE/USADE/DEVICE DEFFICIENCY FOR DEVICES

9.3.1 Serious Adverse Event (SAE)

A SAE is an adverse event that:

- led to a death
- led to a serious deterioration in health that either:
  - resulted in a life-threatening illness or injury, or
  - resulted in a permanent impairment of a body structure or a body function, or
  - required in-patient hospitalisation or prolongation of existing hospitalisation, or
  - resulted in medical or surgical intervention to prevent life threatening illness or injury or permanent impairment to a body structure or a body function
- led to fetal distress, fetal death or a congenital abnormality or birth defect

NOTE 1: This includes device deficiencies that might have led to an SAE if a) suitable action had not been taken or b) intervention had not been made or c) if circumstances had been less fortunate. These are handled under the SAE reporting system.

NOTE 2: A planned hospitalisation for pre-existing condition, or a procedure required as by usual practice, without a serious deterioration in health, is not considered to be a SAE.

NOTE 3: Death due to disease progression is not considered a SAE

9.3.2 Serious Adverse Device Effect (SADE)

A serious adverse device effect is defined as an AE that is related or potentially related to the dose of TheraSphere administered and has resulted in any of the consequences characteristic of a SAE, as defined in Table 3.

Table 3: Anticipated Serious adverse device effects defined as related to the dose of TheraSphere administered

**Serious Adverse Device Effect***
Abdominal pain
Ascites
Pleural effusion
Nausea
Edema
Fatigue
Malaise
Elevated bilirubin
Elevated SGOT/CGPT
Elevated alkaline phosphatase
Hepatic encephalopathy
Elevated LDH
Elevated prothrombin time
Decreased platelets
Gastrointestinal bleed
Hemorrhage, not otherwise specified
Radiation hepatitis
Hepatic decompensation
Liver failure
Hepatorenal failure
Death, not otherwise specified

* These SADEs are serious only if they warrant medical or surgical intervention to preclude permanent injury or loss of a body function.

9.3.3 Adverse Device Effect (ADE)

An adverse device effect is an AE related to a medical device including device procedure and includes but not limited to any event resulting from device deficiencies such as insufficiencies or inadequacies in the instructions for use or the deployment, implantation, installation or malfunction of the device; any event that is the result of user error and is not considered a SADE as defined above.

9.3.4 Unanticipated Adverse Device Effect (UADE)/Unanticipated Serious Adverse Device Effect (USADE)

For the purpose of the registry, an UADE is an AE which is related to the use of TheraSphere and which by its nature, incidence, severity and outcome has not been identified in the IFU or Investigator Brochure or the protocol.

An unanticipated SADE is an UADE that meets one or more of the serious criteria outlined in Section 9.3.1. All UADEs must be reported in an expedited fashion as described in Section 9.3.

9.3.5 Device Deficiency

A device deficiency is an inadequacy of a medical device with respect to its identity, quality, durability, reliability, safety or performance. Device deficiencies include malfunctions, user errors and inadequate labeling.

A device malfunction is the failure of a medical device to perform in accordance with its intended purpose when used in accordance with the IFU.

9.4 Recording of AEs and SAEs

Grade 3 or higher adverse events (AEs) (graded using NCI-CTCAE v 5.0) related or possibly related to the device, or the device administration procedure, that occur up to 90 days after every TheraSphere administration or the first follow-up visit post TheraSphere administration will be captured in the eCRF.

All SAEs, related or not, will be documented in the eCRF starting from the date of TheraSphere administration during the ‘Initial 12 Month Period’ and only related SAEs will be documented in the eCRF during the ‘Follow-up Period. If unable to access the eCRF the SAE can be reported via email (PROACTIFSAFETY@bsci.com) and the eCRF updated within 2 days of the email report.

A symptom or condition preceeding administration of TheraSphere will not be captured as an SAE unless the event increases in severity after administration of TheraSphere and the increase in severity is considered related or possibly related to TheraSphere or its administration.

Any medical management of an event and the date of resolution of the event must be recorded in the eCRF using medical terminology according to NCI-CTCAE v. 5.0 terminology.

For each AE, the following information will be recorded:

- AE term according to MedDRA (Medical Dictionary for Regulatory Activities)
- Seriousness criteria
- Severity
- Action taken
- Relationship to study procedure (possible or probable or definitely related)
- Relationship to study device (possible or probable or definitely-related)
- Date of onset
- Date of resolution
- Outcome of event

9.5 Causality (Relationship to Device or Procedure) Assessment

The investigator or physician sub-investigator must indicate whether he/she believes the AE/SAE is possibly related (reasonable possibility), probably related, or definitely related to the device or procedure (i.e. the device or device administration procedure caused the AE/SAE).

All SAEs that are possibly, probably or definitely related to the device as determined by the investigator or physician sub-investigator, must be reported to the Sponsor and evaluated for post-marketing regulatory reporting in accordance with BTG-SP-05.010 Medical Device Vigilance and

Reporting. All SAEs will be evaluated for post-marketing reporting requirements in accordance with the applicable regulatory requirement (i.e. MEDDEV (Medical Devices Directive)).

9.6 Suspected Device Malfunction or Deficiency

In case of a suspected device malfunction or deficiency in performance that is not associated with an SAE or AE, the investigator or physican sub-investigator must report the event to the Sponsor and Vigilance via the eCRF within 48 hours of learning of the event (if unable to access the eCRF, the suspected device malfunction or deficiency can be reported via email (PROACTIFSAFETY@bsci.com) and the eCRF updated within 2 days of the email report) (Section 9.1). The event will be investigated as per the applicable Customer Complaints procedure.

9.7 Submitting Expedited Safety Reports

Any SAE, SADE or UADE (defined previously) considered to be device or procedure related must be reported via the eCRF to the sponsor within 24 hours of learning of the event (if unable to access the eCRF, the SAE can be reported via email (PROACTIFSAFETY@bsci.com)(and the eCRF updated within 2 days of the email report). In addition, reporting to the Ethics Committee (EC) may be required as defined by the local institutional practices.

The SAE form should be completed in the eCRF and will constitute the source for the SAE report.

The SAE form must include the same information as described in section 9.4. In the free text box the investigator must document other information, such as: event description, associated symptoms, medication, procedure, biological data, imaging, any action taken. This information should also be documented in the patient file including the hospitalisation report or the consultation report.

The entire SAE form needs to be completed in English and in accordance with Good Documentation Practices to keep requests for additional information to a minimum.

Patients experiencing SAE, SADE or UADE should be followed as medically appropriate and in line with usual medical practice, until medical treatment and/or medical monitoring of the event is no longer required because the event resolves or stabilises, returns to baseline (if a baseline value is available), can be reasonably attributed to agents other than the study procedure or device or a referral for appropriate follow-up care has been made.

The investigator must promptly inform the EC of all UADEs and SAEs as required by their local EC. These events will be reported by the sponsor as appropriate to the regulatory authorities according to relevant jurisdictional medical device regulations.

All SAEs related to the device or procedure will be entered into the Quality System and evaluated for post-market medical device reporting in accordance with MEDDEV 2.12-1 rev 8 and 21 CFR 803.

9.8 Anticipated Adverse Events

Adverse events that might reasonably be anticipated to be TheraSphere-related or procedure-related as reported from sponsored clinical trials and the published literature are summarised below in Table 4.

Table 4: Adverse Events reported as TheraSphere device or procedure-related

Frequency	Description of Adverse Event (per NCI-CTCAE v 3.0)
Common ≥10%	Fatigue, pain, nausea, vomiting, anorexia and laboratory value abnormalities including increased phosphate, AST, ALT, bilirubin, hypoalbuminemia and lymphopenia with no clinical sequellae
Infrequent ≤10%	Constipation, heartburn, weight loss, fever, ascites, muscle weakness, variations in creatinine, platelets, hemoglobin and leukocytes, GI ulcer, dyspnea, supraventricular arrhythmia, diarrhea, hypotension, insomnia, rigors/chills, sweating, distension, GI obstruction, hematoma, GI hemorrhage, pleural effusion, hyponatremia, dehydration, allergic reaction, GI other, neurology other
Rare ≤1%	Alopecia, bruising, pruritis, rash, hot flashes, taste alteration, hemorrhage, liver dysfunction, infections, dizziness, mood alteration, sensory neuropathy, somnolence, urine color change, intraoperative injury, flu-like symptoms, tumour lysis syndrome, thrombosis, metabolic/laboratory abnormalities: neutrophils, hypercalcemia, hyperglycemia, hyperkalemia, hypermagnesemia, lipase, lymphatics other, pulmonary other vascular other, death

In addition, the following treatment-emergent events whose relationship to the use of TheraSphere or the administration procedure cannot be proven have been reported in clinical trials of treatment of primary or secondary liver cancer:

Abdominal pain, abdominal distention, anxiety, blurred vision, bladder infection, lower extremity edema, gastrointestinal stoma complication including mild pain, hepatic encephalopathy, hepatorenal failure, mesenteric vein thrombosis, edema, malaise, hepatic decompensation, hepatitis, cholangitis, bacterial cholangitis, duodenal ulcer, hypertension, portal hypertension, aspiration pneumonia, fall, gastrointestinal bleeding, perforated gastric ulcer, deterioration of general health status, elevated CEA, elevated LDH, elevated prothrombin time, elevated BUN, bacterial sepsis, hypoglycemia, abnormal platelets and electrolyte disturbances including hypercalcemia, hyperkalemia, hypo-magnesemia, hyponatremia, low serum bicarbonate and low serum chloride.

10.0 STATISTICAL CONSIDERATIONS

10.1 STUDY DESIGN AND DETERMINATION OF SAMPLE SIZE

A formal sample size computation was not performed for this real world registry study. All patients that have been treated and reimbursed will be included.

10.2 STATISTICAL ANALYSIS

The statistical analysis plan (SAP) will be a separate document and will be updated as required, in association with any protocol amendments. The SAP will include descriptions of tables, listings and figures and will describe statistical programming considerations.

10.2.1 Analysis Populations and Sub-Groups

The treated population will comprise all patients who have received a reimbursed dose of TheraSphere and are not opposed to their data being collected.

The dosimetry population will comprise of patients in the treated population with dosimetry data available. Dosimetry data will be collected for all patients.

All analyses will be performed on the treated population, except for dosimetry analyses. All analyses will also be performed according to the disease indication (HCC, mCRC , iCC).

Analyses of study outcomes will also be performed on the following subgroups of interest:

For All patients:

- Age group (≥18 to <65 years, ≥65 to <75 years, ≥75 years)
- Unilobar or bilobar disease at baseline
- ECOG status (0, >0) at baseline
- Albumin/bilirubin (ALBI) score (1, 2 or 3) at baseline
- Liver tumour burden at baseline (<25%, ≥25%)
- Target lesion size (≤ 5 cm vs >5 cm, ≤7 cm vs >7 cm, ≤ 10 cm vs >10 cm)
- Selective versus non-selective (lobar or whole liver) administration
- Standard versus multi compartment dosimetry treatment

For HCC patients:

- Etiology of underlying liver disease
- Child Pugh score (A or B) at baseline for cirrhotic patients
- Cirrhosis versus no cirrhosis
- Prior TACE treatment (Yes, No)
- PVT classification (Vp0, Vp1 or Vp2, Vp3 or Vp4) at baseline
- BCLC stage (B, C) at baseline
- Prior systemic treatment, including sorafenib (Yes, No)
- AFP (<200 ng/ml, ≥200 ng/mL, <400 ng)/mL, ≥400 ng/mL) at baseline

- Threshold absorbed doses to the tumour ≥ 205, < 205 Gy and ≥250, <250 Gy (by local and central assessment)

For mCRC patients:

- CEA (<2xULN, ≥ 2xULN) at baseline
- Previous line of systemic chemotherapy (≤ 2, >2)
- Prior local or/and locoregional treatment (Yes, No)
- Concomitant chemotherapy versus non concomitant chemotherapy
- Threshold absorbed doses to the tumour <100 and ≥ 100 Gy (by local assessment)

For iCC patients:

- Prior resection (Yes, No)
- CA 19-9 (<2xULN, ≥ 2xULN) at baseline
- Cirrhosis versus no cirrhosis
- Concomitant chemotherapy versus non concomitant chemotherapy
- Threshold absorbed doses to the tumour <205, 205-250 Gy, >250 Gy (by local and central assessment)

10.3 Baseline and Demographic Characteristics

Demographic data and baseline characteristics will be summarised. Continuous data will be summarised with means, medians, standard deviations, minima and maxima. Categorical data will be summarised with observed counts and percentages for each category.

Length of follow-up will be summarised as mean, median, standard deviation, minimum and maximum. The median length of follow-up will also be computed using the reverse Kaplan-Meier method.

10.4 Effectiveness Analyses

All effectiveness outcomes will be assessed in the treated population.

A Kaplan Meier (KM) analysis will be performed for OS and median OS will be computed with a corresponding 95% confidence interval (CI).

Univariable and multivariable Cox regression analyses of OS will be performed to assess the impact of the subgroup factors listed above.

The number of patients achieving their treatment expectation will be summarised as observed counts and percentages, with corresponding 95% CI.

Tumour marker response will be summarised as observed counts and percentages, with corresponding 95% CI.

Qualitative tumour response will be summarised as the number and percentage of patients having a response (CR or PR), with corresponding 95% CI.

Univariable and multivariable logistic regression analyses of binary effectiveness endpoints (i.e. achievement of treatment expectation, tumour marker response and qualitative tumour response) will be performed to assess the impact of the subgroup factors listed above.

10.5 SAFETY ANALYSES

Incidence of SAEs and grade 3 or higher AEs, coded according to MedDRA (Medical Dictionary for Regulatory Activities), will be tabulated. Descriptive summaries of laboratory results, including changes from baseline, will be presented by study visit. The number of re-hospitalisations related to TheraSphere treatment will be summarised as observed counts and percentages and the duration of re-hospitalisations will be summarised with means, medians, standard deviations, minima and maxima.

10.6 DOSIMETRY ANALYSES

The following outcomes will be summarised as observed counts and percentages for local assessments in all indications and for central assessments in HCC and iCC patients only:

- Association between tumour location at baseline and location of lesions targeted by ^{99m}Tc-MAA (SPECT or SPECT/CT)*
- Association between tumour location at baseline and location of lesions targeted by Y-90 PET/CT, Y-90 PET/MRI or SPECT/CT*
- Association of lesion location based on ^{99m}Tc-MAA (SPECT or SPECT/CT), and location of lesions targeted by Y-90 using post-treatment PET/CT, PET/MRI or SPECT/CT*
- Association between PVT at baseline and PVT targeting by ^{99m}Tc-MAA (SPECT or SPECT/CT), Y-90 (PET/CT or PET/MRI or SPECT/CT), when applicable
- DVH for total perfused tumour, index lesion and whole normal liver tissue, using ^{99m}Tc-MAA (SPECT or SPECT/CT) and Y-90 (PET/CT or PET/MRI), when applicable

* A score that describe the intensity of distribution of ^{99m}Tc-MAA / Y-90 in the tumour versus normal tissue, and the tumour coverage with ^{99m}Tc-MAA / Y-90 in tumour will be created (Appendix 5).

In patients with HCC and iCC, a Cox regression analyses of OS will be performed to assess the impact of the tumour absorbed doses. This will be done separately for absorbed doses determined by ^{99m}Tc-MAA (SPECT or SPECT/CT) and by post-treatment Y-90 (PET/CT or PET/MRI). Similarly, in patients with mCRC, a similar cox regression analysis of OS will be performed to assess the impact of absorbed dose to tumours and liver volume.

In patients with HCC and iCC, a logistic regression analysis of qualitative tumour/index lesion response (CR or PR) will be performed to assess the impact of the tumour/index lesion absorbed doses. This will be done separately for tumour/index lesion absorbed doses determined by ^{99m}Tc-MAA (SPECT or SPECT/ CT) and determined by post-treatment Y-90 (PET/CT or PET/MRI). Similarly, in mCRC patients, a logistic regression analysis of qualitative tumour/index lesion response will be performed to assess the impact of the absorbed dose to perfused tumours and liver volume.

In patients with HCC and iCC, a logistic regression analyses of the occurrence of SAEs will be performed to assess the impact of the normal tissue liver absorbed doses. This will be done separately for absorbed doses from pre-procedural ^{99m}Tc-MAA (SPECT or SPECT/CT) and for post-treatment Y-90 (PET/CT or PET/MRI). Similarly, in patients with mCRC, logistic regression analyses of the occurrence of SAEs will be performed to assess the impact of absorbed dose to normal tissue liver.

In patients with HCC and iCC, the relationship between absorbed doses derived from post-treatment Y-90 (PET/CT or PET-MRI) and from pre-procedural ^{99m}Tc-MAA (SPECT or SPECT/CT) will be assessed separately for normal tissue liver absorbed doses (perfused liver and total liver) and tumour absorbed doses using Bland-Altman analysis. Similarly, in patients with mCRC, the relationship between absorbed dose to perfused liver volume will be assessed for absorbed dose to perfused liver volume and absorbed dose to normal tissue liver.

In patients with HCC and iCC, a linear regression of absorbed dose from pre-procedural ^{99m}Tc-MAA (SPECT or SPECT/CT) imaging and post-treatment Y-90 (PET/CT or PET/MRI) imaging will be performed and Pearson's correlation coefficient will be calculated. Similarly, in patients with mCRC, a linear regression will also be performed for absorbed dose to perfused liver volume and absorbed dose to normal tissue liver.

10.7 Quality of Life

QoL scores of each domain and each question at each time-point and their differences from baseline will be summarised. A deterioration in QoL is defined as a 7-point decline in the total score or death, whichever comes first. The time to deterioration in QoL will be calculated as the interval between first date of TheraSphere treatment and deterioration in QoL. If a patient is lost to follow-up, the patient will be considered as a death in the time to deteriation analysis. A KM analysis will be performed and median value will be computed with corresponding 95% CI.

10.8 Other Analyses

The following outcomes will be summarised as observed counts and percentages:

- The number of patients receiving a post TheraSphere anti-cancer treatment.
- The number of patients receiving a post TheraSphere best supportive care treatment.
- The type of vascular access (radial/femoral) used to administer TheraSphere.

10.9 Interim Analyses

Interim analyses will be performed when sufficient data have been completed in the eCRF to support interim study publications.

Table 5: Schedule of Study Analyses

Analysis number	Based on first enrolled patient until	Data to be included in analysis
1st Interim analysis	1 year	Baseline characteristics, TheraSphere treatment and safety data
2nd Interim analysis	2 years	All data

3rd Interim analysis (5 year clinical study report to HAS)	4 years	All data
Final analysis	5 years (after last patient enrolled)	All data

Note: 1st Interim analysis will only have data available to HCC as this was prior to the addition of iCC and mCRC indications.

10.10 Final Analyses

Final analysis will be performed after 5 years of enrollment of the first patient, which will include data for all patients included in the registry until the registry closure (31 Dec 2023) plus a follow-up period of one year for the last patient included.

11.0 DATA MANAGEMENT

Data from the study will be collected via an EDC system hosted. Clinical data will be held in a secured, validated system by the EDC vendor DataTrak or Medidata RAVE and can be downloaded by Data Management (DM) on an ongoing basis. All processes will be documented in the Data Management study files and forwarded to BTG for storage in their study area.

Data will be entered in the eCRF at the study site by trained study personnel.

System trained study personnel will be responsible for entering data on the observations, tests and assessments captured in the eCRF system and according to the eCRF completion guidelines. The eCRF completion guidelines will also provide the study site with data entry instructions. Data entered in the eCRF will be immediately saved to a central database and changes tracked to provide an audit trail. When data have been entered, reviewed and edited the investigator will be notified to sign the eCRF electronically as per the agreed project process and data will be locked to prevent further editing. A copy of the eCRF will be archived at the study site.

Data verification will be performed by DM and data validation checks will be created, with the sponsor team performing User Acceptance Testing on them before they go live. DM will be responsible for managing any programmed edit checks that fire as well as DM manually created ones. Regular metrics on the study will be provided to the study team, including clinical, that can be shared with the sites to assess progress and show what still needs to be completed. The reports that will be provided will be documented in the relevant Plan. A clean database will be declared by Data Management after consistency checks have been run, all coding has been completed and approved, all the data in the database has been accounted for and all edit checks have been run and data discrepancies have been resolved or accepted. Any third party data has been reconciled and deemed clean. After the database has been declared clean, it will be locked and editing in the database will only be allowed with the proper documentation.

After database lock, data will be extracted to SAS® (SAS Institute, Inc., Cary, NC, USA) for analysis as defined in the SAP. SAEs will be entered into the appropriate Pharmacovigilance database.

AEs and concomitant diseases will be coded according to the version of MedDRA agreed with Biocompatibles UK Ltd. Concomitant medications will be coded using the agreed version of the WHO Drug dictionary.

12.0 LEGAL/ETHICS AND ADMINISTRATIVE PROCEDURES

12.1 GOOD CLINICAL PRACTICE/REGULATORY COMPLIANCE

This study will be conducted in compliance with standard operating procedures of the sponsor or designee. All procedures set out in the this study protocol, pertaining to the documentation of this study, are designed to ensure that the sponsor and investigators abide by the Declaration of Helsinki, Good Clinical Practice (GCP) as described in 21 CFR Parts 11, 50, 56, each participating country's legislation, as well as in the International Council for Harmonisation (ICH) Harmonised Tripartite Guideline E6 (R2): Good Clinical Practice and International Standard ISO 14155 Clinical investigation of medical devices for human patients: Good Clinical Practice.

It is the investigator's responsibility to ensure that adequate time and appropriate resources are available at the study site prior to participation in this study. The investigator will maintain a list of appropriately qualified personnel to whom the investigator has delegated study related tasks.

12.2 STUDY SITE AND INVESTIGATOR QUALIFICATION

This treatment will be performed by qualified investigators at multiple sites in France. All treated sites will be reviewed by the sponsor to verify that they are able to conduct the treatment.

12.2.1 Statement of Investigator

The investigator will be required to sign and date a Statement of Investigator form provided by the sponsor for the original and each subsequent amendment of the protocol and return the original signed document to the sponsor. A copy of the signed form will be kept with the investigator for his/her files.

12.2.2 Site qualifications

The Institution must have appropriately qualified staff in place to adequately complete the treatment. The following need to be in place for site qualification.

- The use of TheraSphere is carried out in accordance with the decree number 2007-389 of March 21st 2007 relative to the technical operating conditions applicable to cancer care activity.
- TheraSphere is used by multidisciplinary teams that include: a nuclear physician, an interventional radiologist with the expertise of hepatic embolisation in oncology, a radiophysicist and a radiopharmacist. This activity must be carried out in centres with sufficient infrastructure to be authorised by the French Nuclear Safety Authority (Agence de Sécurité Nucléaire - ASN) to carry out internal radiation activities.
- The decision to perform the treatment and the post-treatment follow-up has been taken, under patient agreement and after a positive treatment recommendation of the local/regional multidisciplinary tumour board (MTB), specialised in HCC. The MTB must include at least: an oncologist, a hepatologist, or a hepatologist skilled in oncology, a surgeon specialist in liver surgery, an interventional radiologist, a nuclear medicine specialist, a radiation oncologist, and a palliative care specialist.

- The patient must be able to benefit from the availability of a supportive care team.

Every investigator will need to comply with the Declaration of Helsinki, and the local data protection laws, which means ensuring the investigator understands the need for adequate medical records as well as the need to inform and obtain the patient's agreement, and to ensure data is collected and processed in a confidential manner.

In addition, all participating study sites must be appropriately experienced in the use of Y-90 microspheres for the treatment of liver tumour and must have completed adequate training specified by Biocompatibles UK Ltd. The same applies for the use of Simplicit^{90}Y™ software, where applicable.

12.3 Independent Ethics Committee (IEC)

It is the responsibility of the sponsor to submit this protocol, the informed consent document (approved by the Sponsor or designee), relevant supporting information and all types of patient recruitment information to the IEC for review and approval prior to site initiation.

A copy of the written approval of the protocol and Patient Information Sheet (PIS) document must be received by the sponsor prior to initiating consent discussion with the first patient. Prior to implementing changes to the study, the sponsor and IEC must also approve any revised Patient information Sheets and amendments to the protocol with documentation of the approvals submitted to the sponsor. The approval document should clearly state the study reference, date of review and actions taken.

The sponsor, will be responsible for keeping the IEC apprised of any changes to the protocol, any deviations from the protocol and SAEs as required by the IEC for this type of registry study.

12.4 Information document and data collection non-opposition

It is the responsibility of the investigator to discuss the requirements of the Registry Study with the patient and to record their non-opposition to their data collection in the patient medical records prior to any data entry. A copy of the PIS document must be provided to the patient and filed at site for verification by sponsor assigned personnel.

A traceability of information documentation provided and the patient non opposition must be documented in the patient medical record and must be available for verification by the study monitors, the regulatory authority and any authorised personnel at any time.

12.5 Patient Privacy and Confidentiality

The sponsor and investigator affirm and uphold the principle for the patient's right to protection against invasion of privacy. Throughout this study, all data collected and analysed by the sponsor or designee will be treated confidentially and identified by an identification number.

To verify compliance with the protocol, the sponsor will require that the investigator permits its designee access to the patient's primary medical record to review those portions that directly concern

this study (including but not limited to laboratory test results, radiology images, and hospital and outpatient records).

The patient must be informed that his/her records will be reviewed by the sponsor, sponsor representative and/or a representative of the appropriate regulatory agency and Scientific Advisory Board. The treatment information document will also state that patient privacy will be maintained pursuant to the Health.

Data collected during this study may be used to support the development, registration or marketing of TheraSphere. Collected data may be reviewed by the sponsor and/or its representatives, independent auditors who validate the data on behalf of the sponsor, third parties with whom the sponsor may develop, register or market TheraSphere, national or local regulatory authorities and the IEC who granted approval for this registry to proceed.

12.6 Study Monitoring

Monitoring will be detailed in the Monitoring Plan and for the registry, will be performed by qualified sponsor assigned personnel. Data will be monitored on site and remotely. At the monitoring visits, the progress of the study will be discussed with the Investigator or his/her representative. The patient information and non-opposition to data collection documentation will be reviewed and the CRF pages will be checked for completeness and accuracy. Patient source data must be available for review. The Investigator and his/her staff are expected to cooperate with the Study Monitor and be available during at least a portion of the monitoring visit to review the CRF pages and any queries/resolutions, answer questions and provide any missing information.

Data checks will be conducted through built-in electronic logic checks as well as manual targeted reviews performed jointly by the Data Manager; Manager, Clinical Development; Study Statistician and Project Physician. This will include checks to address missing data and inconsistencies on collected data.

Risk based monitoring modules of EDC provider may be utilised for assessing the risk for data integrity of specific data points.

Telephone and electronic mail contact will be made with the investigator and study staff as necessary during the data collection and report writing periods. The investigator and their staff are expected to cooperate with respect to data entry and responding to queries in a timely manner.

Detailed reports on data transcription activities will be run by DM and used for assessing the compliance with data entry expectations.

12.7 Modification of the Registry

All amendments to the Registry must be documented in writing, reviewed and approved by the investigator, the sponsor and submitted to the IEC for approval prior to implementation. If the protocol amendment substantially alters the data that will be collected, a new consent form must be provided to each ongoing patient for continued participation in the registry.

12.8 PROTOCOL DEVIATIONS

All deviations from the investigational plan, with the reason for the deviation and the date of occurrence, must be documented and reported to the sponsor using eCRF. Sites may also be required to report deviations to the IEC, per local guidelines and government regulations.

Deviations will be reviewed and evaluated on an ongoing basis and, as necessary, appropriate corrective and preventive actions (including IEC) notification, site re-training, or site discontinuation/termination) will be put into place by the sponsor.

12.9 RECORDING ACCESS TO AND RETENTION OF SOURCE DATA

Investigators are required to prepare and maintain adequate documentation which includes:

- Documents relative to the subject medical history that verify indications for TheraSphere treatment.
- Records covering subject participation in the trial including basic identification information, results of physical examinations and diagnostic tests, original laboratory results (initialed and dated by a physician), therapy, treatment administration, concurrent medication information, pathology reports, and patient visit notes.
- Documented evidence of a patient's non opposition to data collection

The investigator must permit authorised representatives of the sponsor, the regulatory authorities, the IEC and auditors to inspect facilities and records relevant to the study.

The sponsor or authorised representative of the sponsor, auditors, IEC or regulatory inspectors may check the eCRF entries against the source documents. The non-opposition documentation will include a free statement by which the patients allow the above-named access to source data to substantiate information recorded in the eCRFs. These personnel, bound by professional secrecy, will not disclose any personal information or personal medical information.

As described in the ICH GCP Guidelines, 'essential documents', including eCRFs, source documents, information document, laboratory test results should be retained by the investigator until at least two years following the date on which the investigation is terminated or completed. These documents should be retained for a longer period however, if required by the applicable regulatory requirements or by an agreement with the sponsor. The investigator must obtain written permission from the sponsor prior to destruction of any study document. These records must be made available at reasonable times for inspection and duplication, if required, by a properly authorised representative of the local regulatory authority in accordance with applicable local regulations or other regulatory authorities in accordance with regulatory requirements.

12.10 ELECTRONIC CASE REPORT FORMS

The investigator is responsible for maintaining the eCRF and for the accurate transcription of data into the eCRF from source documents. The eCRF has been designed to capture all observations and other data pertinent to this registry. The eCRF pages should be completed by the investigator or a delegate as stated on the Delegation of Authority Log. Overwriting of information or use of liquid correction fluid is not permitted in any source documentation associated with this registry.

Once monitored, queries may be raised if the data are unclear or contradictory. The eCRFs must be reviewed and electronically signed and dated by the investigator once all data has been entered and all queries resolved.

12.11 Publications

All manuscripts, abstracts or other modes of presentation arising from the results of the study will be prepared under guidance and approval from the Publication Review Committee, but must be reviewed and approved in writing by the Sponsor, in advance of submission per the publication plan. The review is intended to protect Sponsor proprietary information existing either at the date of commencement of the study or generated during the study. No individual Investigator may publish results from his/her site until after publication of the primary manuscript describing the full study population.

The detailed obligations regarding the publication of any data, material results or other information that is generated or created in relation to the study shall be set out in the agreement between the Investigator and Sponsor.

Publication plans for this registry lie within the remit of the Publication Review Committee and are detailed in a separate Publication Plan. Should an investigator wish to publish on any of the data sets collected in this registry, a request in writing providing a detailed outline of the intended abstract or publication must be submitted to the Scientific Steering Committee in advance for consideration. The Scientific Steering Committee will render a decision and communicate the outcome to the investigator.

In accordance with recommendations from the International Committee of Medical Journal Editors, the study will be listed in a publicly accessible registry of clinical trials such as www.clinicaltrials.gov.

12.12 Audit/Inspections

To ensure compliance with relevant regulations, data captured in this registry must be available for inspection upon request by the sponsor and its representatives and the IEC for each study site.

13.0 APPENDICES

13.1 APPENDIX 1: TABLE OF CLASSIFICATION OF PORTAL VEIN THROMBOSIS (PVT)

**Types of Portal Vein Thrombosis**	**Situation / Extent of the Thrombosis**
Vp0: Absent	No presence of tumour thrombus in portal vein
Vp1: Presence of tumour thrombus distal to, but not in, the second-order branches of the portal vein	Segmental branchs
Vp2: Presence of tumour thrombus in second-order branches of the portal vein	Sectorial branchs
Vp3: Presence of tumour thrombus in first-order branches of the portal vein	Right/left portal veins, providing the vascular flux to right or left liver is not preserved
Vp4: Presence of tumour thrombus in the main trunk of portal vein or a portal vein contralateral to the primay involved lobe (or both)	Portal trunk or right **and/or** left portal veins providing the vascular flux to right or left liver is **not** preserved

Ikai et al., 2010; Chan et al., 2016

13.2 APPENDIX 2: ECOG PERFORMANCE STATUS

GRADE	ECOG PERFORMANCE STATUS
0	Fully active, able to carry on all pre-disease performance without restriction
1	Restricted in physically strenuous activity but ambulatory and able to carry out work of a light or sedentary nature, e.g., light house work, office work
2	Ambulatory and capable of all selfcare but unable to carry out any work activities; up and about more than 50% of waking hours
3	Capable of only limited selfcare; confined to bed or chair more than 50% of waking hours
4	Completely disabled; cannot carry on any selfcare; totally confined to bed or chair
5	Dead

Developed by the Eastern Cooperative Oncology Group, Robert L. Comis, MD, Group Chair (Oken et al., 1982)

13.3 APPENDIX 3: HCC STAGE AND SCORES

Child-Pugh Score

Factor	1 point	2 points	3 points	TOTAL POINTS and SCORE
Total bilirubin (µmol/L)	<34	34-50	>50	**A 5-6**
Serum albumin (g/L)	>35	28-35	<28	
INR<br>PR	<1.7<br>>50%	1.71-2.30<br>40-50 %	>2.30<br><40%	**B 7-9**
Ascites	None	Mild<br>Diuretic sensitive	Moderate to Severe<br>Diuretic refractory	
Hepatic encephalopathy	None	Grade 1-11 (or suppressed with medication	Grade III-IV (or refractory)	**C > 9**

https://www.mdcalc.com/child-pugh-score-cirrhosis-mortality

BCLC Classification

**BCLC 0, Very Early stage**	Single ≤2 cm<br>Child A<br>ECOG PS 0
**BCLC A, Early stage**	Single or up to 3 nodules ≤3 cm<br>Preserved liver function*,<br>ECOG PS 0
**BCLC B, Intermediate stage**	Intermediate stage<br>Multinodular (more than 3 nodules, or more than one of more than 3 cm)<br>Preserved liver function*,<br>ECOG PS 0
**BCLC C, Advanced stage**	Multinodular and one of the following<br>Portal invasion or/and<br>Extrahepatic spread or/and<br>Preserved liver function*, or/and<br>ECOG PS 1–2
**BCLC D, End stage**	Child C<br>ECOG PS 3-4

Forner et al., 2018

* Preserved liver function includes a group of patients with different degrees of liver function reserve that has to be carefully evaluated. For most treatment options, compensated liver disease (without ascites) is required to obtain optimal outcomes.

13.4 APPENDIX 4: ALBI SCORE

ALBI calculation	($\log_{10}$ bilirubin [μmol/L] × 0.66) + (albumin [g/L] × −0.085)
grade 1	≤−2.60
grade 2	> −2.60 and ≤−1.39
grade 3	> −1.39 =

Johnson et al., 2015

13.5 APPENDIX 5: CATEGORISATION OF AGREEMENT BETWEEN CT/MRI BASELINE IMAGING AND ^{99m}TC-MAA DEPOSITION AND THERASPHERE DEPOSITION ON SPECT

- **Description of the association of ^{99m}Tc-MAA or Y-90 distribution and baseline imaging**

	^{99m}Tc-MAA or Y-90 distribution in perfused liver			
ITEM	Significantly higher overall perfusion in tumour versus normal liver	Slightly higher perfusion in tumour versus normal liver	Same perfusion in tumour versus normal liver	Lower perfusion in tumour versus normal liver
Complete ^{99m}Tc-MAA or Y-90 distribution in 90-100% of the tumour(s)				
Intermediate ^{99m}Tc-MAA or Y-90 distribution in 50-90% of the tumour(s)				
Poor ^{99m}Tc-MAA or Y-90 distribution in < 50% of the tumour(s)				

Optimal	Sub optimal	Non Optimal

- **Description of the association between ^{99m}Tc-MAA and Y-90 distribution**

	^{99m}Tc-MAA distribution in perfused liver		
Y-90 distribution in perfused liver	Same distribution (90 -100 % of overlap)	Intermediate (50-90% overlap)	Poor (< 50% overlap)

- **Assessment of ^{99m}Tc-MAA / Y-90 uptake on Portal Vein Thrombosis:**

^{99m}Tc-MAA or Y-90 distribution in portal vein thrombosis	strong uptake	Weak uptake	No uptake

13.6 APPENDIX 6: THERASPHERE INSTRUCTIONS FOR USE

Contact your CRA for a copy of the current Instructions for Use.

13.7 APPENDIX 7: ASCITES ASSESSMENT

Grade 0= Absence clinical or imaging (with or without diuretic treatment)

Grade 1 = Clinically absent (asymptomatic) but Trace of ascites on imaging assessment (no medical intervention needed or if the patient is on diuretic treatment, no need to modify the treatment)

Grade 2 = Mild/Moderate ascites, clinically present (symptomatic) and medical intervention needed, or if the patient is on diuretic treatment need to modify the treatment.

Grade 3 = Severe ascites, clinically symptomatic, paracentesis and diuretic treatment needed.

Grade 4 = life threatening*

* SAE to be declared

14.0 REFERENCES

- Abbott E, Nadia Falzone, Boon Q. Lee, Christiana Kartsonaki, Helen Winter, Tessa A.Greenhalgh et al. The Impact of Radiobiologically-Informed Dose Prescription on the Clinical Benefit of Yttrium-90 SIRT in Colorectal Cancer Patients. J Nucl Med May 2020; doi: 10.2967/jnumed.119.233650
- Ahmed S et al. Quality of Life in Hepatocellular Carcinoma Patients Treated with Transarterial Chemoembolisation. HPB Surg. 2016; 2016: 6120143.
- Alexandra Gangi, Jehan Shah, Nathan Hatfield, Johnna Smith, Jennifer Sweeney, Junsung Choi, Ghassan El-Haddad, Benjamin Biebel, et al. Intrahepatic Cholangiocarcinoma Treated with transarterial Yttrium-90 Glass Microsphere Radioembolization: Results of a Single Institution retrospective Study. J Vasc Interv Radiol 2018; 7:1–8
- Angela Lamarca, Paul Ross, Harpreet S. Wasan, Richard A. Hubner, Mair_ead G. et al Advanced Intrahepatic Cholangiocarcinoma: Post Hoc Analysis of the ABC-01, -02, and -03 Clinical Trials. JNCI J Natl Cancer Inst (2020) 112(2): djz071
- Angela Lamarca, Daniel H. Palmer, Harpreet Singh Wasan, Paul J. Ross, Yuk Ting Ma, and on behalf of the Advanced Biliary Cancer (ABC) Working Group. ABC-06 | A randomised phase III, multi-centre, open-label study of active symptom control (ASC) alone or ASC with oxaliplatin / 5-FU chemotherapy (ASC+mFOLFOX) for patients (pts) with locally advanced / metastatic biliary tract cancers (ABC) previously-treated with cisplatin/gemcitabine (CisGem) chemotherapy. Journal of Clinical Oncology 2019 37:15_suppl, 4003-4003 Belghiti J Treatment of hepatocellular carcinoma. Bull Acad Natl Med. 2012; 196(1): 97-102; 102-103.
- Bengtsson G, et al. Natural history of patients with untreated liver metastases from colorectal cancer. Am J Surg 1981; 141: 586-589
- Biederman D et al. Yttrium-90 Glass-Based Microsphere Radioembolisation in the Treatment of Hepatocellular Carcinoma Secondary to the Hepatitis B Virus: Safety, Efficacy, and Survival. J Vasc Interv Radiol. 2015; 26(11): 1630-8.
- Bolondi L et al. Heterogeneity of patients with intermediate (BCLC B) Hepatocellular Carcinoma: proposal for a subclassification to facilitate treatment decisions. Semin Liver Dis. 2012; 32(4): 348-59.
- Brooks R EuroQoL: The current state of play. Health Policy. 1996;37(1):53–72
- Bruix J et al. Evidence-Based Diagnosis, Staging, and Treatment of Patients With Hepatocellular Carcinoma. Gastroenterology. 2016; 150(4): 835-53.
- Bruix J et al. Management of hepatocellular carcinoma: an update. Hepatology. 2011; 53(3): 1020-2.
- Bruix J et al. Regorafenib for patients with hepatocellular carcinoma who progressed on sorafenib treatment (RESORCE): a randomised, double-blind, placebo-controlled, phase 3 trial. Lancet. 2017; 389(10064): 56-66.
- Cella D, Webster K, Chang C, Sarafian B, Linn E, Bonomi A et al. (2000) Deriving a clinically meaningful symptom index from the Functional Assessment of Cancer Therapy-Hepatobiliary (FACT-Hep) scale: The FHSI-8. Quality of Life Research, 9(3), 293
- Chan A, Poon D, Chok K. Management of hepatocellular carcinoma with portal vein tumor thrombosis: Review and update at 2016. World J Gastroenterol. 2016 Aug 28; 22(32): 7289–7300.
- Chauhan N, Mulcahy MF, Salem R Benson Iii AB, Boucher E, et al TheraSphere Yttrium-90 Glass Microspheres Combined With Chemotherapy Versus Chemotherapy Alone in Second-Line Treatment of Patients With Metastatic Colorectal Carcinoma of the Liver: Protocol for the EPOCH Phase 3 Randomized Clinical Trial. JMIR Res Protoc. 2019 Jan 17;8(1):e11545. doi: 10.2196/11545

- Cheng A et al. Efficacy and safety of sorafenib in patients in the Asia-Pacific region with advanced hepatocellular carcinoma: a phase III randomised, double-blind, placebo-controlled trial. Lancet Oncol. 2009; 10(1): 25-34.
- Chiappa A et al. The management of colorectal liver metastases: Expanding the role of hepatic resection in the age of multimodal therapy. Crit Rev Oncol Hematol 2009; 72: 65-75
- Chiesa C et al. Need, feasibility and convenience of dosimetric treatment planning in liver selective internal radiation therapy with Y-90 microspheres: The experience of the National Cancer Institue of Milan. Q J Nucl Med Mol Imaging(55) 2011;168-197.
- Chiesa C et al. A dosimetric treatment planning strategy in radioembolisation of hepatocarcinoma with Y-90 glass microspheres. Q J Nucl Med Mol Imaging(56) 2012, 503–508.
- Chiesa et al. Radioembolization of hepatocarcinoma with 90Y glass microspheres: relationship between mean parenchyma absorbed dose and treatment related liver decompensation. European Journal of Nuclear Medicine and Molecular Imaging, 2020. Published on line. https://doi.org/10.1007/s00259-020-04845-4
- Chiesa C1, Crissien A et al. Current management of hepatocellular carcinoma. Gastroenterol Hepatol (N Y). 2014; 10(3): 153-61.
- Dieudonne A et al. (2011). Clinical Feasibility of Fast 3-Dimensional Dosimetry of the Liver for Treatment Planning of Hepatocellular Carcinoma with Y-90-microspheres. J Nucl Med(52), 1930-1937.
- Edeline J, Lenoir L, Boudjema K, et al. Volumetric changes after Y-90 radioemboisation for hepatocellular carcinoma in cirrhosis: an option to portal vein embolisation in a preoperative setting? Ann Surg Oncol. 2013; 20(8): p. 2518-2525.
- Edeline J. et al. Selective internal radiation therapy compared with sorafenib for hepatocellular carcinoma with portal vein thrombosis. Eur J Nucl Med Mol Imaging. 2016; 43(4): 635-43.
- Edeline J, Touchefeu Y, Guiu B, Farge O, Tougeron D, Baumgaertner I, Ayav A, Campillo-Gimenez B et al , Radioembolization Plus Chemotherapy for First-line Treatment of Locally Advanced Intrahepatic Cholangiocarcinoma: A Phase 2 Clinical Trial. JAMA Oncol. 2019 Oct 31. doi: 10.1001/jamaoncol.2019.3702.
- Edenvik P. et al. Application of hepatocellular carcinoma surveillance in a European setting. What can we learn from clinical practice? Liver Int. 2015; 35(7): 1862-71.
- El-Fouly A. et al. In intermediate stage hepatocellular carcinoma: radioembolisation with yttrium 90 or chemoembolisation? Liver Int. 2015; 35(2): 627-35.
- Elschot M et al. Quantitative Evaluation of Scintillation Camera Imaging Characteristics of Isotopes Used in Liver Radioembolisation PLoS One. 2011; 6: e26174.
- El-Serag H. Epidemiology of hepatocellular carcinoma in USA. Hepatol res. 2007; 37 (suppl 2): p. S88-s94.
- El-Serag H. Epidemiology of viral hepatitis and hepatocellular carcinoma. Gastroenterology. 2012; 142(6): 1264-1273.e1.
- European Association For The Study Of The Liver et al. EASL-EORTC clinical practice guidelines: management of hepatocellular carcinoma. J Hepatol. 2012; 56(4): 908-43.
- European Association for the Study of the Liver. EASL Clinical Practice Guidelines: Management of hepatocellular carcinoma. J Hepatol. 2018; 69(1):182-236
- Fartoux L et al. Carcinome hépatocellulaire : épidémiologie, physiopathologie et diagnostic. 2009; Doi: 10.1016/S1155-1976
- Ferrell B, Dow K, and Grant M. Measurement of the quality of life in cancer survivors, Quality of Life Research. 1995; 4: 523–531.
- Ferrell B, Grant M., Padilla G, Vemuri S, Rhiner, M. The experience of pain and perceptions of quality of life: validation of a conceptual model. Hospice Journal, 1991. 7: 9–24.

- Ferrell B, Wisdom C, Wenzl C. Quality of life as an outcome variable in the management of cancer pain. Cancer, 1989. 63: 2321–2327.
- Ferrell B. The impact of pain on quality of life. A decade of research, The Nursing Clinics of North America. 1995; 30: 609–624.
- Forner A, Reig M, Bruix J. Hepatocellular Carcinoma. Lancet. 2018; 391:1301-1314.
- Fukudo M. et al. Exposure-toxicity relationship of sorafenib in Japanese patients with renal cell carcinoma and hepatocellular carcinoma. Clin Pharmacokinet. 2014; 53(2): 185-96.
- Gaba R, Lewandowski R, Kulik L, et al. Radiation Lobectomy: Preliminary findings of hepatic volumetric response to lobar yttrium-90 radioembolisation. Ann Surg Oncol. 2009; 16: 1587-1596.
- Gandhi S, Khubchandani S and Lyer R. Quality of life and hepatocellular carcinoma. Journal of Gastrointestinal Oncology, 2014; 5: 296–317
- Ganten T et al. Sorafenib in patients with hepatocellular carcinoma - results of the observational INSIGHT study. Clin Cancer Res. 2017; 23(19): 5720-5728
- Garden OJ, et al. Guidelines for resection of colorectal cancer liver metastases. Gut 2006; 55 Suppl 3: iii1-iii8
- Garin E et al. Dosimetry Based on 99mTc-Macroaggregated Albumin SPECT/CT Accurately Predicts Tumor Response and Survival in Hepatocellular Carcinoma Patients Treated with Y-90-Loaded Glass Microspheres: Preliminary Results. J Nucl Med. 2012; 53: 255-263.
- Garin E, Lenoir L, Edeline J, et al. Boosted selective internal radiation therapy with Y-90-loaded galss mcirospheres (B-SIRT) for hepatocellular carcinoma patients: a new personalised promising concept. Eur J Nucl Med Mol Imaging. 2013; 40: 1057-1068.
- Garin E et al. Major impact of personalized dosimetry using 90Y loaded glass microspheres SIRT in HCC: Final overall survival analysis of a multicenter randomized phase II study (DOSISPHERE-01). Journal of Clinical Oncology 38, no. 4_suppl (February 01, 2020) 516-516
- GBD 2013 Mortality and Causes of Death Collaborators. Global, regional, and national age-sex specific all-cause and cause-specific mortality for 240 causes of death, 1990-2013: a systematic analysis for the Global Burden of Disease Study 2013. Lancet 2015; 385:117-71.
- Giannini E et al. Application of the Intermediate-Stage Subclassification to Patients With Untreated Hepatocellular Carcinoma. Am J Gastroenterol. 2016; 111(1): 70-7.
- Gill T, Feinstein A. A critical appraisal of the quality of quality-of-life measurements, The Journal of the American Medical Association. 1994; 272 (8); 619–626.
- Gmür A, Kolly P, Knöpfli M, Dufour J. FACT-Hep increases the accuracy of survival prediction in HCC patients when added to ECOG Performance Status. Liver Int. 2018 Feb 1. doi: 10.1111/liv.13711. [Epub ahead of print]
- Grothey A et al. Regorafenib monotherapy for previously treated metastatic colorectal cancer (CORRECT): an international, multicentre, randomised, placebo-controlled, phase 3 trial Lancet 2013; 381: 303–12
- Haute Autorité de Santé. Critères diagnostiques et bilan initial de la cirrhose non compliquée. 2006. https://www.has-sante.fr/portail/jcms/c_476486/fr/criteres-diagnostiques-et-bilan-initial-de-la-cirrhose-non-compliquee
- Haute Autorité de Santé. Evaluation des Implants d'embolisation artérielle (pour fistule artérioveineuse, tumeur, anévrisme) : Indications en dehors de la topographie cranioencéphalique. 2011. https://www.has-sante.fr/portail/jcms/c_1148855/fr/evaluation-des-implants-d-embolisation-arterielle-pour-fistule-arterioveineuse-tumeur-anevrisme-indications-en-dehors-de-la-topographie-cranioencephalique

- Haute Autorité de Santé. Guide Affection Longue Durée 30 – Tumeur maligne, affection maligne du tissu lymphatique ou hématopoïétique: Cancer primitif du foie. 2010. http://www.has-sante.fr/portail/jcms/c_1005121/fr/ald-n-30-cancer-primitif-du-foie
- Haute Autorité de Santé. NEXAVAR (sorafénib) : Avis de la CT en date du 5 mars 2008. https://www.has-sante.fr/portail/jcms/c_642569/fr/nexavar?xtmc=&xtcr=2
- Haute Autorité de Santé. Radiothérapie en conditions stéréotaxiques des tumeurs hépatiques - Rapport d'évaluation technologique. 2016. https://www.has-sante.fr/portail/jcms/c_2565031/fr/radiotherapie-en-conditions-stereotaxiques-des-tumeurs-hepatiques-rapport-d-evaluation-technologique
- Haute Autorité de Santé SIR-Spheres, Microsphères d'Yttrium-90 : Avis de la CNEDiMTS en date du 24 mars 2015.
- Haute Autorité de Santé. TheraSphere, Microsphères d'Yttrium-90 : Avis de la CNEDiMTS en date du 20 février 2018. https://www.hasante.fr/portail/jcms/c_2831678/fr/therasphere?xtmc=&xtcr=264
- Haute Autorité de Santé TheraSphere, Microsphères d'Yttrium-90 : Avis de la CNEDiMTS en date du 31 mai 2011. Health outcomes methodology. Medical Care. 2000; 38(9 Suppl II): II7–II13.
- Hickey R et al 90Y Radioembolization of Colorectal Hepatic Metastases Using Glass Microspheres: Safety and Survival Outcomes from a 531-Patient Multicenter Study. J Nucl Med 2016; 57:665–671
- Hilgard P, Hamami M, Fouly A, et al. Radioembolisation with Yttrium-90 glass microspheres in hepatocellular carcinoma: European experience on safety and long-term survival. Hepatology. 2010; 52: p. 1741-1749.
- Ho S, et al. Partition model for estimating radiation doses from yttrium-90 microspheres in treating hepatic tumours. Eur J Nucl Med. 1996; 23: 947-952.
- Holmes S. Measuring health related quality of life in patients with hepatobiliary cancers: the functional assessment of cancer therapy-hepatobiliary questionnaire. Journal of Clinical Oncology. 2002; 20: 2229–39.
- https://www.btg-im.com/en-GB/TheraSphere/Products/Indications
- https://www.has-sante.fr/portail/jcms/c_2831678/fr/therasphere
- Hubert M, et al. Beyond budget silos: budget impact analysis of transarterial radioembolisation with yttrium-90 glass microspheres for hepatocellular carcinoma from a hospital perspective. Value in Health. 2016; 19(3): A308. http://www.valueinhealthjournal.com/article/S1098-3015(16)00739-7/abstract
- Idée J, et al. Use of Lipiodol as a drug-delivery system for transcatheter arterial chemoembolisation of hepatocellular carcinoma: a review. Crit Rev Oncol Hematol. 2013; 88(3): 530-49.
- Ikai I, Kudo M, Arii S, Omata M, Kojiro M, Sakamoto M, Takayasu K, Hayashi N, Makuuchi M, Matsuyama Y et al. Report of the 18th follow-up survey of primary liver cancer in Japan. Hepatol Res. 2010; 40: 1043–1059.
- Institut National Du Cancer. Définition traitement palliatif. http://www.e-cancer.fr/Dictionnaire/T/ traitement-palliatif
- Johnson P et al. Assessment of liver function in patients with hepatocellular carcinoma: a new evidence-based approach to the ALBI grade. J Clin Oncol 2015; 33: 550–558.
- Kennedy A et al. Recommendations for radioembolisation of hepatic malignancies using yttrium-90 microsphere brachytherapy: a consensus panel report from the radioembolisation brachytherapy oncology consortium. Int J Radiat Oncol Biol Phys. 2007; 68(1): 13-23.

- Kennedy A et al. Regorafenib Prior to Selective Internal Radiation Therapy Using 90Y-Resin Microspheres for Refractory Metastatic Colorectal Cancer Liver Metastases: Analysis of Safety, Dosimetry, and Molecular Markers. Front Oncol. 2019 10;9:624.
- Kennedy A et al. Updated survival outcomes and analysis of long-term survivors from the MORE study on safety and efficacy of radioembolization in patients with unresectable colorectal cancer liver metastases. J Gastrointest Oncol. 2017 Aug;8(4):614-624.
- Kim J et al. New intermediate-stage subclassification for patients with hepatocellular carcinoma treated with transarterial chemoembolisation. Liver Int. 2017; 37(12): 1861-1868
- Kimura H et al. Subclassification of patients with intermediate-stage (Barcelona Clinic Liver Cancer stage-B) hepatocellular carcinoma using the up-to-seven criteria and serum tumor markers. Hepatol Int. 2017; 11(1): 105-14.
- Klein J et al. Stereotactic body radiotherapy: an effective local treatment modality for hepatocellular carcinoma. Future Oncol. 2014; 10(14): 2227-41.
- Knesaurek K et al. Quantitative Comparison of Yttrium-90(Y-90)-Microspheres and Technetium-99m (99mTc)-Macroaggregated Albumin SPECT Images for Planning Y-90 Therapy of Liver Cancer. Technol Cancer Res Treat. 2010; 9; 253-262.
- Kokabi N et al. Open-label prospective study of the safety and efficacy of glass-based yttrium 90 radioembolisation for infiltrative hepatocellular carcinoma with portal vein thrombosis. Cancer. 2015; 121(13): 2164-74.
- Kokudo T et al. Survival benefit of liver resection for hepatocellular carcinoma associated with portal vein invasion. J Hepatol. 2016; 65(5): 938-43.
- Ksienski D, et al. Patterns of referral and resection among patients with liver-only metastatic colorectal cancer (MCRC). Ann Surg Oncol 2010; 17: 3085-3093
- Kulik L, Atassi B, Van Holsbeeck L et al. Yttrium-90 microspheres (TheraSphere) treatment of unresectable hepatocellular carcinoma: Downstaging to resection, RFA and bridget to transplantation. J Surg Oncol. 2006; 94: 572-586.
- Lam M et al. Limitations of Body Surface Area-Based Activity Calculation for Radioembolisation of Hepatic Metastases in Colorectal Cancer J Vasc interv Radiol. 2014; 25: 1085-1093.
- Lambert B et al. Intra-arterial treatment with Y-90 microspheres for hepatocellular carcinoma: 4 years experience at the Ghent University Hospital. Eur J Nucl Med Mol Imaging. 2011; 38(12): 2117-24.
- Lammer J et al. Prospective randomised study of doxorubicin-eluting-bead embolisation in the treatment of hepatocellular carcinoma: results of the PRECISION V study. Cardiovasc Intervent Radiol. 2010; 33(1): 41-52.
- Lance C et al. Comparative analysis of the safety and efficacy of transcatheter arterial chemoembolisation and yttrium-90 radioembolisation in patients with unresectable hepatocellular carcinoma. J Vasc Interv Radiol. 2011; 22(12): 1697-705.
- Lea WB et al. Microsphere Localisation and Dose Quantification Using Positron Emission Tomography/CT following Hepatic Intraarterial Radioembolisation with Yttrium-90 in Patients with Advanced Hepatocelluar Carcinoma J Vasc Interv Radiol. 2014; 25; 1595-1603.
- Lewandowski R et al. Yttrium-90 radioembolisation of hepatocellular carcinoma and metastatic disease to the liver. Semin Intervent Radiol. 2006; 23(1): 64-72.
- Lewandowski R, Kulik L, Riaz A, et al. A comparative analysis of transarterial downstaging for hepatocellular carcinoma: Chemoembolisation versus radioembolisation. Am J Transplant. 2009; 9: 1920-1928.
- Liver cancer statistics: World Cancer Research Fund International. http://www.wcrf.org/int/cancer-facts-figures/data-specific-cancers/liver-cancer-statistics

- Llovet J et al. Sorafenib in advanced hepatocellular carcinoma. N Engl J Med. 2008; 359(4): 378-90.
- Llovet J et al. Systematic review of randomised trials for unresectable hepatocellular carcinoma: Chemoembolisation improves survival. Hepatology. 2003; 37(2): 429-42.
- Lo C et al. Randomised Controlled Trial of Transarterial Lipiodol Chemoembolisation for Unresectable hepatocellular Carcinoma Hepatology. 2002; 35: 1164-1171.
- Mahnken A et al. Standards of practice in transarterial radioembolisation. Cardiovasc Intervent Radiol. 2013; 36(3): 613-22.
- Manceau V, Palard X, Rolland Y, Pracht M, Le Sourd S, Laffont S, Boudjema K, Lievre A, Mesbah H, Haumont LA, Lenoir L, Brun V, Uguen T, Edeline J, Garin E. A MAA-based dosimetric study in patients with intrahepatic cholangiocarcinoma treated with a combination of chemotherapy and (90)Y-loaded glass microsphere selective internal radiation therapy. Eur J Nucl Med Mol Imaging. 2018;45(10):1731-1741.
- Marrero J.A. et al. ACG clinical guideline: the diagnosis and management of focal liver lesions. Am J Gastroenterol. 2014; 109(9): 1328-1347; 1348.
- Mason M et al. Post-embolisation syndrome as an early predictor of overall survival after transarterial chemoembolisation for hepatocellular carcinoma. HPB (Oxford). 2015; 17(12): 1137-44.
- Mazzaferro V et al. Yttrium-90 radioembolisation for intermediate-advanced hepatocellular carcinoma: a phase 2 study. Hepatology. 2013; 57(5): 1826-37.
- McGowan C.E. et al. Suboptimal surveillance for and knowledge of hepatocellular carcinoma among primary care providers. Clin Gastroenterol Hepatol. 2015; 13(4): 799-804.
- Memon K et al. Radioembolisation for hepatocellular carcinoma with portal vein thrombosis: impact of liver function on systemic treatment options at disease progression. J Hepatol. 2013; 58(1): 73-80.
- Meyer T et al. A randomised phase II/III trial of 3-weekly cisplatin-based sequential transarterial chemoembolisation vs embolisation alone for hepatocellular carcinoma. Br J Cancer. 2013; 108(6): 1252-9.
- Moreno-Luna L.E. et al. Efficacy and safety of transarterial radioembolisation versus chemoembolisation in patients with hepatocellular carcinoma. Cardiovasc Intervent Radiol. 2013; 36(3): 714-23.
- Moriguchi M. A Review of Non-operative Treatments for Hepatocellular Carcinoma with Advanced Portal Vein Tumor Thrombus. Journal of Clinical and Translational Hepatology. 2017; 5(2): 177-183.
- Mosconi C et al. Radioembolisation with Yttrium-90 microspheres in hepatocellular carcinoma: Role and perspectives. World J Hepatol. 2015; 7(5): 738-52.
- Mulcahy MF, Lewandowski RJ, Ibrahim SM, Sato KT, Ryu RK, Atassi B, Newman S, Talamonti M, Omary RA, Benson A 3rd, Salem R. Radioembolization of colorectal hepatic metastases using yttrium-90 microspheres. Cancer. 2009 May 1;115(9):1849-58. PubMed PMID: 19267416.
- National Cancer Institute. Cancer Stat Facts: Liver and Intrahepatic Bile Duct Cancer: https://seer.cancer.gov/statfacts/html/livibd.html
- National Cancer Institute. NCI Common Terminology Criteria for Adverse Events (CTCAE) v.4 data files: https://evs.nci.nih.gov/ftp1/CTCAE/About.html
- National Institute for Health and Care Excellence. Selective internal radiation therapy for primary hepatocellular carcinoma - Guidance and guidelines. 2013.
- Nezami N, Kokabi N, Camacho JC, Schuster DM, Xing M, Kim HS. (90)Y radioembolization dosimetry using a simple semi-quantitative method in

intrahepatic cholangiocarcinoma: Glass versus resin microspheres. Nucl Med Biol. 2018;59:22-28

- Nordenstedt H, white D, El-Serag H. The changing pattern of epidemiology in hepatocellular carcinoma. Dig liver dis. 2010; 42 (suppl 3): S206-s214.
- Oken M et al. Toxicity and response criteria of the Eastern Cooperative Oncology Group. Am J Clin Oncol. 1982; (5): 649-655.
-
- Padia SA et al. Comparison of Positron Emission Tomography and Bremsstrahlung Imaging to Detect Particle Distribution in Patients Undergoing Yttrium-90 Radioembolisation for Large Hepatocellular Carcinomas or Associated Portal Vein Thrombosis J Vasc Interv Radiol. 2013; 24: 1147-1153.
- Pellino A, Loupakis F, Cadamuro M, et al. Precision medicine in cholangiocarcinoma. Transl Gastroenterol Hepatol 2018; 3:40.
- Piscaglia F et al. The intermediate hepatocellular carcinoma stage: Should treatment be expanded? Dig Liver Dis. 2010; 42 Suppl 3: S258-263.
- Pracht M et al. Lobar hepatocellular carcinoma with ipsilateral portal vein tumor thrombosis treated with yttrium-90 glass microsphere radioembolisation: preliminary results. Int J Hepatol. 2013; 2013: 827649.
- Raoul J et al. Evolving strategies for the management of intermediate-stage hepatocellular carcinoma: available evidence and expert opinion on the use of transarterial chemoembolisation. Cancer Treat Rev. 2011; 37(3): 212-20.
- Raoul J et al. Treatment of hepatocellular carcinoma with intra-arterial injection of radionuclides. Nat Rev Gastroenterol Hepatol. 2010; 7(1): 41-9.
- Riaz A, Gates V, Atassi B, et al. Radiation segmentectomy: A novel approach to increase safety and efficacy of radioembolisation. Int J Radiation Oncology Biol Phys. 2011; 79(1): 163-171.
- Rizvi S, Gores GJ. Emerging molecular therapeutic targets for cholangiocarcinoma.J Hepatol 2017; 67:632-44.
- Rizvi S, Gores GJ. Pathogenesis, diagnosis, and management of cholangiocarcinoma. Gastroenterology 2013; 145:1215-29.
- Robert J. Mayer, Randomized Trial of TAS-102 for Refractory Metastatic Colorectal Cancer N Engl J Med 2015;372:1909-19.
- Rognoni C. et al. Real-World Data for the Evaluation of Transarterial Radioembolisation versus Sorafenib in Hepatocellular Carcinoma: A Cost-Effectiveness Analysis. Value Health. 2017; 20(3): 336-44.
- Salem R et al. Institutional decision to adopt Y-90 as primary treatment for hepatocellular carcinoma informed by a 1,000-patient 15-year experience. Hepatology. 2017 Dec 1. doi: 10.1002/hep.29691 [Epub ahead of print].
- Salem R et al. Y-90 Radioembolisation Significantly Prolongs Time to Progression Compared With Chemoembolisation in Patients With Hepatocellular Carcinoma. Gastroenterology. 2016; 151(6): 1155-1163.e2.
- Salem R, Lewandowski R, Mulcahy M et al. Radioembolisation for hepatocellular carcinoma using Yttrium-90mMicrospheres: A comprehensive report of long-term outcomes. Gastroenterology. 2010; 138(1): 52-64.
- Salem R, Lewandowski RJ, Kulik L et al. Radioembolisation results in longer time-to-progression and reduced toxicity compared with chemoembolisation in patients with hepatocellular carcinoma. Gastroenterology. 2011; 140(2): 491-507.

- Salem R. et al. Increased quality of life among hepatocellular carcinoma patients treated with radioembolisation, compared with chemoembolisation. Clin Gastroenterol Hepatol. 2013; 11(10): 1358-1365.e1.
- Salem R. et al. Radioembolisation with Y-90ttrium microspheres: a state-of-the-art brachytherapy treatment for primary and secondary liver malignancies. Part 1: Technical and methodologic considerations. J Vasc Interv Radiol. 2006; 17(8): 1251-78.
- Salem, R., Padia, S. A., Lam, M., Bell, J., Chiesa, C., Fowers, K., Hamilton, B., Herman, J., Kappadath, S. C., Leung, T., Portelance, L., Sze, D., & Garin, E. (2019). Clinical and dosimetric considerations for Y90: recommendations from an international multidisciplinary working group. European Journal of Nuclear Medicine and Molecular Imaging, 46(8), 1695-1704.
- Shi J et al. A new classification for hepatocellular carcinoma with portal vein tumor thrombus. J Hepatobiliary Pancreat Sci. 2011; 18(1): 74-80.
- Shuqun C et al. Tumor thrombus types influence the prognosis of hepatocellular carcinoma with the tumor thrombi in the portal vein. Hepatogastroenterology. 2007; 54(74): 499-502.
- Société Nationale Française de Gastro-Entérologie. Thesaurus National de Cancérologie Digestive (TNCD) - Carcinome hépatocellulaire (cancer primitif du foie). 2015. http://www.snfge.org/content/7-carcinome-hepatocellulaire-cancer-primitif-du-foie
- Strigari L et al. The evidence base for the use of internal dosimetry in the clinical practice of molecular radiotherapy. Eur J Nucl Med Mol Imaging. 2014; Epub; 11Jun14.
- TheraSphere™ Yttrium-90 Glass Microspheres Instruction for Use.
- Theysohn J, Ertle J, Muller S, et al. Hepative volume changes after lobal selective internal radiation therapy (SIRT) of hepatocellular carcinoma. Clin Radiol. 2014; 172-178.
- Torre la Bray F, Siegel R, et al. Global cancer statistics. 2012. Ca Cancer J Clin. 2015; 65 (2): 87-108.
- U.S. Department of Health and Human Services. Organ Procurement and Transplantation Network. Available from: http://optn.transplant.hrsa.gov/news/revised-liver-hcc-exception-scores.
- Valle J, Wasan H, Palmer DH, et al. Cisplatin plus gemcitabine versus gemcitabine for biliary tract cancer. N Engl J Med. 2010;362(14):1273–1281.
- Van Cutsem E, et al.. Fluorouracil, Leucovorin, and Irinotecan Plus Cetuximab Treatment and *RAS* Mutations in Colorectal Cancer. Journal of Clinical Oncology 2015 33:7, 692-700
- Van den Eynde M, et al. Treatment of colorectal liver metastases: a review. Rev Recent Clin Trials 2009; 4: 56-62
- Vouche M et al. Unresectable Solitary Hepatocellular Carcinoma Not Amenable to Radiofrequency Ablation: Multicentre Radiology-Pathology Correlation and Survival of Radiation Segmentectomy Hepatology. 2014; 60; 192-201.
- Vouche M, Habib A, Ward T et al. Unresectable solitary hepatocellular carcinoma not amenable to radiofrequency ablation: Multicentre radiology-pathology correlation and survival of radiation segmentectomy. Hepatology. 2014; 60(1): 192-201.
- Vouche M, Lewandowski R, Atassi R, et al. Radiation lobectomy: Time-dependent analysis of future liver remnant volume in unresectable liver cancer as a bridge to resection. J Hepatol. 2013; 59(5): 1029-1036.
- Walrand S et al. A Hepatic Dose-Toxicity Model Opening the Way Toward Individualised Radioembolisation Planning J Nucl Med. 2014; (55), 1-6.
- WHO Cancer. WHO. http://www.who.int/mediacentre/factsheets/fs297/en/
- Williet N et al. Tolerance and outcomes of sorafenib in elderly patients treated for advanced hepatocellular carcinoma. Dig Liver Dis. 2017; 49(9): 1043-1049

- Wondergem M et al. 99mTc-Macroaggregated Albumin Poorly Predicts the Intrahepatic Distribution of Y-90 Resin Microspheres in Hepatic Radioembolisation J Nucl Med. 2013; 54; 1294-1301
- Ziogas D et al. Efficacy and safety of sorafenib in patients with advanced hepatocellular carcinoma: age is not a problem. Eur J Gastroenterol Hepatol. 2017; 29(1): 48-55.

DocuSign SECURED

Certificate Of Completion

Envelope Id: 15E39A4A85F9439289AF9B6AE19B0B79 | Status: Completed

Subject: Please DocuSign: PROACTIF Protocol V4.0 30Jun20 Clean.docx

Source Envelope:

Document Pages: 91	Signatures: 6	Envelope Originator:
Certificate Pages: 5	Initials: 0	Kevin Manning
AutoNav: Enabled		Chapman House Farnham Business Park
EnvelopeId Stamping: Disabled		Weydon Lane, Farnham
Time Zone: (UTC) Dublin, Edinburgh, Lisbon, London		Surrey, Surrey GU9 8QL
		kevin.manning@btgplc.com
		IP Address: 37.200.118.76

Record Tracking

Status: Original<br>01-Jul-2020 | 09:07	Holder: Kevin Manning<br>kevin.manning@btgplc.com	Location: DocuSign

Signer Events	Signature	Timestamp
Binal Patel<br>binal.patel@bsci.com<br>Binal Patel<br>Security Level: Email, Account Authentication (Required)	Binal Patel<br><br>Signature Adoption: Pre-selected Style<br>Signature ID:<br>ABEC9833-6D00-4412-98B4-B00DAC0BE220<br>Using IP Address: 72.92.22.52<br><br>With Signing Authentication via DocuSign password<br>With Signing Reasons (on each tab):<br>I approve this document	Sent: 01-Jul-2020 | 09:10<br>Viewed: 01-Jul-2020 | 12:52<br>Signed: 01-Jul-2020 | 12:52
**Electronic Record and Signature Disclosure:**<br>Accepted: 16-Mar-2020 | 14:29<br>ID: 8999c396-d4fe-4622-94e0-f595a94ed9e9		
Eveline Boucher<br>Evelyne.Boucher@bsci.com<br>Evelyne.Boucher<br>Security Level: Email, Account Authentication (Required)	Eveline Boucher<br><br>Signature Adoption: Pre-selected Style<br>Signature ID:<br>D1BA4A32-3767-41E0-AF8A-3FED7F22E389<br>Using IP Address: 83.199.73.102<br><br>With Signing Authentication via DocuSign password<br>With Signing Reasons (on each tab):<br>I approve this document	Sent: 01-Jul-2020 | 09:10<br>Viewed: 01-Jul-2020 | 09:35<br>Signed: 01-Jul-2020 | 09:35
**Electronic Record and Signature Disclosure:**<br>Accepted: 20-Mar-2020 | 12:43<br>ID: 38a81c85-4f81-4768-9015-538f5d7e2357		

Signer Events	Signature	Timestamp
Henk Tissing<br>hendrik.tissing@bsci.com<br>VP, IO Clinical Development<br>HendrikTissing<br>Security Level: Email, Account Authentication (Required)	Henk Tissing<br><br>Signature Adoption: Pre-selected Style<br>Signature ID:<br>4A8BA900-BBCB-479F-9CFC-6635678E560B<br>Using IP Address: 217.103.115.91<br><br>With Signing Authentication via DocuSign password<br>With Signing Reasons (on each tab):<br>I approve this document	Sent: 01-Jul-2020 | 09:10<br>Viewed: 01-Jul-2020 | 09:12<br>Signed: 01-Jul-2020 | 09:14
**Electronic Record and Signature Disclosure:**<br>Accepted: 16-Apr-2020 | 11:27<br>ID: c1041783-2c77-416f-8fc7-455d24133b7a		
Robert White<br>Robert.White@bsci.com<br>Robert White<br>Security Level: Email, Account Authentication (Required)	Robert White<br><br>Signature Adoption: Pre-selected Style<br>Signature ID:<br>85E90A4A-6670-40E3-8DAD-C418CE08564E<br>Using IP Address: 86.25.146.211<br><br>With Signing Authentication via DocuSign password<br>With Signing Reasons (on each tab):<br>I approve this document	Sent: 01-Jul-2020 | 09:10<br>Viewed: 01-Jul-2020 | 09:11<br>Signed: 01-Jul-2020 | 09:12
**Electronic Record and Signature Disclosure:**<br>Accepted: 24-Jun-2020 | 13:25<br>ID: 76cd81a5-95ca-4c93-bdbc-5a3864928a64		
Boris Guiu<br>b-guiu@chu-montpellier.fr<br>Security Level: Email, Account Authentication (Required), Authentication	Boris Guiu<br><br>Signature Adoption: Pre-selected Style<br>Signature ID:<br>BD404409-9779-44BB-B152-55445EF5B3D3<br>Using IP Address: 81.14.60.200<br><br>With Signing Authentication via DocuSign password<br>With Signing Reasons (on each tab):<br>I approve this document	Sent: 01-Jul-2020 | 12:52<br>Viewed: 02-Jul-2020 | 12:43<br>Signed: 02-Jul-2020 | 12:44
**Authentication Details**<br>SMS Auth:<br>Transaction: 35CC3D5A1F700C049190CEB3945A41FF<br>Result: passed<br>Vendor ID: TeleSign<br>Type: SMSAuth<br>Performed: 02-Jul-2020 | 12:43<br>Phone: +33 6 30 57 91 49		
**Electronic Record and Signature Disclosure:**<br>Accepted: 01-May-2019 | 08:57<br>ID: 537fdafc-9053-439c-9546-011cf8ba86b3		

Signer Events	Signature	Timestamp
Etienne Garin<br>e.garin@rennes.unicancer.fr<br>MD, PhD, Global Investigator<br>Security Level: Email, Account Authentication (Required), Authentication	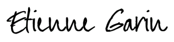<br>Signature Adoption: Pre-selected Style<br>Signature ID:<br>38646D40-1DC7-4434-8A36-205A7610C03D<br>Using IP Address: 2.10.13.187<br><br>With Signing Authentication via DocuSign password<br>With Signing Reasons (on each tab):<br>I approve this document	Sent: 01-Jul-2020 | 12:52<br>Viewed: 01-Jul-2020 | 16:34<br>Signed: 01-Jul-2020 | 16:35

Authentication Details
SMS Auth:
Transaction: 35CC2C0C226413049195F029A5EA186A
Result: passed
Vendor ID: TeleSign
Type: SMSAuth
Performed: 01-Jul-2020 | 16:33
Phone: +33 6 30 17 64 15

Electronic Record and Signature Disclosure:
Accepted: 06-May-2019 | 07:30
ID: c8142a09-2bc2-4020-b4d7-f41e4a74f9f1

In Person Signer Events	Signature	Timestamp

Editor Delivery Events	Status	Timestamp

Agent Delivery Events	Status	Timestamp

Intermediary Delivery Events	Status	Timestamp

Certified Delivery Events	Status	Timestamp

Carbon Copy Events	Status	Timestamp
Sarah Cooper<br>sarah.cooper@btgplc.com<br>Senior Manager, Clinical Development<br>BTG (Default Signer)<br>Security Level: Email, Account Authentication (Required)	COPIED	Sent: 01-Jul-2020 | 09:10<br>Viewed: 01-Jul-2020 | 12:15

Electronic Record and Signature Disclosure:
Accepted: 29-Jun-2020 | 19:11
ID: d28dc18f-ef2a-4220-b9b4-84223b559f9b

Witness Events	Signature	Timestamp

Notary Events	Signature	Timestamp

Envelope Summary Events	Status	Timestamps
Envelope Sent	Hashed/Encrypted	01-Jul-2020 | 12:52
Certified Delivered	Security Checked	02-Jul-2020 | 12:43
Signing Complete	Security Checked	02-Jul-2020 | 12:44
Completed	Security Checked	02-Jul-2020 | 12:44

Payment Events	Status	Timestamps

Electronic Record and Signature Disclosure

By Signing this disclosure, I agree that my electronic signature is the legally binding equivalent to my handwritten signature. Whenever I execute an electronic signature using DocuSign for BTG documents, it has the same validity and meaning as my handwritten signature. I will not, at any time repudiate the meaning of my electronic signature or claim that my electronic signature is not legally binding.

By Signing this disclosure, I agree that my electronic signature is the legally binding equivalent to my handwritten signature. Whenever I execute an electronic signature using DocuSign for BTG documents, it has the same validity and meaning as my handwritten signature. I will not, at any time repudiate the meaning of my electronic signature or claim that my electronic signature is not legally binding.
